# Self‐assembly of chiral diketopyrrolopyrrole chromophores giving supramolecular chains in monolayers and twisted microtapes

**DOI:** 10.1002/chir.23539

**Published:** 2023-02-09

**Authors:** Joshua Humphreys, C. Elizabeth Killalea, Flavia Pop, E. Stephen Davies, Giuliano Siligardi, David B. Amabilino

**Affiliations:** ^1^ The GSK Carbon Neutral Laboratories for Sustainable Chemistry The University of Nottingham Jubilee Campus Nottingham UK; ^2^ School of Chemistry University of Nottingham Nottingham UK; ^3^ Diamond Light Source, Harwell Science and Innovation Campus Didcot Oxfordshire UK; ^4^ Institut de Ciència de Materials de Barcelona (ICMAB‐CSIC) Campus Universitari de Cerdanyola Barcelona Spain; ^5^ Present address: MOLTECH‐Anjou, UMR 6200, CNRS University of Angers Angers France

**Keywords:** aggregation, hydrogen bonds, morphology, scanning tunnelling microscopy

## Abstract

Chiral diketopyrrolopyrroles appended with enantiomeric ethyl lactate functions through an ether linkage to the aryl backbone of the chromophore were synthesized via the Mitsunobu reaction. The molecules have good solubility and excellent optical properties, high molar absorption coefficients, and fluorescence quantum yields. Helical aggregates with circular dichroism arising from the supramolecular arrangement are seen in both solution and thin films, and the aggregates also display circularly polarized luminescence (g_lum_ ≈ ±0.1). The molecules assemble to give monolayers on graphite and precipitate from solution forming supramolecular twisted tapes hundreds of microns long.

## Graphical abstract

Chiral diketopyrrolopyrroles assemble to give monolayers on graphite and precipitate from solution forming supramolecular twisted tapes hundreds of microns long. The chirality in the films is revealed using circular dichroism imaging.

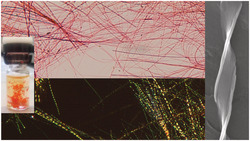

## 1 INTRODUCTION

A supramolecular approach to the design of organic electronic materials has been proposed and investigated for well over two decades as a strategy to produce highly efficient optoelectronic devices.^1^, ^2^, ^3^, ^4^, ^5^, ^6^, ^7^, ^8^ At the basic level, there are two key areas that this approach looks to improve upon regarding existing technology: active layer morphology and charge carrier transport. In organic solar cells, morphological control ideally sees the checkerboard pattern employed,^9^, ^10^ which would see large contact between donor and acceptor domains allowing exciton splitting into free charge carriers and efficient charge transport to electrodes. Non‐covalent interactions, namely, π stacking^11^ and hydrogen bonding,^4^, ^12^ have been shown to influence charge mobility in organic materials, and incorporation of these motifs onto chromophores, coupled with the introduction of chirality, has seen the manifestation of a number of superstructure architectures from liquid crystals^13^ to gelators,^14^ ribbons,^15^ nanocrystals,^16^ and nanowires.^17^ Hence, the design of supramolecular electronic materials looks to exploit this ordering to create reproducible, idealistic active layer morphologies between donor and acceptor domains as a means to improve charge transport and efficiency in next‐generation optoelectronic devices.^18^, ^19^

In the context of organic semiconductors, over recent years, diketopyrrolopyrrole (DPP) has come to the fore as a well‐explored material, with simple synthetic transformations resulting in substantial modulation of properties.^20^, ^21^ This has led to the development of highly efficient organic electronic devices ranging from solar cells to transistors to organic light emitting diodes (OLEDs).^22^, ^23^, ^24^, ^25^, ^26^ Broadly speaking, the aryl substituent, in the most part, typically dictates the intended application of the DPP molecule, with thiophene‐based systems (**ThDPP,** Supporting information S1: ESI 5.1) being used for organic electronic applications thanks to their planarity, good charge transport, and straightforward polymerization, whereas phenyl‐based systems (**PhDPP**, Supporting information S1: ESI 5.1) are synonymous with sensors and imaging agents given their excellent emissive properties in both the solution and the solid state.^27^, ^28^, ^29^, ^30^, ^31^, ^32^, ^33^, ^34^, ^35^, ^36^, ^37^

For DPP systems, there have been several strategies to impart hydrogen bonding^38^ and chiral motifs^39^, ^40^, ^41^ that promote self‐assembly and modulate π–π stacking interactions, to ultimately evaluate their impact on the compounds' optoelectronic performance. DPPs inherently suffer from solubility issues given the strong intermolecular interactions driven by π–π stacking and hydrogen bonding through the lactam moiety. Hence, to study hydrogen bonding in solution‐processable organic electronic devices, much of the early work in this area looked to use thermocleavable groups attached at the lactam nitrogen that would increase solubility to allow thin film deposition from solution followed by thermal annealing to release the hydrogen‐bonding network.^42^, ^43^, ^44^, ^45^, ^46^ Mono‐alkylation of the DPP unit has been explored also, with these more soluble derivatives displaying cofacial π stacks that show improved mobility versus the herringbone structure often adopted by the dialkylated form.^47^, ^48^ Additionally, the introduction of alkyl chains containing hydrogen‐bonding amide and urea groups at the lactam nitrogen has been reported.^46^, ^49^, ^50^, ^51^, ^52^, ^53^, ^54^ Alternative functionalization along the aryl backbone through the Knoevenagel condensation reaction with amide and semicarbazone motifs has been described for **ThDPP**‐based systems.^55^, ^56^, ^57^, ^58^ With regard to chirality, alkyl, amide, and amino acid side chains have been typically introduced through *N‐*alkylation at the lactam.^59^, ^60^, ^61^, ^62^, ^63^, ^64^ Additionally, end capping of the aryl system to give axially chiral systems with promising circularly polarized luminescence (CPL) properties has been detailed.^65^, ^66^, ^67^, ^68^ Imparting said motifs that promote self‐assembly on to the DPP skeleton has seen systems that form gels,^62^, ^69^, ^70^ nanowires,^71^ and liquid crystals^72^, ^73^ among others.

Despite the generalization that **PhDPP** systems are less suited as standalone optoelectronic materials relative to **ThDPP**, Glowacki *et al.* previously investigated **PhDPP *N*H** semiconductors and suggested promising mobility values.^34^ Little has been explored since, possibly because of the molecule's poor solubility. For **PhDPP**, *N*‐alkylation leads to soluble, highly emissive materials, at the expense of forming twisted molecular backbones (attributed to the steric clash between the phenyl‐appended protons and the *N‐*substituents) that have reduced mobility, absorption, and large band gaps relative to their parent chromophore.^74^ They are generally regarded as being less favorable as standalone materials in devices but potentially interesting as morphological additives.^70^, ^75^ From a theoretical perspective, it has been suggested that, based on calculated mobilities from **PhDPP**‐based crystal structures, these systems hold promise to rival rubrene as organic field effect transistor (OFET) materials, with the challenge of optimization of device engineering the main obstacle.^75^ In light of this hypothesis, and taking into account that retention of the free lactam and functionalizing along the backbone with phenyl ethers has seen derivatives with improved solubility that self‐assemble to form lamellar homo assemblies dominated by hydrogen bonding between lactams,^72^, ^73^, ^76^, ^77^ we proposed potentially functionalizing the DPP backbone with chiral esters to impart solubility and investigate the influence of chirality and hydrogen bonding of the lactam core on the supramolecular assembly of the system as a means to potentially address the challenge of active layer morphological control and charge transport in organic electronics. Thus, herein, we describe the synthesis of a novel DPP system appended with enantiomeric non‐racemic ethyl lactate functions through an ether linkage and characterize the optoelectronic properties and self‐assembly in the solution, at an interface, and in the solid‐state, to aid in development of future supramolecular organic electronic materials.

## 2 RESULTS AND DISCUSSION

### 2.1 Synthetic procedure

**SCHEME 1:**
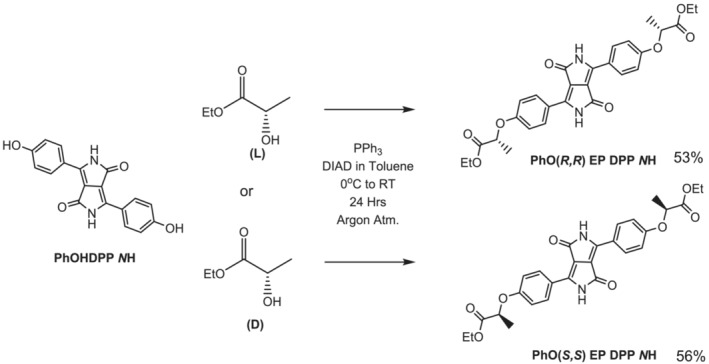
Reaction conditions used for the synthesis of **PhO(*R,R*) EP DPP *N*H** and **PhO(*S,S*) EP DPP *N*H**.

The core phenol‐appended DPP compound (**PhOHDPP *N*H**) was synthesized according to previously published methods.^73^, ^78^ Once isolated, we wished to functionalize the phenol moiety with chiral substituents to introduce asymmetry, increase solubility, and maintain the ability of the core to enter into hydrogen bonding. The chain used here is ethyl lactate, which is commercially available as both enantiomers and can be post‐functionalized to create other chiral hydrogen‐bonding materials such as acids (Supporting information S1: ESI 5.2) and amides. The (−)‐ethyl L‐lactate (*S* enantiomer) was explored first, because as the natural isomer it is cheaper. The proposed route to achieve the ether formation was via the Mitsunobu reaction,^79^, ^80^ to ensure stereoselectivity and avoid basic reagents that might react with the lactam *N*H, which leads to undesired products.^73^ We, among many, have already shown that this reaction is highly stereoselective with enantiomeric excesses typically over 90 for this kind of phenol‐lactate coupling.^81^, ^82^, ^83^ To optimize the reaction, fresh bottles of ethyl lactate and coupling agent were used, and the solvent dried exhaustively, and purification by Soxhlet extraction followed by precipitation was required to maximize the purity in one case. We were unable to identify the impurity after only precipitation (although it does affect the optical activity, see below). After optimization, the reaction (Scheme 1) proceeded well to produce the novel chiral system **PhO(*R,R*) EP DPP *N*H** in good yield (53%), with inversion of stereochemistry. The solubility of the compound at room temperature is good in solvents such as dimethylsulphoxide (DMSO), *N,N'*‐dimethylformamide (DMF), diglyme, and tetrahydrofuran (THF). The opposite enantiomer (**PhO[*S,S*] EP DPP *N*H**) was also synthesized, using (+)‐ethyl‐D‐lactate (*R* enantiomer), under identical conditions (Scheme 1). The compounds showed better solubility compared with the parent compound **PhOHDPP *N*H**. Although we were unable to check the enantiomeric excess of the chiral compounds made here (their high polarity and their limited solubility in appropriate solvents for chromatography made this impractical in our hands), the optical activity observed by circular dichroism (CD) spectroscopy indicates similar enantiopurity (see below). Despite appearing clean by nuclear magnetic resonance spectroscopy (NMR) and elemental analysis, the first attempts at synthesis of **PhO(*R,R*) EP DPP NH** and **PhO(*S,S*) EP DPP NH** resulted in compounds that exhibited slightly different CD spectra (and only **PhO(*S,S*) EP DPP NH** gave rise to a CPL signal in the first isolated materials, see below), indicating a slight chemical or optical impurity.^84^, ^85^ The fact that after optimization the compounds were purified by precipitation, a process that would be expected to favor a high enantiomeric purity, and the process does give materials with mirror image CD spectra, supports that hypothesis. The subsequent characterization proved the identity and chemical purity of the compounds (see Supporting information S1: ESI for details).

### 2.2 Optical properties

#### 2.2.1 Solution‐state absorption properties

**FIGURE 1:**
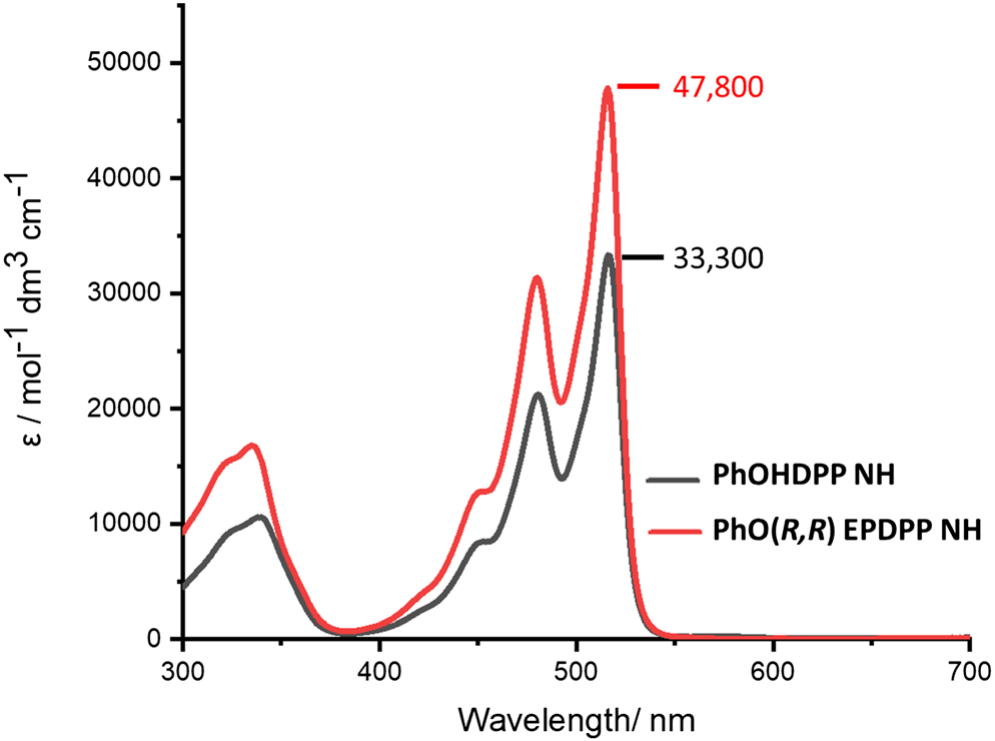
UV–visible absorption spectra of **PhOHDPP *N*H** and **PhO(*R,R*) EP DPP *N*H** in DMSO (2.2 × 10^−5^ Mol dm^−3^).

In solution, both **PhOHDPP *N*H** and **PhO(*R,R*) EP DPP *N*H** possess a two band absorbance profile typical of donor–acceptor systems of this type (Figure 1).^86^ The vibronic structure to the absorption band indicates the relative planarity, with little variation between the systems, indicating similar frontier orbital energies and band gaps.^87^, ^88^, ^89^, ^90^, ^91^ Functionalization with the ethyl propionate motifs leads to a near 50% improvement in ε in DMSO (Figure 1) compared to the phenol precursor, with the highest observed value of 54,100 dm^3^mol^−1^ cm^−1^ in THF (Supporting information S1: ESI 5.4), showing marked improvement versus **PhDPP *N*H** and similar unfunctionalized lactam DPP derivatives.^91^, ^92^ This property suggests good viability for light‐harvesting applications, giving comparative absorptivity to known highest absorbing **ThDPP** materials in the literature,^33^, ^93^ which have been successfully incorporated into optoelectronic devices.^94^

#### 2.2.2 Solution‐state emissive properties

**FIGURE 2:**
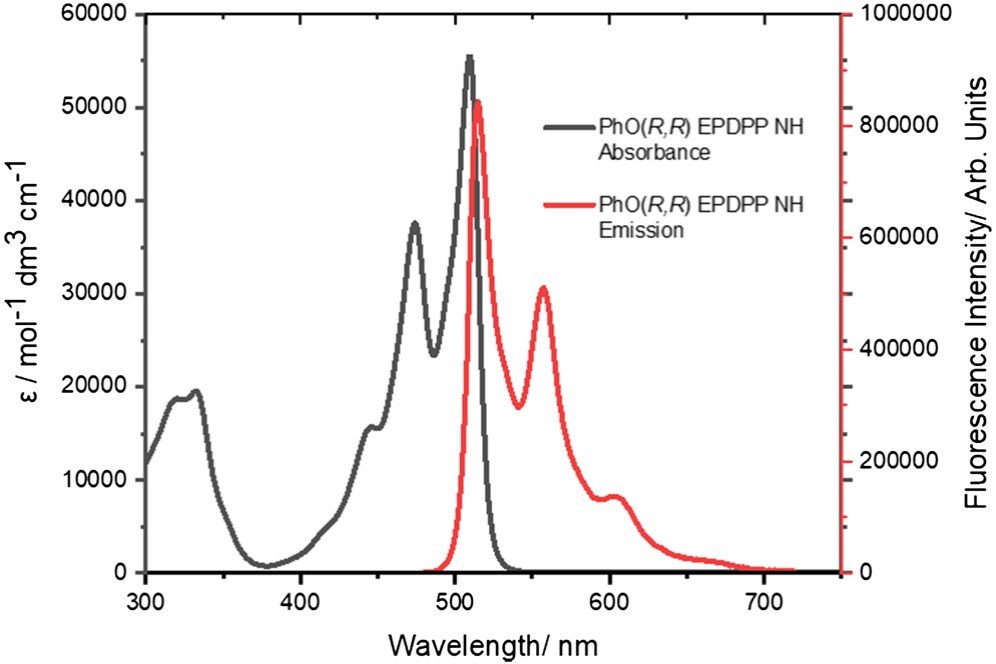
UV–visible absorption and emission spectra of **PhO(*R,R*) EP DPP *N*H** in THF. Excitation wavelength 470 nm.

As with absorption, **PhO(*R,R*) EP DPP *N*H** displays characteristic emissive behavior exhibited by other **PhDPP *N*H** analogs,^74^, ^90^, ^91^, ^95^ with mirror image absorption and emission profiles and retention of vibronic structure (Figure 2). This feature is indicative of a highly planar molecule in both the ground and the excited state, with a small difference in energy between the 0–0 vibronic transitions in absorption and emission, manifesting in a small Stokes shift (6 nm). The compound is highly emissive, indicated by the quantum yield (Supporting information S1: ESI 5.4), showing one of the highest values recorded for unfunctionalized lactam DPPs (89%).^33^, ^96^, ^97^ The emission is quenched in the solid state, presumably because of the proximity of the aromatic cores in this phase.^74^

#### 2.2.3 Solid‐state absorption properties

**FIGURE 3:**
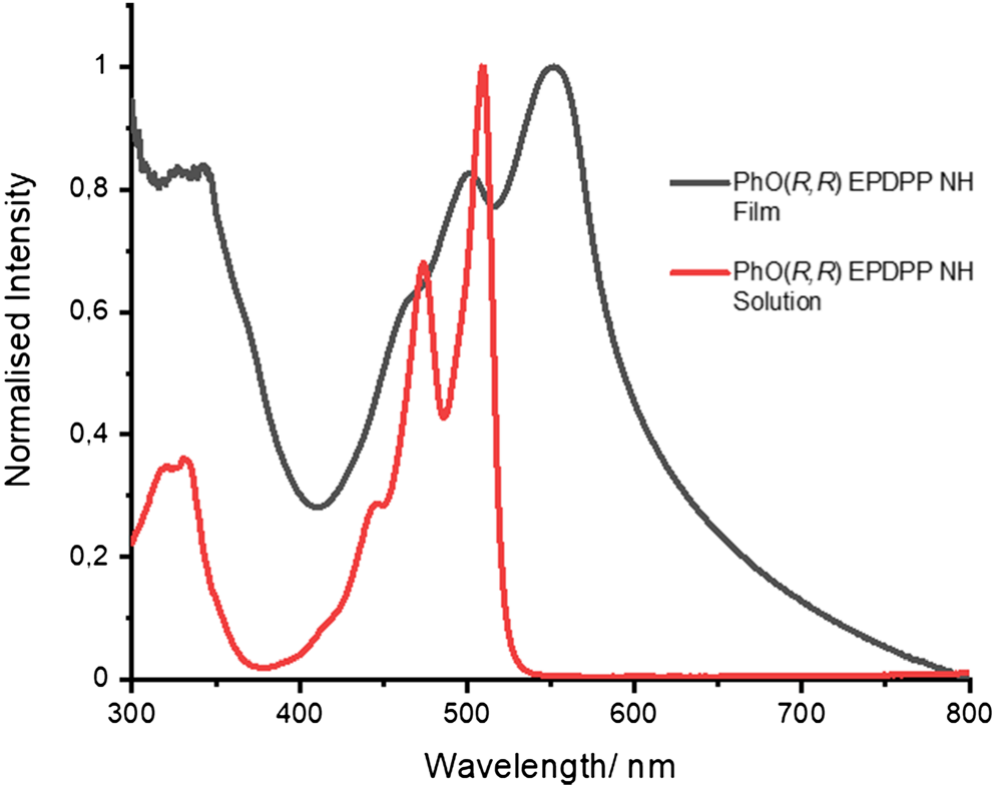
UV/Vis absorption spectra of **PhO(*R,R*) EP DPP *N*H** in solution in THF and a drop‐cast thin film from THF.

Solid‐state absorption spectroscopy is a useful tool to determine the arrangement of DPPs of this type in thin films.^74^ To prepare the samples, concentrated solutions of the compounds in THF (0.5 mg/ml) were drop‐cast onto glass, and the solid‐state absorption spectra were recorded. In both cases (Figure 3 and Supporting information S1: ESI 5.5), there is a bathochromic shift (42 nm) and a visible broadening of the film spectrum relative to the solution. These effects are attributed to close intermolecular interactions in the solid state, aided by hydrogen bonding that is observed by infrared (IR) spectroscopy (see below).^34^, ^98^ The bathochromic shift in the film indicates that the molecules stack in a head‐to‐tail orientation, suggesting *J*‐type aggregation. From the absorption edge of the film (602 nm), the band gap is calculated to be ~2.0 eV (Supporting information S1: ESI 5.6).

### 2.3 Redox properties

To assess donor–acceptor capability, frontier orbital energies were estimated from cyclic voltammetry measurements. The voltammograms demonstrate that **PhO(*R,R*) EP DPP *N*H** has an irreversible oxidation, shown by the absence of a return wave (Supporting information S1: ESI 5.7). Indeed, the voltammograms performed with different scan rates (Supporting information S1: ESI 5.8 and 5.9) suggest that the species generated either by oxidation or by reduction have poor stability under the conditions of the experiment. The oxidation appears to be a two‐electron process from the current magnitude, and we suggest that the dication system will form a quinoidal‐type structure similar to those observed for other DPP systems.^99^, ^100^, ^101^, ^102^ The two‐electron process indicates the system's electron richness and potential as an electron donor system. Previously, we observed that for the two‐electron oxidation of **PhOMeDPP *N*‐Hex** and **PhOHDPP *N*‐Hex**, the presence of an acidic hydrogen atom in the latter destabilizes the radical cation,^74^ which could also be the case here from the lactam hydrogen atom. The electron‐accepting ability is indicated by the single one‐electron quasi‐reversible reduction process (Supporting information S1: ESI 5.7–5.9).

Both **PhO(*R,R*) EP DPP *N*H** and its enantiomer (that displays identical voltammetric behavior) possess highest occupied molecular orbital (HOMO) and lowest unoccupied molecular orbital (LUMO) energy level values typical of a donor system, with an electrochemical band gap of 1.96 eV,^9^ giving good agreement with the optical band gap (Table 1) and a comparable band gap to other lactam‐free DPPs, namely, **PhDPP NH**, **PhClDPP NH**, and **PhBrDPP NH** (2.1 eV) (Supporting information S1: ESI 5.11), which all displayed good mobility values.^34^

**TABLE 1:** Experimental frontier orbital energy levels determined through cyclic voltammetry measurements in solution and optical band gap from thin film absorption edge for **PhO(*R,R*) EP DPP *N*H**.

Compound	Oxidation onset [V]	Reduction onset [V]	HOMO [eV]	LUMO [eV]	Electronic E_g_ [eV]	Optical E_g_ (film) [eV]	LUMO (film) [eV]
**PhO(*R,R*) EP DPP *N*H**	0.300^a^	−1.66^a^	−5.10^a^	−3.14^a^	1.96^a^	2.06^b^	−3.04^b^

^a^ Determined from cyclic voltammetry measurements in solution of compound (1 mmol), electrolyte (tetrabutylammonium hexafluorophosphate, 0.1 M) in DMF. Values referenced against the ferrocenium–ferrocene redox couple.

^b^ Thin films were formed on glass slides by drop casting of a concentrated THF solution (0.5 mg/ml).

### 2.4 Formation of twisted microtapes

**FIGURE 4:**
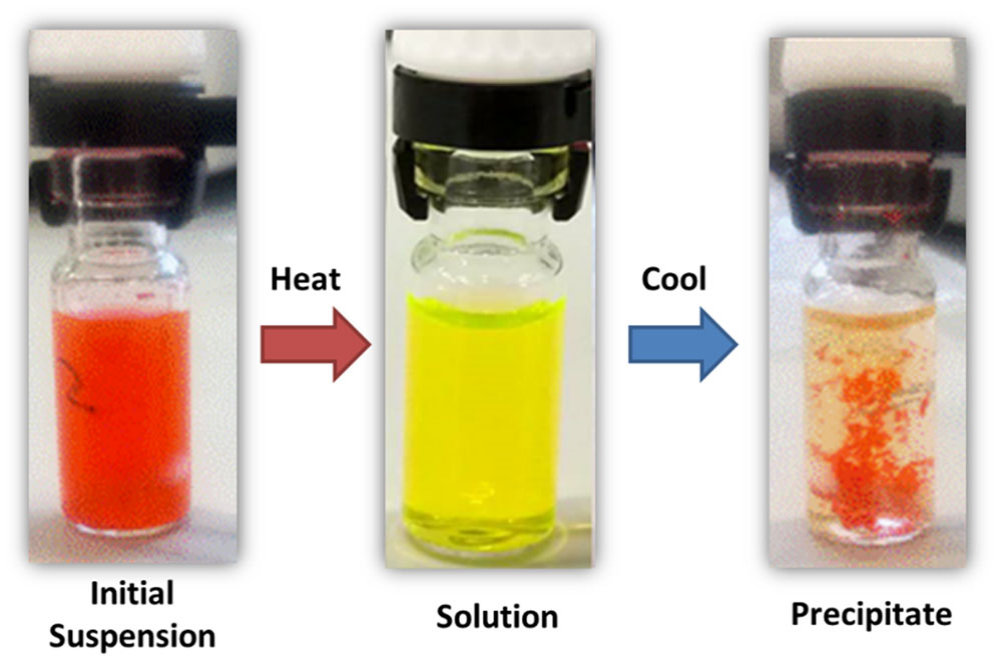
Photographs showing the appearance of an initial suspension of **PhO(*S,S*) EP DPP NH** after sonication at room temperature, the solution of the compound near the boiling point of the solvent, and the flocculant appearance of the suspension containing a precipitate of helical aggregates after slow cooling.

Whereas solubility of the compounds is excellent in certain solvents, controlled cooling and vapor diffusion techniques in solvents of medium polarity lead to intriguing aggregated species. Whereas it is largely insoluble at room temperature in acetone, acetonitrile, and methanol, warming leads to complete dissolution of the compounds to give yellow solutions, from red powders (Figure 4). Cooling leads to the formation of red flocculant‐type solids that were analyzed using different microscopes. The formation of the aggregates, kinetically determined in a similar way to crystal growth, is characteristic of other micron‐scale objects.^103^

**FIGURE 5:**
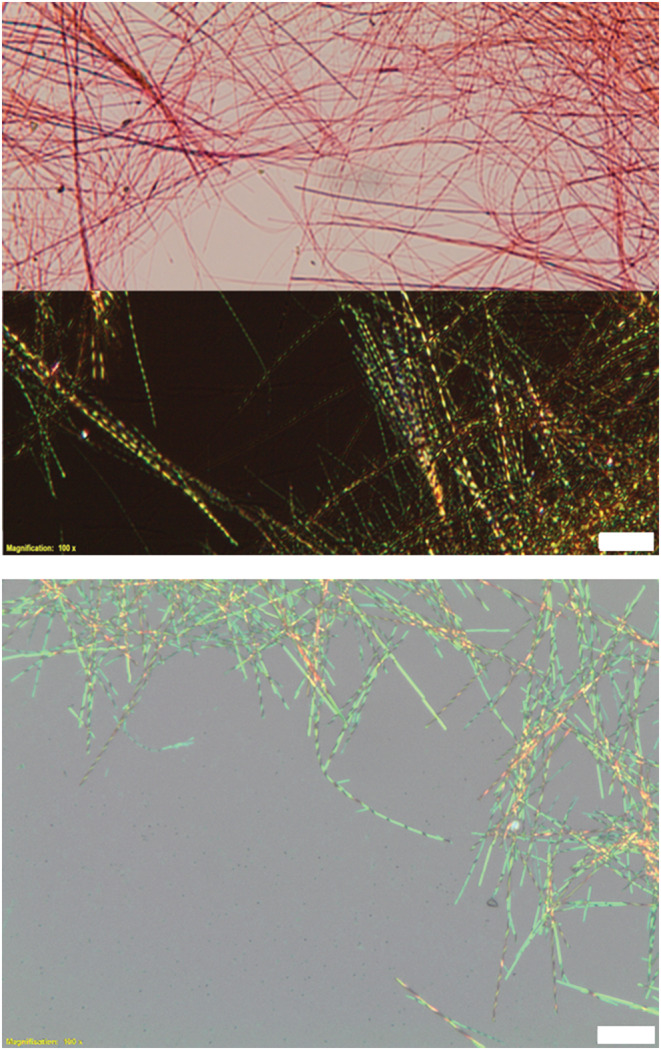
Optical microscope images (scale bars correspond to 5 μm) in transmission (top showing unpolarized and cross‐polarized images, the latter with the typical banded feature of helical objects) and reflectance mode of **PhO(*R*,*R*) EP DPP *N*H**, grown from acetonitrile (top) and acetone (below) solutions under variable temperature conditions showing the general morphology and twisting of the fibers.

Visualization of the solids formed by the individual enantiomers by polarizing optical microscopy showed that they comprised fibers with helical nature (Figure 5). Transmission micrographs show relatively featureless red fibers that are either straight (for the thicker objects) or curved, and all of them are dozens of microns in length, at least. Crossed polarizers inserted into the transmitted light beam show that the objects change regularly and periodically, giving banded features, indicative of regular twisting. The reflected light optical micrographs show the helical features most clearly.

Optimization of conditions to form these fibers was undertaken through solvent screening studies. We determined that ≈0.5 mg/ml solutions of either enantiomer in acetone, methanol, ethanol, or acetonitrile all lead to the formation of helical aggregates over time, after initial sonication to disperse in the media, followed by heating to solubilization and slow cooling. Minimum aggregation concentrations and optimal cooling rate were determined using a Crystal16 parallel crystallizer, which allowed greater precision to control heating and cooling rate, coupled with qualitative analysis through optical microscopy. Through these means, an optimal cooling rate of 1°C/min or slower after complete solubilization was seen to be crucial for the formation of well‐defined twisted structures.

Faster cooling led to much smaller aggregates, for which helicity could not be determined by optical methods. This observation suggests that the fibers are formed under kinetic control rather than thermodynamic.^104^ The minimum concentration for the formation of well‐defined helical structures was seen to be in the range 0.1–0.2 mg/ml, depending on solvent system (Supporting information S1: ESI 5.12). Initial growth was determined through a change in turbidity upon cooling of the solution, typically seen to be 5–10°C below the boiling point of the solvent (Supporting information S1: ESI 5.13). Additionally, vapor diffusion of *tert‐*butyl methyl ether into a 0.2 mg/ml THF solution of **PhO(*R,R*) EP DPP *N*H** or **PhO(*S,S*) EP DPP *N*H** also led to the formation of helical aggregates after several days (Supporting information S1: ESI 5.14), which could also be promising for post‐deposition solvent annealing.^105^, ^106^

Optical microscope images (Figure 5 and Supporting information S1: ESI 5.15–5.23) detail the well‐defined structures formed from variable temperature and vapor diffusion as mentioned previously. In reflectance mode (Supporting information S1: ESI 5.16–17), the opposite twist for each enantiomer is clearly seen, showing that molecular chirality is dictating the direction of the twist. From several images (Supporting information S1: ESI 5.24), it was seen that there are also quite a few fractured fibers resembling straight plates, a possible result of unwinding upon deposition on the surface. The helical fibers display birefringence at the node, but upon unraveling, the species no longer polarize the light, suggesting the materials have lost order. Seemingly thicker fibers form through bundling of smaller fibers together (Supporting information S1: ESI 5.22).

Twisted crystals of this nature have been observed in the formation of solids from a range of organic compounds, generally from the melt, and are surprisingly common.^107^, ^108^, ^109^ It is believed that over a quarter of simple molecular crystals can be grown with a twisted morphology from the melt,^110^, ^111^, ^112^ but those precipitated from solution are far rarer. Indeed, there are few examples where, like here, mesoscale twisting can be directly linked to the chirality of the crystallizing molecules.^113^, ^114^ More usually, the connection between molecular chirality and supramolecular chirality is enigmatic, and there is no direct correlation between molecular and supramolecular chirality.^115^ There are currently several accepted mechanisms for the formation of twisted crystals, all of which are united by the common theme of stress‐relief.^108^, ^109^ This stress can come from a variety of sources including dislocations as in the Eshelby mechanism; unbalanced surface stresses typically seen in polymers crystallized form the melt or heterometry stresses in the auto deformation mechanism.

Studies into the formation aggregates were also conducted with 50:50 mixtures of the two enantiomers through variable temperature and vapor diffusion, but no helicity was observed, and only straight, short plates were formed (Supporting information S1: ESI 5.25). This result indicates that the racemic compound is formed preferentially. It also indicates that the enantiomers have a high degree of optical purity, as the twisted objects seen by microscopy are very morphologically pure, enantiomeric impurities would be expected to lead to microscopic heterogeneity in the aggregates. Attempts to grow single crystals of **PhO(*R,R*) EP DPP *N*H, PhO(*S,S*) EP DPP *N*H**, or their mixture, through various techniques failed to produce any objects of sufficient size and quality for single crystal diffraction studies.

**FIGURE 6:**
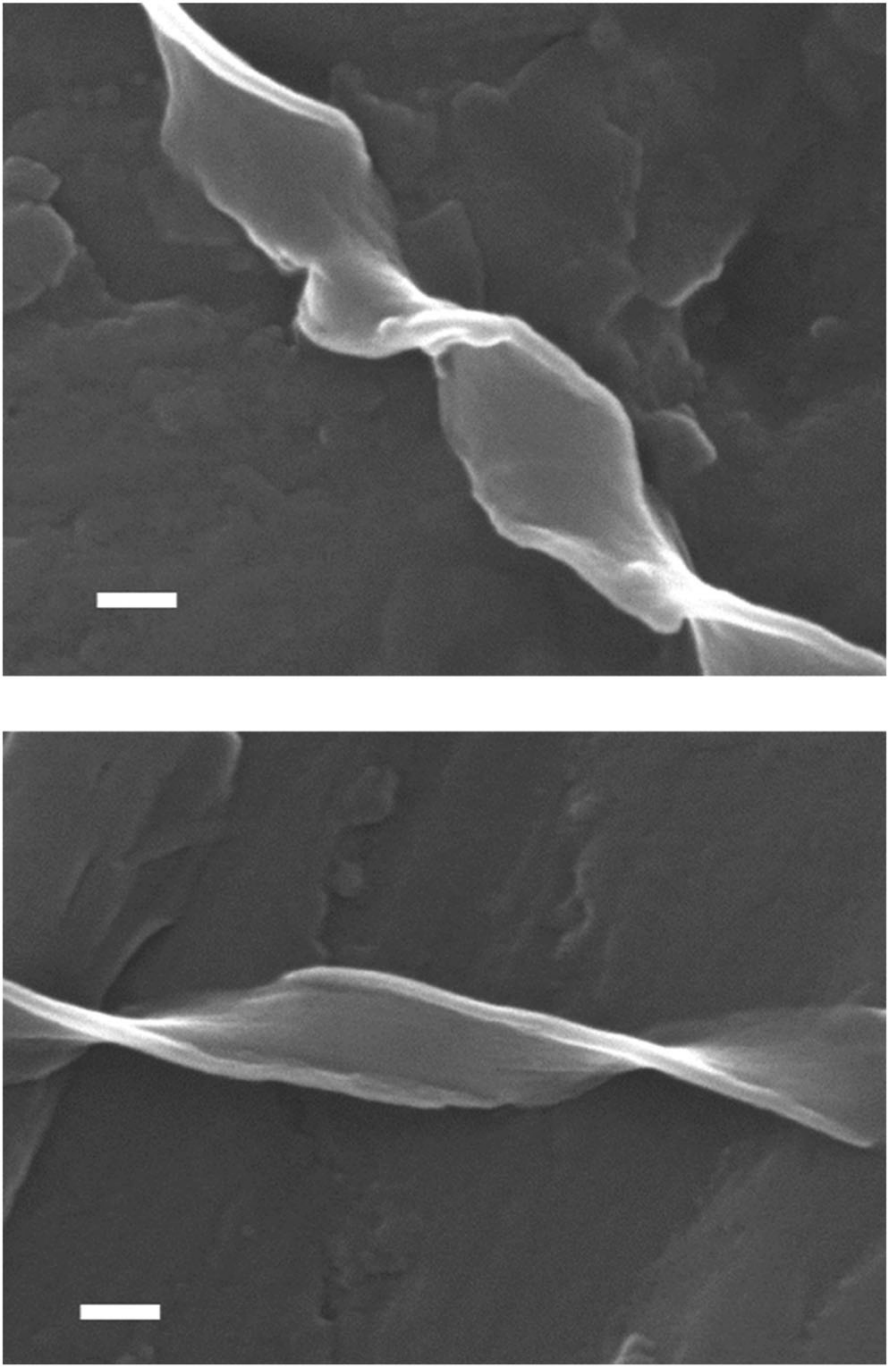
Scanning electron microscopy (SEM) image of **PhO(*S,S*) EP DPP *N*H** (top) and **PhO(*R,R*) EP DPP *N*H** (bottom), grown from acetone solutions under variable temperature conditions showing the general morphology and twisting of the fibers that are opposite for the two enantiomers. The scale bar corresponds to 100 nm.

**FIGURE 7:**
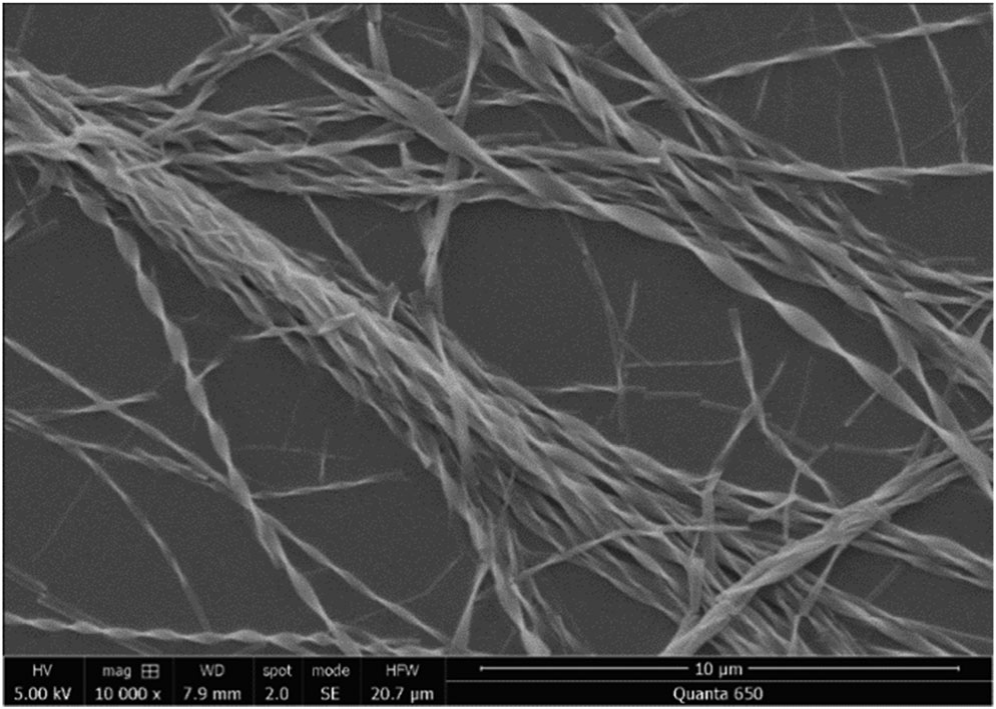
Scanning electron microscopy (SEM) image of **PhO(*R,R*) EP DPP *N*H** grown from an acetonitrile solution under variable temperature conditions showing the general morphology and twisting of the fibers.

Given the promising optical microscope images, the chiral aggregates were further characterized using scanning electron microscopy (SEM). Figure 6 and Supporting information S1: ESI 5.26–5.45 show examples of micron‐scale twisted tapes grown from acetone, acetonitrile, methanol, and vapor diffusion of *tert*‐butyl methyl ether into THF for **PhO(*R*,*R*) EP DPP *N*H** and **PhO(*S*,*S*) EP DPP *N*H**, showing the previously mentioned opposing twists, dictated by point chirality of the molecule. The aforementioned bundling of fibers to make larger structures is seen most prominently in the acetonitrile grown fibers (Figure 7). As observed with optical microscopy in some cases, deposition onto the SEM stub leads to unraveling of fibers (Supporting information S1: ESI 5.45), and the relative thinness can be seen by the fact fibers, which are visible underneath.

There is generally a well‐defined relationship between the pitch of the observed twisting and the dimensions of the crystalline objects as it has been shown that twisted crystals unwind as they grow.^111^ Most systems exhibit a correlation of the pitch (P), and h is the smallest cross‐sectional size, for example, the thickness of a ribbon ^108^, ^109^ with the exception of those grown via the Eshelby twist, which can be fitted to the function P ~h^2^.^116^ A twist‐pitch analysis of fibers was therefore performed for both **PhO(*R,R*) EP DPP *N*H** and **PhO(*S,S*) EP DPP *N*H** structures formed from an acetone solution, and data can be fitted to the equation *P* = kh^n^, where *n* is equal to 0.47 and 0.49, respectively, ruling out the Eshelby twist as their growth mechanism (Supporting information S1: ESI 5.46–5.48). Both data sets lie below the line 2P = 103πh, and therefore, auto deformation and surface stresses can be at least partially relieved through twisting.

IR measurements of the evaporated films of the chiral DPP derivatives from the solution in THF, suspended aggregates in acetone, acetonitrile, and THF vapor diffused with *tert*‐butyl methyl ether, showed identical spectra for the NH region, displaying only a signal corresponding to a hydrogen‐bonded NH group,^44^, ^59^, ^72^, ^117^ and no free amide NH, with similar findings for the carbonyl (Supporting information S1: ESI 5.49–5.50), showing that hydrogen bonding is driving aggregation.

### 2.5 CD spectroscopy

CD measurements were undertaken in solution to deduce the optical activity of the enantiomeric DPP derivatives. Even at very high concentrations in THF, only weak optical activity is observed (see Supporting information S1: ESI 5.51–5.52), with no apparent concentration dependence, indicating that the CD signal arises as a result of the totally solvated monomeric species. Two main peaks are observed, one at low wavelength, 262 nm, and one at higher wavelength, 546 nm. There is little coupling between the stereogenic center and the main chromophores in the molecules in this kind of unit, where the peak at around 260 nm is also observed, corresponding to the phenyl‐O‐lactate unit.^118^, ^119^

Initial screening of media showed that substantial optical activity is observed in the solvent systems previously described to form chiral fibers, namely, acetone, acetonitrile, and ethanol. Solutions containing aggregates were prepared through heating to solubilization and slow cooling at ≈1°C/min. CD spectra in acetone showed opposing signs for each enantiomer, with a positive Cotton effect at ≈565 nm (∆ε 3.4) for **PhO** **(** ***R**,**R*** **) EP** **DPP** ***N*** **H** and a second positive Cotton effect at 352 nm (∆ε 2.2). Upon heating, loss of optical activity and the *J*‐aggregate band at ≈550 nm in absorption (Supporting information S1: ESI 5.53–5.57) ensues, owing to dissolution of the aggregate and return to the monomeric form. Relatively fast cooling (2.5°C/min) fails to regenerate the signal of the aggregate confirming previous finding that a cooling rate of 1°C/min or lower is required to form helical structures (Supporting information S1: ESI 5.55). Owing to the UV cut off of acetone and its volatility, ethanol was chosen for further study. The solution was first heated to 75°C, and the CD spectra were recorded, but the compounds are not fully solubilized at this concentration, and so complicated multisignate spectra are observed (Supporting information S1: ESI 5.58–5.59). Both enantiomers exhibit a bisignate exciton couplet that has a center at 275 nm, which can be attributed to the coupling between the π–π* transition dipole moments. **PhO** **(** ***R**,**R*** **) EP DPP** ***N*** **H** and **PhO(** ***S**,**S*** **) EP DPP** ***N*** **H** exhibit negative and positive exciton couplets, respectively, at this wavelength.

**FIGURE 8:**
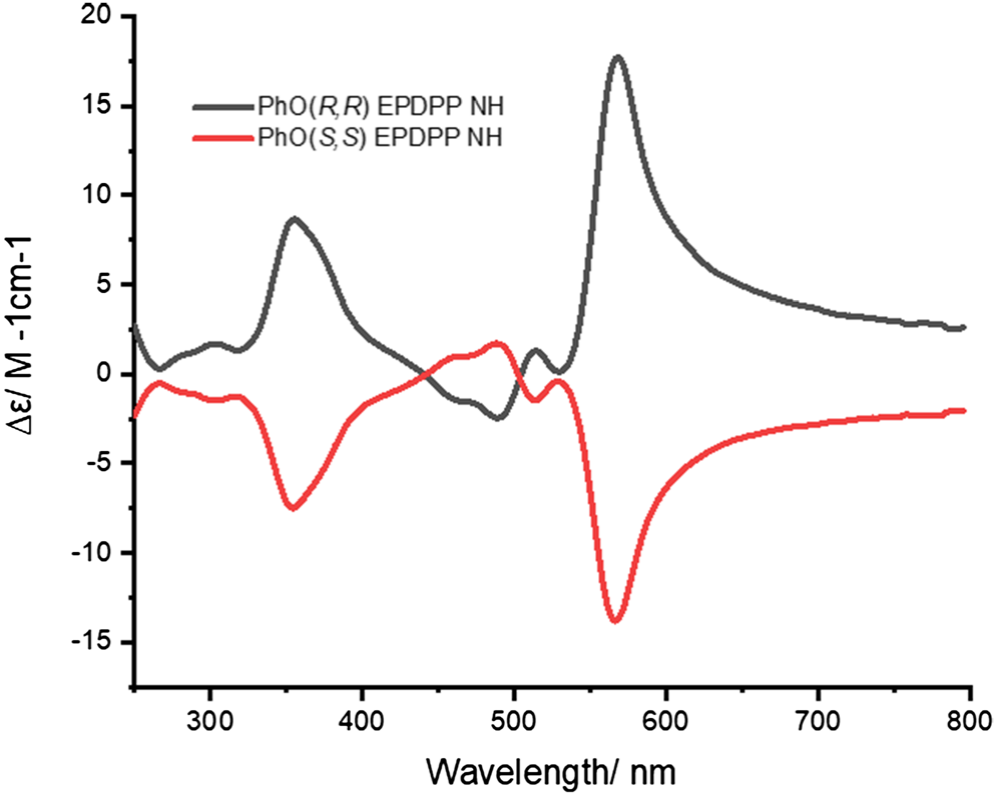
Circular dichroism spectra of **PhO(** ***R,R*** **) EP DPP** ***N*** **H** (black) and **PhO(** ***S,S*** **) EP DPP** ***N*** **H** (red) in 0.35 mmol solutions of ethanol at 5°C.

The restriction of flexibility that occurs upon aggregation of these molecules into chiral macrostructures could be the reason that complicated multisignate Cotton effects are observed at the low‐energy end of the spectrum.^120^ At this temperature, CD relative to the *J*‐aggregate is minor compared with higher energy bands. The solution was cooled to 5°C at a rate of 1°C per min and aggregation ensued. As was the case in acetone, CD spectra at this temperature exhibit two large Cotton effects, which are an order of magnitude greater than seen at the lower temperature, with maxima at 355 and 567 nm, both of which are positive for **PhO(** ***R,R*** **) EP DPP** ***N*** **H** and negative for **PhO** **(** ***S,S*** **) EP DPP** ***N*** **H** (Figure 8), accompanied by a *J*‐aggregate band at ≈550 nm observed in the absorption spectra^59^ (Supporting information S1: ESI 5.60). There is little CD signal at the wavelength of the main absorption band in both solvent systems. A considerable increase in ∆ε is observed for both signals in ethanol when compared with acetone (∆ε ≈ ±15, ∆ε ≈ ±7.5 vs. ∆ε ≈ ±3.4, ∆ε ≈ ± 2.2).

### 2.6 Circularly polarized luminescence

**FIGURE 9:**
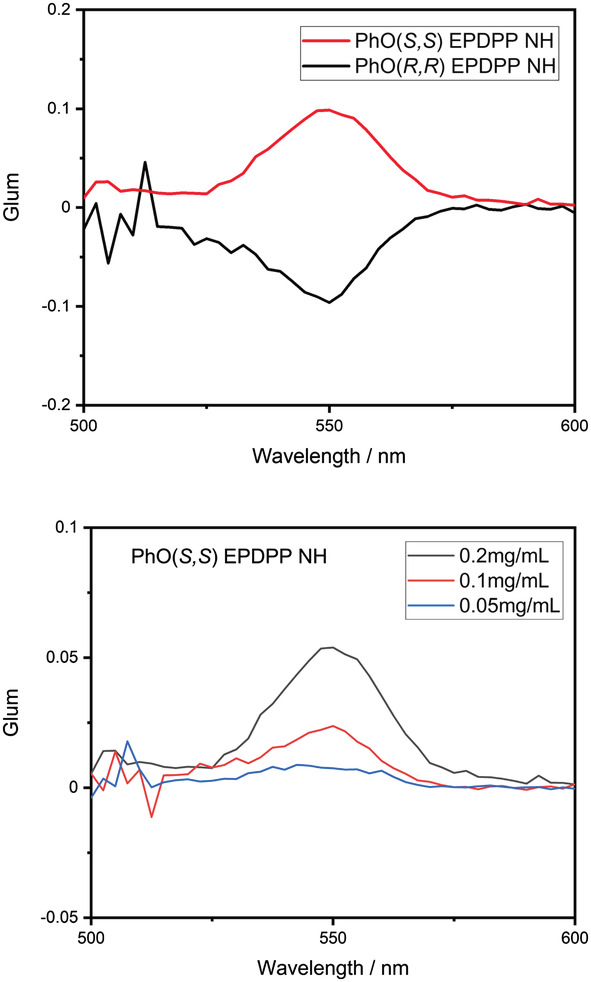
Circularly polarized luminescence (CPL) spectra of **PhO(*R,R*) EP DPP *N*H** (red) and **PhO(*S,S*) EP DPP *N*H** (black) in 0.35 mmol solutions in ethanol (top) and CPL spectra of **PhO(*S,S*) EP DPP *N*H** in acetone at different concentrations.

The strong CD observed and the excellent emissive properties of the system prompted us to investigate the CPL of these derivatives in ethanol. Both enantiomers exhibit circularly polarized luminescence, with peaks at 550 nm corresponding to the 1–0 vibronic transition of the fluorescence spectra (Figure 9 and Supporting information S1: ESI 5.61) and equivalent to the wavelength of the *J*‐aggregate in absorption. **PhO(** ***S,S*** **) EP DPP** ***N*** **H** displays a positive CPL signal, whereas **PhO(** ***R,R*** **) EP DPP** ***N*** **H** displays a negative one, opposite to CD. Literature studies of β‐γ‐enones attribute this unusual attribute to a reversal of local chirality between the ground and the excited state, for which additional interactions of the excited state are responsible.^121^, ^122^ G_lum_ values of ≈ ± 0.1 show marked improvement compared to previously reported DPP systems.^66^, ^68^ This high asymmetry in CPL is a result of aggregation, as the solution in THF shows no detectable CPL signal. In addition, recording the CPL for the (*S,S*) enantiomer in acetone at different concentrations revealed a dependence of the intensity on this factor (Figure 9). These data therefore support an aggregation‐induced CPL mechanism, similar to that seen previously,^123^, ^124^ and whose origin must lie in the asymmetry induced in the chromophores upon their self‐assembly, in a conceptually similar manner to increased CD on aggregation.

### 2.7 Solid‐state CD

Given the promising chiroptical absorption properties in solution and the ambition to produce materials for organic electronics, thin films were deposited by drop casting a 2 mmol THF solution of **PhO(*R,R*) EP DPP *N*H** onto a quartz disk to study CD in the solid state (Supporting information S1: ESI 5.62). We considered that it would be interesting to use a mapping technique to study the apparent CD spectra over an area of the sample.^125^, ^126^ Therefore, the CD spectra were recorded in imaging mode using the Module B spectrophotometer at B23 beamline for synchrotron Radiation CD (SRCS) of Diamond Light Source,^127^ which can be used to map optical activity in solid films^128^, ^129^ with a resolution of approximately 50 μm. Therefore, the optical activity of any microscopic objects cannot be resolved, but the overall CD signal can be mapped in the regions of interest.

**FIGURE 10:**
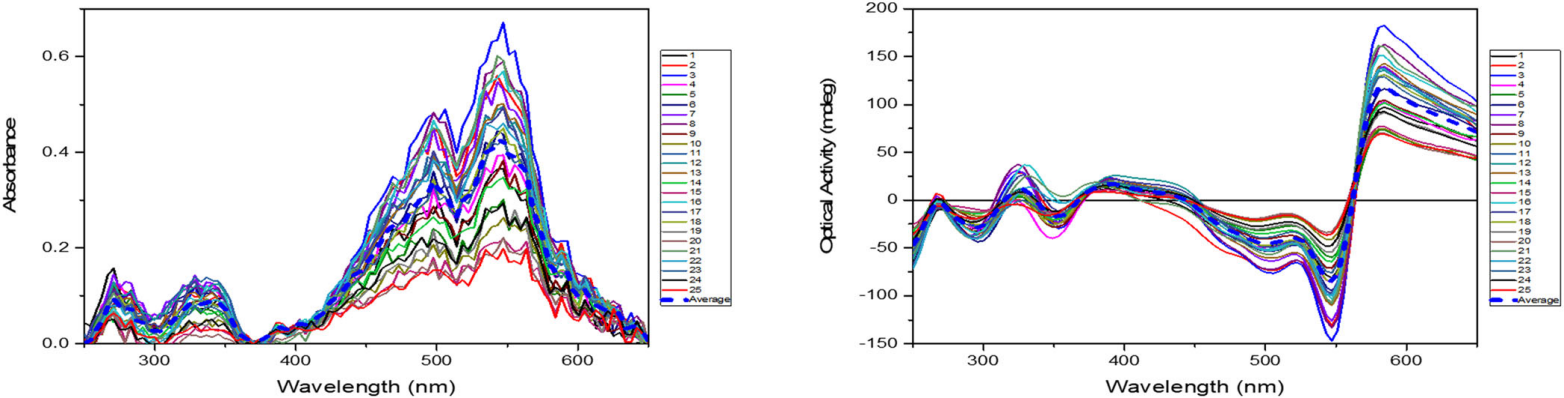
Circular dichroism spectra of 25 scans (step size of 50 μm) and average (blue dotted line) of a drop cast film from a 2.00 mmol solution of **PhO(*R,R*) EP DPP *N*H** in THF.

**FIGURE 11:**
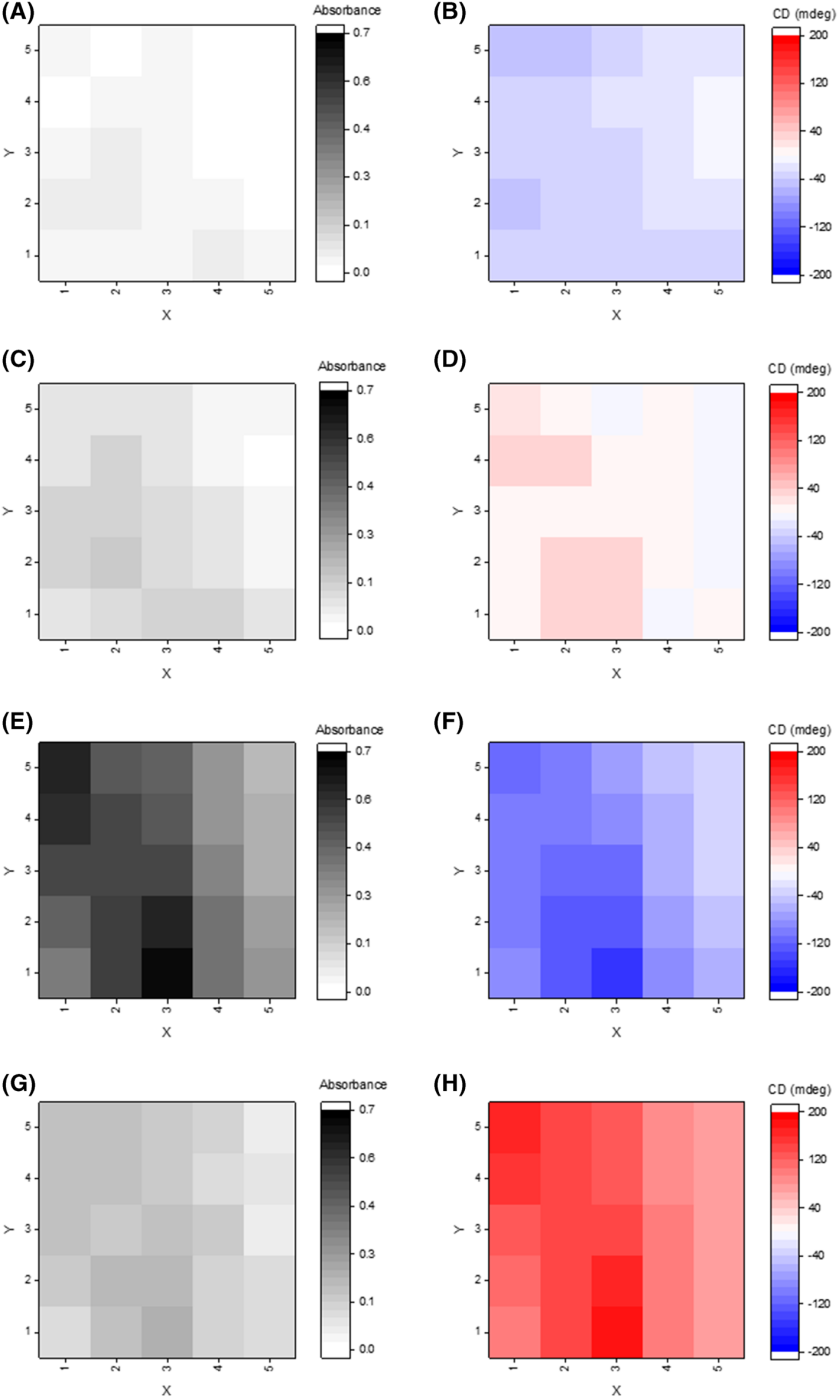
2D optical activity maps of a cast film of **PhO(*R,R*) EP DPP *N*H** in THF. Absorbance versus X‐Y (gray hues) and CD intensity versus X‐Y (red and blue hues) for each of the wavelengths that had the most intense optical activity 294 nm (A and B), 326 nm (C and D), 546 nm (E and F), and 582 nm (G and H); each square represents a 50 × 50 μm area where the center was illuminated.

As seen (Figure 10), the film gives rise to two positive Cotton effects at 308 and 562 nm at room temperature, with the latter having a significantly greater intensity. The Cotton effect at 562 nm coincides with the solid‐state absorption maxima (Figure 3, Supporting information S1: ESI 5.63). Compared with the solution‐state CD spectra, the main difference is in the 500–600 nm region where in solution there is a positive Cotton effect and in the solid state a positive excitonic couplet. The wavelength at which the strongest optical activities were observed was plotted as 2D color maps to look at the distribution of the film (Figure 11), which show homogeneous distribution of the sign of the optical activity at any given wavelength, whereas the magnitude varies in a way that does not match exactly the absorbance of the films. These imaging CD experiments therefore show a heterogeneity in the supramolecular arrangement of the molecules in the film.

The inconsistencies in intensity between absorbance and optical activity are most evident at 294 nm (maps A and B), where the strongest absorbance is observed in squares (1,2), (2,2), (2,3), and (4,1), whereas the most intense optical activity is observed in squares (1,2), (1,5), and (2,5), suggesting contributions from linear dichroism and/or birefringence. For 326 nm (Map D), both positive and negative signals are observed. Rotation of the sample by 180° displays a change in intensity of the Cotton effect at 562 nm, further evidence of the aforementioned additional factors (Supporting information S1: ESI 5.64). These are likely a product of the deposition method, given the previously observed sensitivity of thin film formation technique on the supramolecular ordering and homogeneity.^130^

### 2.8 Monolayer formation

**FIGURE 12:**
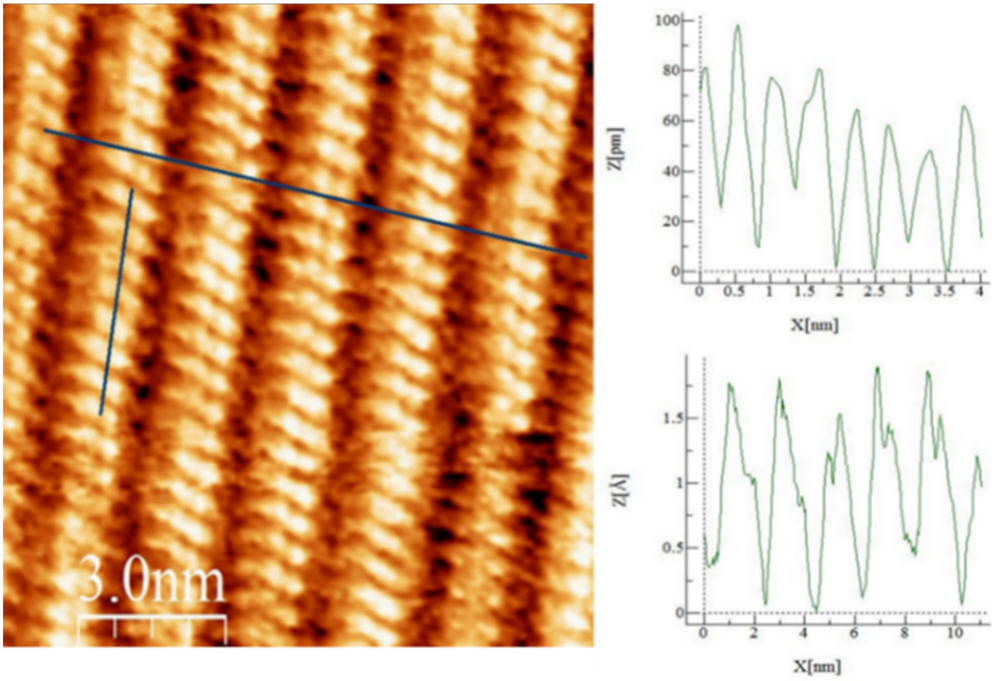
Self‐assembled monolayer of **PhO(*S,S*) EP DPP *N*H** formed from 10^−5^ mol dm^−3^ THF solution on HOPG imaging in a phenyl octane droplet (V_bias_ = −700 mV I_set_ = 300 pA). Inset profiles showing lamella width and length.

Scanning tunneling microscopy (STM) has been utilized to study the self‐assembly of DPP derivatives.^76^, ^131^ Hence, STM studies were undertaken to see how the **PhO(*S,S*) EP DPP *N*H** molecules organized on a highly oriented pyrolytic graphite (HOPG) surface. The monolayer was grown from immersion of HOPG in a 10^−5^ mol dm^−3^ THF solution and imaging in a drop of phenyl octane. **PhO**(*S,S*) **EP DPP *N*H** formed well‐defined lamellae of the nature seen in Figure 12 and Supporting information S1: ESI 5.65, displaying good uniformity, with defects in packing being rare (Supporting information S1: ESI 5.66). Lamellar dimensions were equivalent to that of a single molecule of **PhO(*S,S*) EP DPP *N*H** and stacked in a slipped stacked manner (Figure 12 and Supporting information S1: ESI 5.67), likely driven by the hydrogen bonding and close packing.^76^ The dynamic nature of the self‐assembled structure is evidenced by the formation and disappearance of a hole upon continual scanning (Supporting information S1: ESI 5.68). The tilt of the cores with respect to the lamellar axis is unique for each enantiomer and shows how the established transfer of chirality from stereogenic centers to monolayer structure^132^ can also be applied in these molecules.

## 3 CONCLUSION

We have synthesized the novel pair of π‐functional enantiomeric chiral chromophores in good yield (53%–56%). The compounds display excellent photophysical properties for light‐harvesting (ε = 54,100 dm^3^mol^−1^ cm^−1^) and emissive (Φ = 0.89) applications in solution. Electronically, the materials behave as donor‐type systems with a two‐electron irreversible oxidation and single electron quasi‐reversible reduction, with hydrogen bonding inhibiting the stabilization of the radical cation species formed in the redox process. Thin film absorption spectra, from drop‐cast THF solutions, show a broadening and bathochromic shift compared with solution spectra, owing to the intermolecular hydrogen bonding and π–π stacking in the solid state. Optical band gaps of 2.0 eV give good agreement with cyclic voltammetry measurements. STM measurements show that for monolayers formed from immersion of HOPG in THF solutions, molecules arrange into 1D lamellae. Monomeric CD spectra are observed in THF, with no perceived aggregation, whereas in the solid‐state enhancement of CD is observed with a positive excitonic couplet at 562 nm. In solvents that promote aggregation, CD spectra display Cotton effects at ≈567 and at ≈355 nm, both of the same sign, depending on the enantiomer. In ethanol, the molecules aggregate giving a significant increase in ∆ε when compared with acetone (∆ε ≈ ±15 vs. ∆ε ≈ ±3.4). Heating to dissolution leads to loss of the *J*‐aggregate band in absorption and diminished CD signal, which fails to return at a cooling rate of 2.5°C/min, suggesting that the structures are former under kinetic rather than thermodynamic control. Owing to the emissive nature of the compounds, CPL is also observed. In said solvent systems that promote aggregation at concentrations of 0.1–0.2 mg/ml, after heating to solubilization and cooling at 1°C/min, helical fibers are observed, with the twist seemingly dictated by the molecular chirality at the stereogenic center. Self‐assembly of the helical fibers is likely driven by the interplay between intermolecular hydrogen bonding and π–π stacking,^133^ with hydrogen‐bonding driving assembly of these structures and chirality dictating the twisting direction.^134^ Literature precedent shows that often similar systems tend to form hydrogen‐bonded oligomers, which assemble into disordered stacks that ultimately lead to the formation of a chiral helix.^133^, ^135^, ^136^, ^137^, ^138^, ^139^, ^140^ From the evidence displayed, it seems that in THF the system, it self‐assembles to form lamellar structures. In solvent systems that promote aggregation, helical structures are formed, as well as through vapor diffusion into THF solutions. In such solvents that promote the formation of helical aggregates, when a racemic mixture is utilized, straight plate‐type structures are produced, with no twisting. When helical structures are deposited on a surface of quartz or mica, unraveling of the fiber at the surface can occur, forming plate‐type structures, similar to observations for the racemic mixture and akin to the lamellae from STM. This suggests the fact that oligomers are formed as straight plate lamellar assemblies and then a defect promotes the twist, be it through solvent interaction^133^ or another means, and the helicity is dictated by molecular chirality. From an optoelectronic device point of view, solvent dictation of morphology is particularly interesting especially in the case of potential vapor annealing to change morphology from a lamellae structure to a helical system and the subsequent influence on performance. Future design of materials could look to increase conjugation while retaining the favorable optoelectronic and self‐assembly properties of the subjects of this article or their use as a morphological additive through a sergeants and soldiers' chiral induction approach^141^ to dictate morphology of state‐of‐the‐art donors. Ultimately, this may lead to organic semiconductors with long charge carrier lifetimes.^142^ The DPPs are particularly promising in this regard.^143^ In a broader sense, they could also lead to new properties arising from the molecular chirality and charge transfer properties,^144^, ^145^ including those displayed by twisted crystals.^146^, ^147^

## Supporting information


**Data S1.** Supporting Information.

## Data Availability

The data that support the findings of this study are available in the Supporting information S1 of this article, which can be found in the online version of this article at the publisher's website.
