# Supplementary material for: Self‐assembly of chiral diketopyrrolopyrrole chromophores giving supramolecular chains in monolayers and twisted microtapes
Source: Chirality. 2023 Feb 9;35(5):281–97. doi: 10.1002/chir.23539 (PMC10947275; doi:10.1002/chir.23539)

Electronic supplementary information (ESI) for:

Self-assembly of chiral diketopyrrolopyrrole chromophores to give supramolecular twisted microtapes

Joshua Humphreys,[a,b] C. Elizabeth Killalea, [a,b] Flavia Pop, [a,b,§ ¶] E. Stephen Davies,[b] Giuliano Siligardi[c] and David B. Amabilino*[a,b,c]

a The GSK Carbon Neutral Laboratories for Sustainable Chemistry, The University of Nottingham Jubilee Campus, Triumph Road, Nottingham NG7 2TU, UK.

b School of Chemistry, University of Nottingham, Nottingham NG7 2RD, UK.

c Diamond Light Source, Harwell Science and Innovation Campus, Didcot, Oxfordshire OX11 0DE, UK

d Institut de Ciència de Materials de Barcelona (ICMAB-CSIC), Campus Universitari de Cerdanyola, 08193 Spain

§ Current Address MOLTECH-Anjou, UMR 6200, CNRS, Univ. Angers, 2bd Lavoisier, 49045 Angers, France

Contents

1. Experimental Procedures
2. ^{1}H NMR Spectra
3. ^{13}C NMR Spectra
4. IR Spectra
5. Supporting Figures

1. Experimental

1.1 Materials and instrumentation

All commercially available reagents were purchased from Sigma-Aldrich and used as received. Anhydrous solvents were fitted with an Aldrich Sure-Seal from purchase and used as received. Chromatography purifications were performed using Sigma-Aldrich Silica Gel (pore size 60Å, particle size 40- 63 μm) and thin-layer chromatography (TLC) was carried out on E. Merck silica gel plates, irradiated using UV light (365 nm). NMR spectra were acquired on a Bruker AV400, Bruker AV(III)500 or Bruker DPX300 spectrometers and NMR spectra were recorded at room temperature. All chemical shifts are reported in δ parts per million (ppm), using the solvent residual signal as an internal standard and the coupling constant values (*J*) are reported in Hertz (Hz). The following abbreviations are used for signal multiplicities: s, singlet; d, doublet; t, triplet; m, multiplet; and b, broad. ESI-MS were recorded using a Bruker micro-TOF II instrument. Infra-red spectra were recorded on a Bruker Tensor 27 instrument equipped with a Pike GladiATR attachment with a diamond crystal. Melting points were determined on a Stuart SMP20 Melting Point Apparatus. UV/Vis absorption studies were performed using a Cary 5000 UV-Vis-NIR Absorption spectrometer. Emission studies were performed using an Edinburgh Instruments FLS980 Photoluminescence spectrometer. For quantum yield studies, fluorescence spectra were recorded as aerated solutions using a Jobin Yvon Horiba FluoroMax-3 spectrometer at ambient temperature in a 1cm pathlength quartz cuvette. Quantum yields were calculated by comparison with the fluorescence observed for fluorescein (F = 91 in NaOH) under identical conditions of irradiation.[72] Circularly polarised luminescence (CPL) measurements were performed using a Circularly Polarised Luminsecence Accessory for the FLS980 Spectrometer which employs a photo-elastic modulator and lock-in amplifier counting box.

Cyclic voltammetric studies were carried out using an Autolab PGSTAT20 potentiostat. Standard cyclic voltammetry was carried out under an atmosphere of argon using a three-electrode arrangement in a single compartment cell. A glassy carbon working electrode, a Pt wire secondary electrode and a saturated calomel reference electrode, chemically isolated from the test solution *via* a bridge tube containing electrolyte solution and fitted with a porous Vycor frit, were used in the cell. Redox potentials are quoted versus the ferrocenium-ferrocene couple, which was used as an internal reference.[73] DMF was utilised as solvent and tetrabutylammonium hexafluorphosphae (0.1 M) was employed as supporting electrolyte for all electrochemical experiments. HOMO and LUMO levels were calculated using the equations:[74]

$E_{HOMO} = -(E_{onset}(ox) + 4.8\ [eV])$ and $E_{LUMO} = -(E_{onset}(red) + 4.8\ [eV])$.

The band gap is calculated using the equation

$E_g = E_{HOMO} - E_{LUMO}\ [eV]$.

Alternatively, the band gap can also be calculated from the onset of the optical absorption of the film using the equation: $E_g = 1240 / \lambda_{onset}$ [eV].

Circular dichroism spectra of solutions and aggregates were recorded on an Applied Photophysics Chirascan plus spectrometer while Circular Dichroism Imaging (CDi*)* experiments of thin films were recorded using Module B spectrophotometer at B23 beamline for Synchrotron Radiation CD (SRCS) of Diamond Light Source. Module B employs an Olis DSM20 Monochromator and Photomultiplier tube detector. The beam light was positioned in a vertical measurement chamber using a motorized XY stage and temperature variation was achieved with a Linkam hot stage. UV and CD spectra were recorded simultaneously in the range of 250 to 650 nm with 100 increments (which is equal to 4nm steps) over this range. UV*i* and CD*i* spectra were recorded by rastering 5 x 5 grids arrays with a step size 50 using a beam diameter of 50 µm resulting in 25 separate scans.

To determine solvent systems that promote the growth of the helical structures, various solvents were screened. Initially, a small amount of sample was added to a given solvent to form solutions of approximately 0.5 mg/ml in concentration. Sonication of the sealed vial was then undertaken to break any large particles that would prevent full solubilisation and once sufficiently broken, heating to solubilisation and cooling of the compound in acetone, methanol, acetonitrile (and structural variants) led to the formation of helical aggregates over time Variable temperature aggregation studies were performed using a Crystal16 parallel crystallizer. From experiments that consisted of heating and holding at the temperature of solubilisation (indicated by 100% transmission) followed by cooling, the minimum concentration for the formation of well-defined helical structures visible *via* optical microscope (determined qualitatively) was established. Slow cooling of the solution at 1 °C per hour was utilised to determine the growth initiation point where a change in transmissivity/ turbidity indicated the beginning of fibre formation.

Optical microscope images were obtained using a BX51-P Olympus polarising microscope with hot stage Scanning electron microscope images were obtained using either FEI Quanta 650, ESEM or JEOL 7100F FEG-SEM on mica. Scanning tunnelling microscope experiments were carried out on an Agilent Technologies 4500 PicoPlus STM using a PicoScan controller on HOPG. STM tips were formed from mechanically cut 80:20 PtIr wire. STM samples were prepeared through immersing a section of HOPG in a 10^{-5} mol dm^{-3} THF solution of the molecule of interest for approximately 20 seconds, allowing the formation of a self-assembled mono layer. The surface was then studied by adding a drop of phenyl octane.

1.2. Synthesis of compounds

4-(Tetrahydro-2-pyran-2H-yloxy)benzonitrile was synthesised according to previously published literature procedures with ^{1}H NMR in agreement with the target structure.[1] [2]

3,6-Bis(4-hydroxyphenyl)-2,5-dihydropyrrolo[3,4-c]pyrrole-1,4-dione (PhOHDPP *N*H)

Sodium (3.92 g, 170 mmol) and a catalytic amount of iron chloride were added to anhydrous *t*-amyl alcohol (112 ml, 1.02 mol) and heated at 120 °C for 1 hour, under an argon atmosphere, whilst stirring until all the sodium had dissolved. The mixture was then cooled to around 80 C and 4-(tetrahydro-2-pyran-2H-yloxy) benzonitrile (17.3 g, 84.6 mmol) added. The reaction was then heated slowly to 130 °C and once it had reached this temperature di-*tert* amyl succinate (10.5 ml, 47.9 mmol) in *t*-amyl alcohol (28.0 ml) was added *via* syringe pump over 3 hours. The reaction was then allowed to heat at 130 °C overnight. Upon completion, the reaction mixture was cooled, and hot methanol added. The reaction mixture was then filtered and the solid was stirred in 6M HCl (100 ml) in order to deprotect the pyran to give the free phenol. The mixture was then filtered over a glass sintered crucible (No. 4 porosity) and washed with hot methanol and allowed to dry in a vacuum desiccator to afford the pure product as a red solid (11.9 g , 43.7 %) Mp: >300 °C; ^{1}H NMR (400 MHz, DMSO d_6) δ 11.02 (bs, 2H), 10.38 (s, 2H), 8.35 (d, *J* = 8.5 Hz, 4H), 6.91 (d, *J* = 8.5 Hz, 4H) ppm; ^{13}C NMR (100 MHz, DMSO-d_6) δ 162.5, 160.7, 143.0, 129.9, 119.2, 115.8, 108.4 ppm; $\tilde{\nu}$ max (ATR-IR) 3132 (OH stretch), 3056 (NH stretch), 2922 (CH stretch), 2940 (CH stretch), 1626 (C=O), 1571 (C=C stretch), 1507, 1454, 1432, 1384, 1283, 1187, 1143, 1034, 948, 838, 793, 756 cm-1; *m/z* calcd. for $C_{18}H_{12}N_2O_4$: 320.08, found MS-MALDI-TOF [M] 320.091

Diethyl2,2'-(((3,6-dioxo-2,3,5,6-tetrahydropyrrolo[3,4-c]pyrrole-1,4-diyl)bis(4,1-phenylene))bis(oxy))(2*R*,2'*R*)-dipropionate – (PhO(*R,R*) EP DPP *N*H)

3,6-Bis(4-hydroxyphenyl)-2,5-dihydropyrrolo[3,4-c]pyrrole-1,4-dione(0.40 g, 0.13 mmol), triphenylphosphine (1.32 g, 0.503 mmol) and (−)-ethyl L-lactate (0.60 g, 0.51 mmol) were dissolved in anhydrous tetrahydrofuran (8.5 ml) and cooled to 0°C whilst stirring under an argon atmosphere. Once at this temperature, diisopropyl azodicarboxylate (1.03 g, 0.51 mmol), dissolved in toluene (2.5 ml), was added dropwise and the reaction allowed to warm to room temperature, continuing stirring for 72 hours. Upon completion, excess solvent was removed by rotary evaporator and the remaining solid filtered over a glass sintered crucible (No. 4 porosity). The solid was purified by Soxhlet extraction with acetone. The acetone was removed under reduced pressure and the remaining solid was then recrystallised in boiling ethanol with a hot filtration to yield a red powder. (0.359 g, 69.5 μmol, 53 %). Mp: 296-298 °C; ^{1}H NMR (400 MHz, DMSO d_6) δ 11.16 (bs, 2H), 8.43 (d, *J* = 8.5 Hz, 4H), 7.07 (d, *J* = 8.6 Hz, 4H), 5.17 (q, *J* = 6.7 Hz, 2H), 4.17 (q, *J* = 7.1 Hz, 4H), 1.56 (d, *J* = 6.7 Hz, 6H), 1.20 (t, *J* = 7.1 Hz, 6H) ppm; ^{13}C

[1] Humphreys J, Pop F, Hume PA, Murphy AS, Lewis W, Davies ES, Argent SP, Amabilino DB. Solid state structure and properties of phenyl diketopyrrolopyrrole derivatives. *CrystEngComm*. 2021;23(8):1796–1814.
[2] Ciba Specialty Chemicals Corporation, USPat.,5969154, 1999.

NMR (75 MHz, DMSO *d*6) δ 171.45, 162.95, 160.31, 143.38, 130.16, 121.62, 115.81, 109.68, 72.12, 61.44, 40.83, 40.55, 40.27, 39.99, 39.71, 39.44, 39.16, 18.54, 14.46 ppm; $\tilde{\nu}$ max (ATR-IR) 3134 (NH stretch), 3089 (CH stretch), 3039 (CH stretch), 2985 (CH stretch), 2850 (CH stretch), 1749 (C=O stretch), 1635 (C=O stretch), 1597 (CC stretch), 1512 (CC stretch), 1444, 1257, 1184, 113, 1089, 1041, 947, 835, 786, 758, 624 cm-1; *m/z* calcd. for $C_{28}H_{29}N_2O_8$: 521.192, found MS-MALDI-TOF [M] 521.01; Elemental analysis: calcd. for $C_{28}H_{29}N_2O_8$: C, 64.11; H, 5.42; N, 5.38%; found: C, 63.58; H, 5.25; N, 5.47%.

Diethyl2,2'-(((3,6-dioxo-2,3,5,6-tetrahydropyrrolo[3,4-c]pyrrole-1,4-diyl)bis(4,1-phenylene))bis(oxy))(2*S*,2'*S*)-dipropionate – (PhO(*S,S*) EP DPP *N*H)

The product was synthesised under identical conditions to **PhO(*R,R*) EP DPP *N*H** with the exception of (+)-ethyl D-lactate used instead of (−)-ethyl L-lactate.

Elemental analysis: calcd for $C_{28}H_{29}N_2O_8$: C, 64.11; H, 5.42; N, 5.38%; found: C, 64.54; H, 5.87; N, 5.23%.

2. ^{1}H NMR Spectra

2.1 ^{1}H NMR of **PhDPP-OH-*N*H**

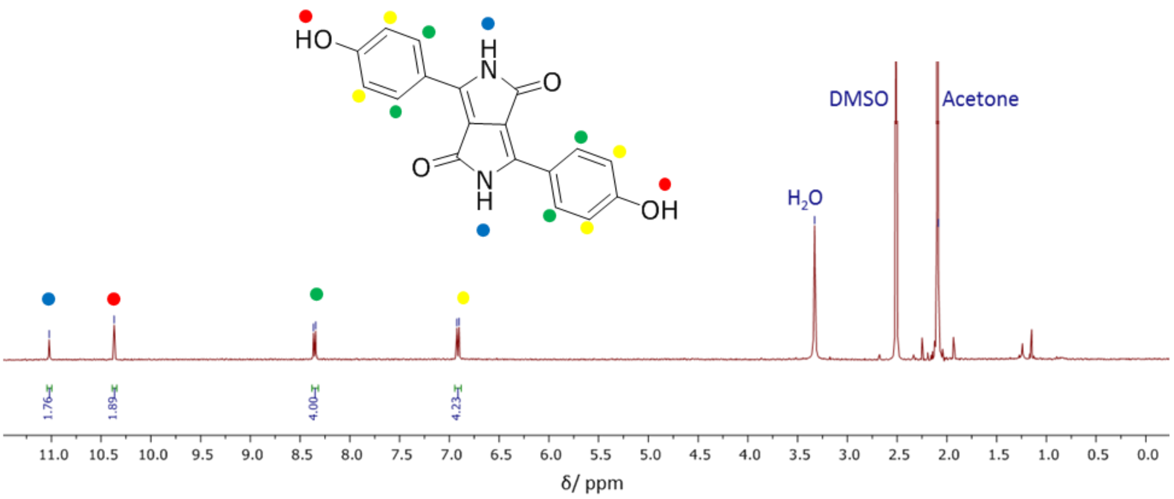

2.2 ^{1}H NMR of **PhO(*S,S*) EP DPP *N*H**

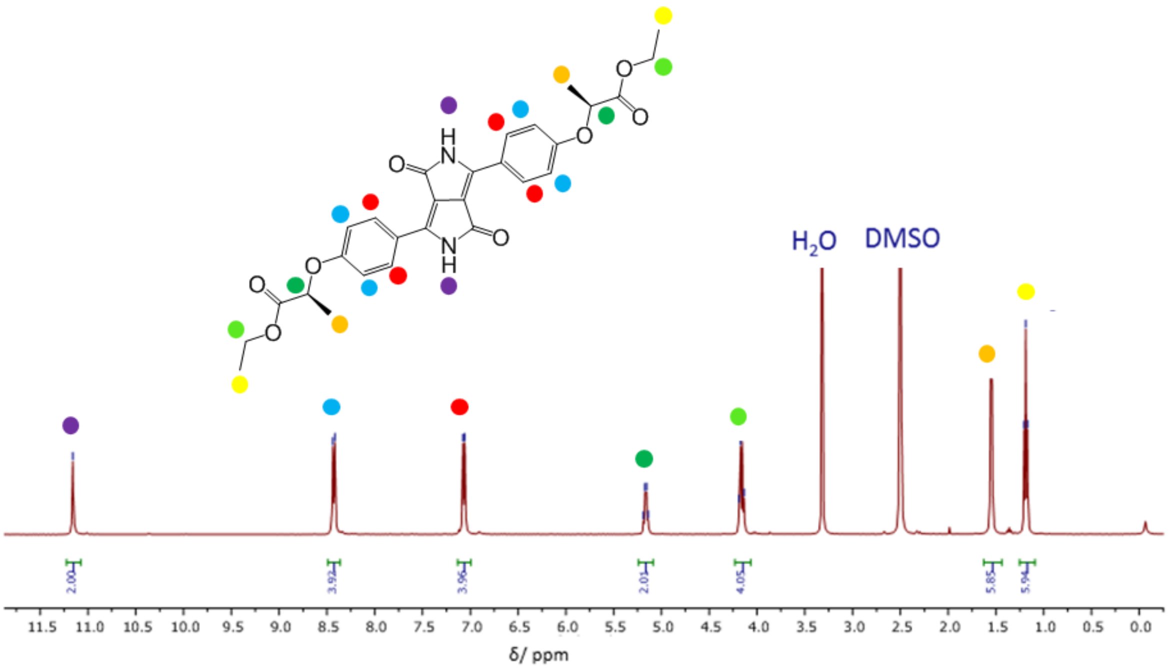

Note : **PhO(*R,R*) EP DPP *N*H** gave identical spectra to **PhO(*S,S*) EP DPP *N*H** so was omitted

3. ^{13}C NMR

3.1 ^{13}C NMR of **PhDPP-OH-*N*H**

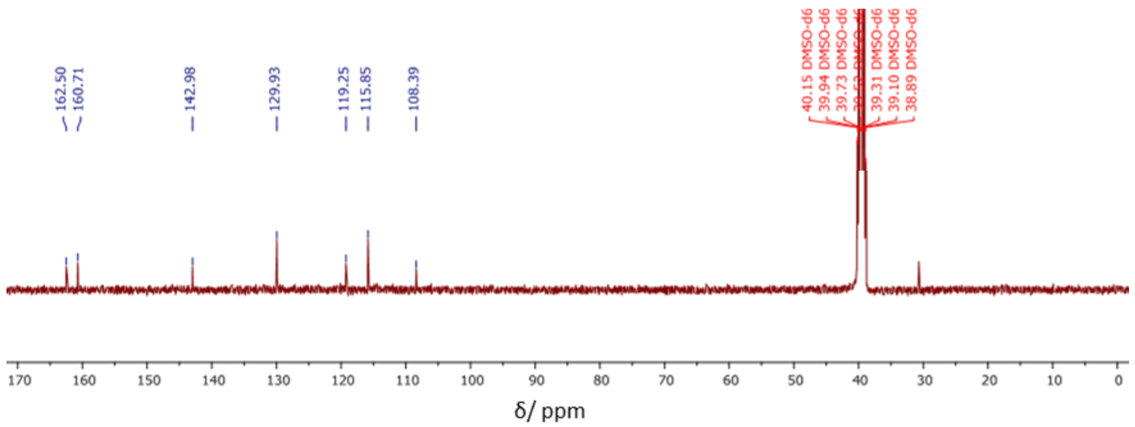

3.2 ^{13}C NMR of **PhO(*S,S*) EP DPP *N*H**

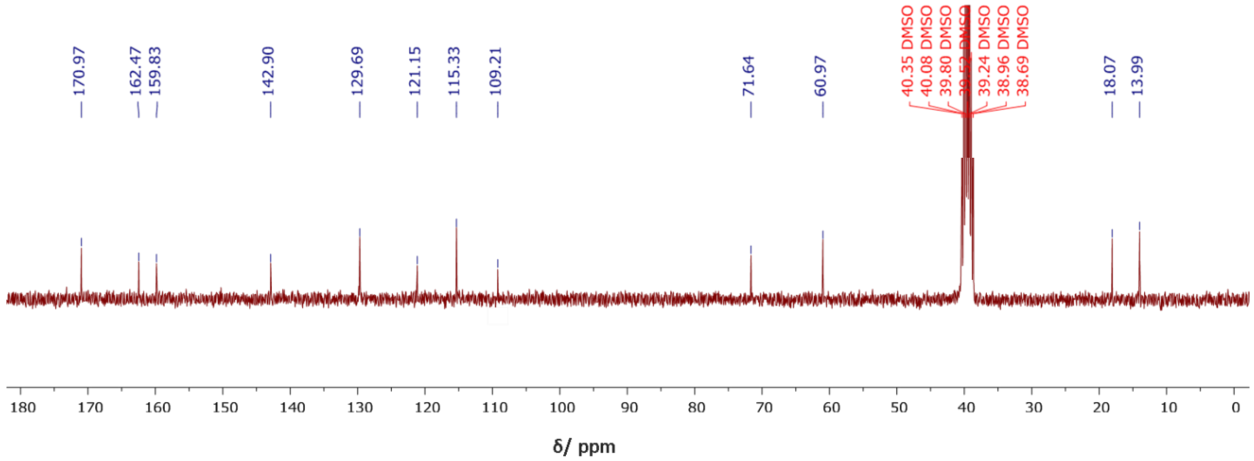

4. FT-IR

4.1 FT-IR of **PhDPP-OH-*N*H**

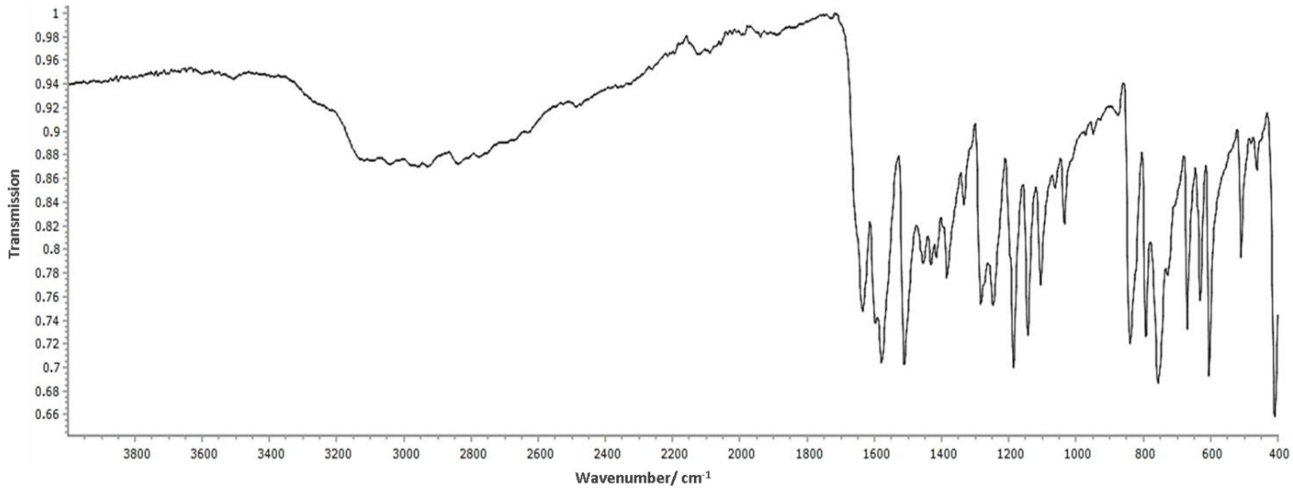

4.2 FT-IR of **PhO(*S,S*) EP DPP *N*H**

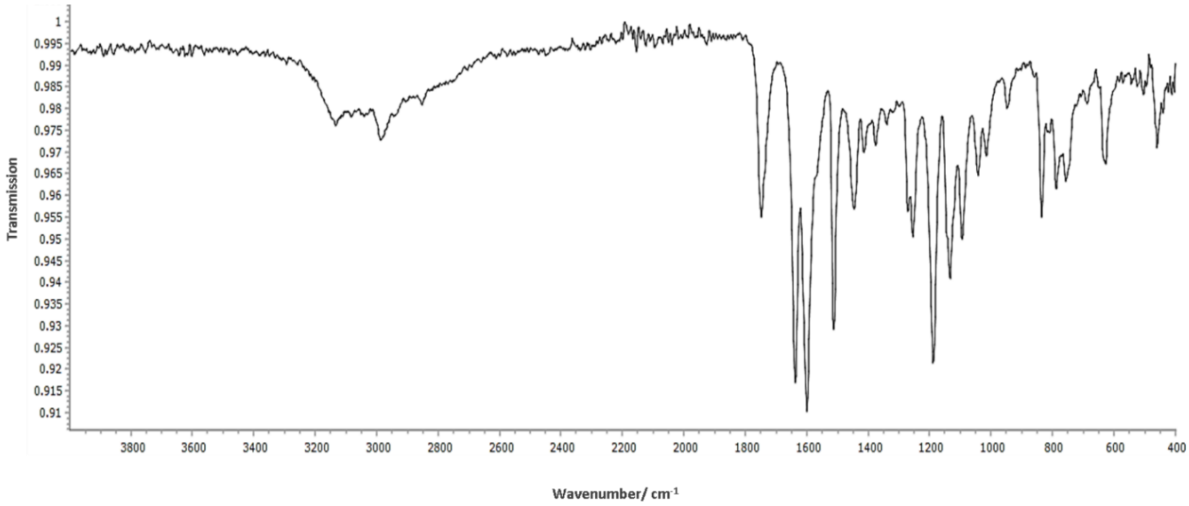

5. Supporting figures

5.1 Structures of **PhDPP** and **ThDPP**

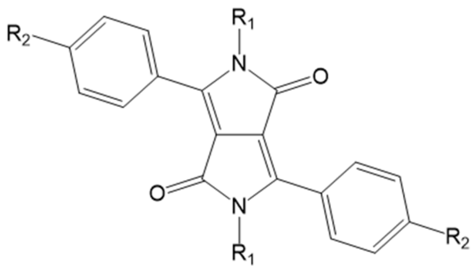

PhDPP

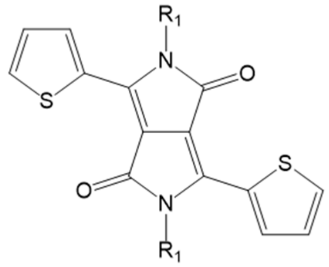

ThDPP

5.2 Characterisation and reactions conditions for the formation of **PhO(*R*,*R*) PCOOH DPP *N*H**

((2*R*,2'*R*)-2,2'-(((3,6-Dioxo-2,3,5,6-tetrahydropyrrolo[3,4-c]pyrrole-1,4-diyl)bis(4,1-phenylene))bis(oxy))dipropionic acid)

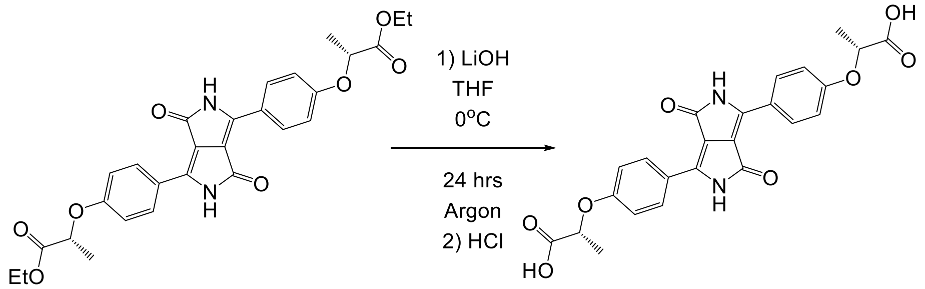

PhO(*R*,*R*) EP DPP *N*H

PhO(*R*,*R*) PCOOH DPP *N*H
69%

PhO(*R*,*R*) EP DPP *N*H (0.45g, 0.088mmol) was dissolved in THF (15 ml) and cooled to 0°C, whilst stirring under an argon atmosphere. Once at this temperature lithium hydroxide (0.23g, 0.96mmol) dissolved in water (7.5 ml) was added dropwise and the reaction allowed to warm to room temperature and continued to stir overnight. Upon completion, the reaction mixture was acidified to pH 2 using 2M hydrochloric acid, followed by extraction of the organic layer with tetrahydrofuran and sodium chloride. The organic layer was dried ($MgSO_4$), filtered and excess solvent removed on rotary evaporator to yield the pure product, a red solid (0.28 g, 0.60 mmol, 68.7%). The product is novel and has: Mp: >250°C; ESI-MS found: $[M-H]^-$ 463.1154 and $C_{24}H_{19}N_2O_8$ requires M 463.1147; ν_{max} (ATR-IR) 3485 (V.broad OH stretch), 3417 (V.broad NH stretch), 3386, 3302, 3277, 3242, 3110, 3063, 2970 (CH stretch), 2851 (CH stretch), 1741 (C=O), 1636(C=O DPP), 1594 (C=C stretch), 1508, 1444, 1409, 1370,

1250, 1187, 1143, 1088, 1036, 946, 876, 834, 789, 740, 674, 624, 540, 503, 461 cm^{-1}; ^{1}H NMR (300 MHz, DMSO-d_6) δ 11.15 (2H, s, NH), 8.43 (4 H, d, *J* 9.0 Hz, ArH), 7.05 (4 H, d, *J* 9.0 Hz, ArH), 5.03 (2H, q, *J* 6.7 Hz, R-CH), 1.54 (4 H, d, *J* 6.8 Hz , R-CH_3) ppm; ^{13}C NMR (75 MHz, DMSO-d_6) δ 172.6, 162.5, 160.1, 142.9, 129.7, 120.93, 115.2, 109.1, 71.6, 18.1 ppm

^{1}H NMR of **PhO(*R*,*R*) PCOOH DPP *N*H**

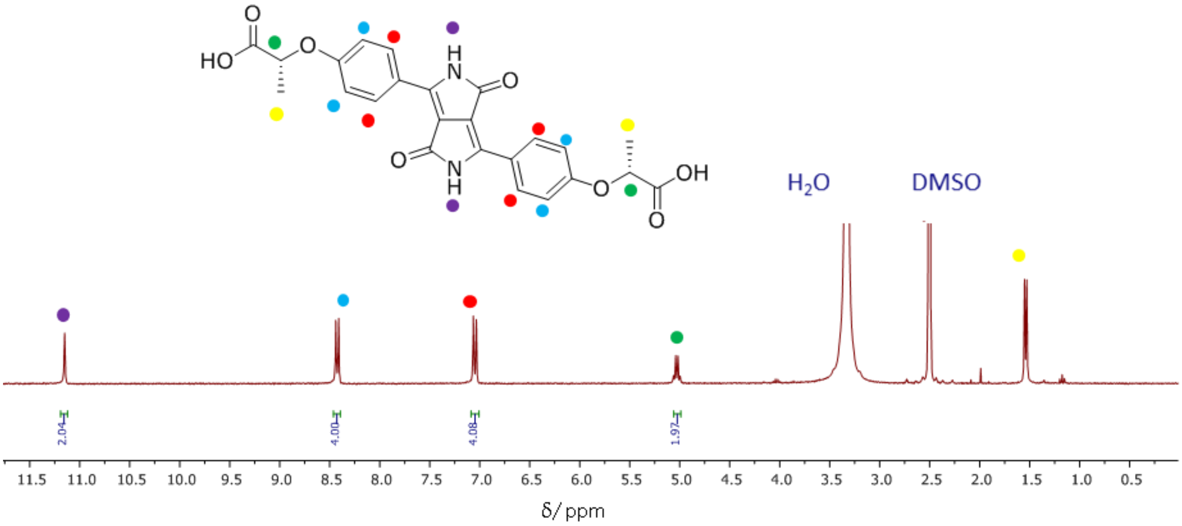

^{13}C NMR of **PhO(*R*,*R*) PCOOH DPP *N*H**

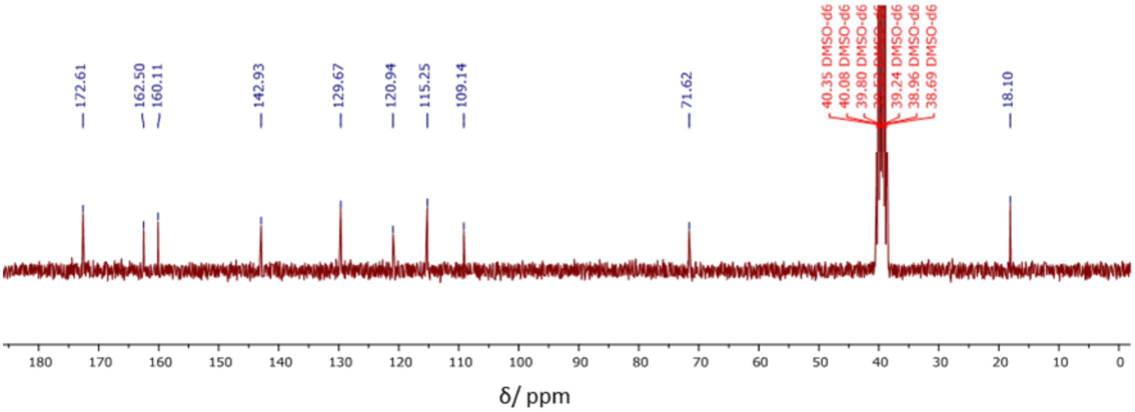

5.3 UV/Vis absorption spectrum of **PhO(*R*,*R*) EP DPP *N*H** in THF.

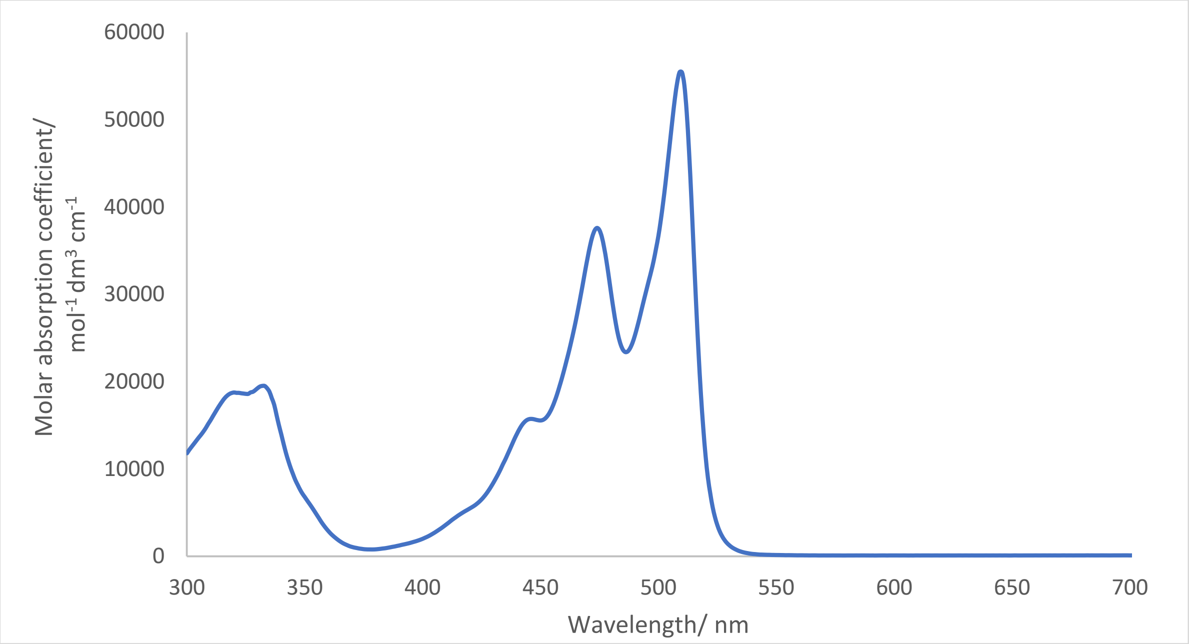

5.4 Table of the photophysical properties of **PhO(*R*,*R*) EP DPP *N*H** in THF.

**Compound**	**Absorption λ_{max} [nm]**	**ε [dm^{3}mol^{-1} cm^{-1}]**	**Emission λ_{max} [nm]**	**Stokes shift [nm]**	**Φ**
**PhO(*R*,*R*) EP DPP *N*H**	509	54,100	515	6	0.89

[a] Excitation wavelength 470 nm. [b]Quantum yields were calculated by comparison with the fluorescence observed for fluorescein (F = 91 in NaOH) under identical conditions of irradiation.

5.5 Solution and thin film UV/Vis absorption spectra of **PhO(*S,S*) EP DPP *NH*** in THF.

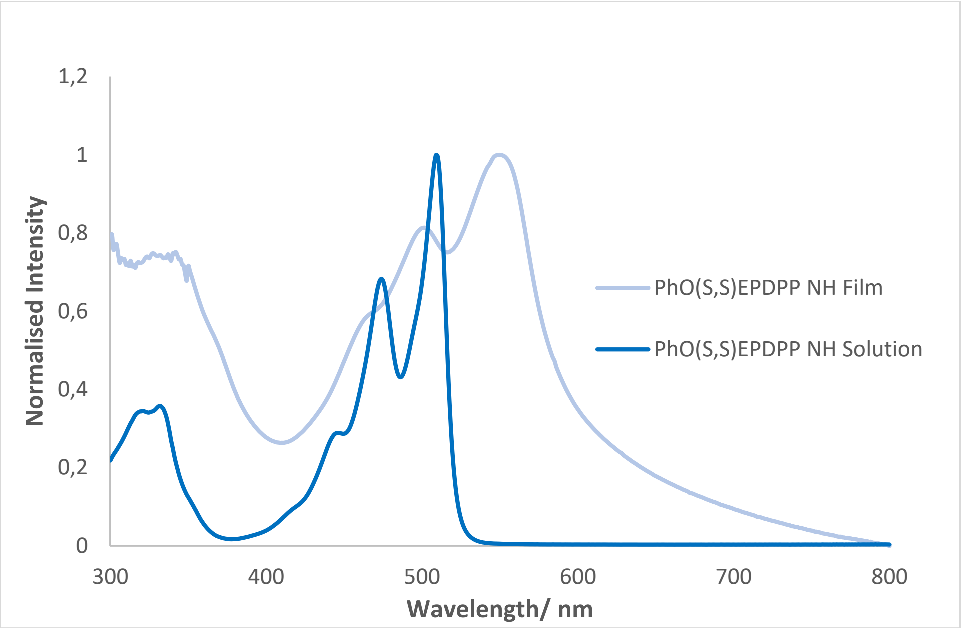

5.6. Table of solution and solid state absorption properties of **PhO(*R,R*) EP DPP *NH*.**

**Compound**	**Absorption λ_{max} solution [nm]**	**Absorption λ_{max} solid [nm]**	**Absorption edge onset film [nm]**	**Optical Eg film [eV]**
**PhO(*R,R*) EP DPP *NH***	509	551	602	2.06

. [a]Thin films were formed on glass slides by drop casting of a concentrated THF solution (0.5 mg ml^{-1})

Electrochemistry

5.7 Full sweep cyclic voltammogram of **PhO(*R,R*) EP DPP *N*H** at 100 mV/s scan rate (referenced against potential of Fc/Fc$^+$) in DMF with tetrabutylammonium hexafluorophosphate as supporting electrolyte. The enantiomer shows identical behaviour.

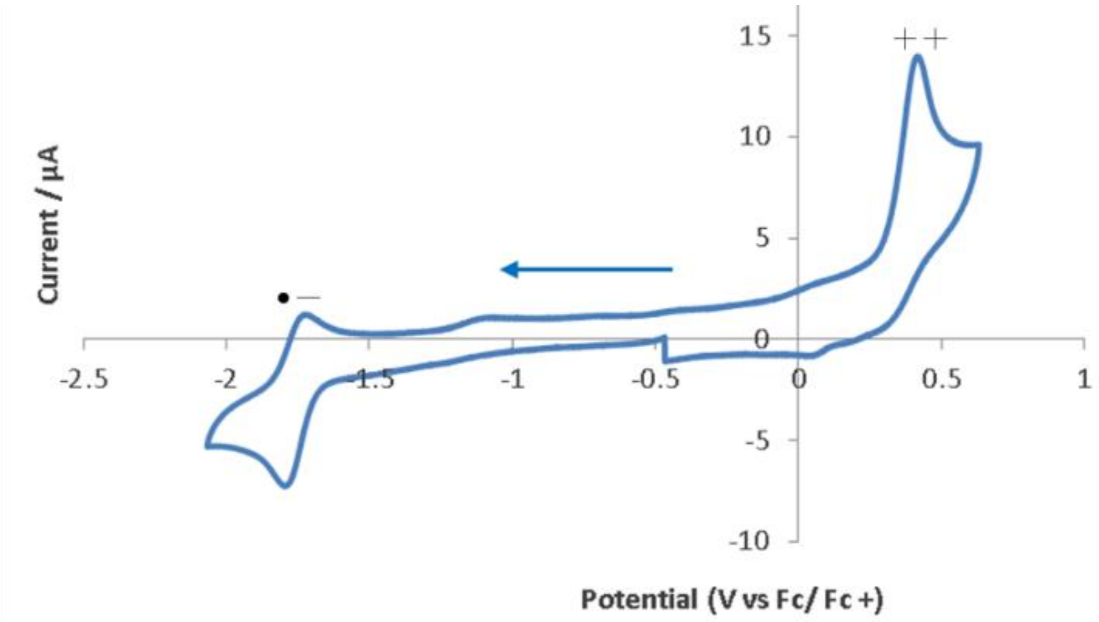

5.8 Scan rate dependence study of the oxidation process for **PhO(*R,R*) EP DPP *N*H**.

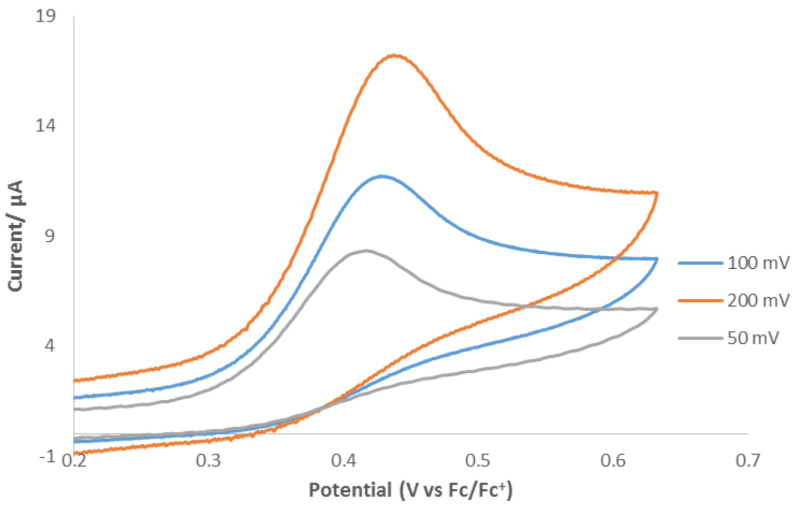

5.9 Scan rate dependence study of the reduction process for **PhO(*R*,*R*) EP DPP *N*H**.

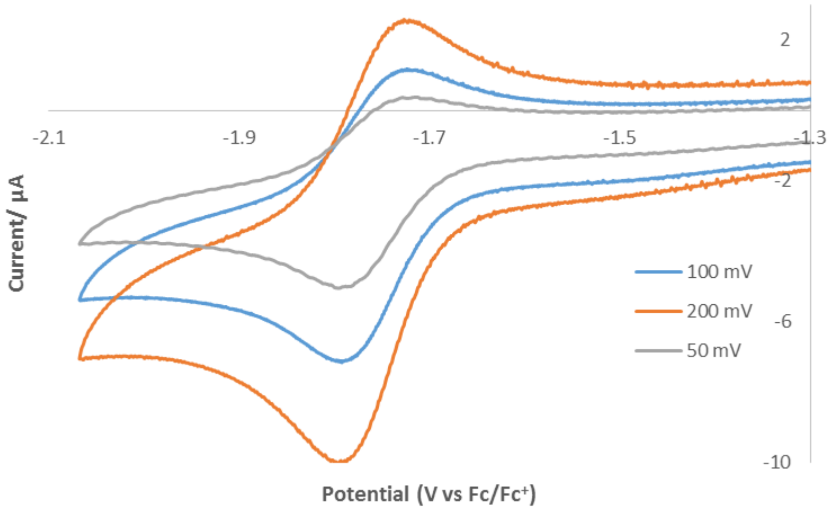

5.10 Table of potentials for oxidation and reduction processes for **PhO(*R*,*R*) EP DPP *N*H** Potentials in V versus Fc^+ / Fc.

Scan rate [mV s^{-1}]	$E_{pa\ (OX)}$ [V]	$E_{pa\ (RED)}$ [V]	$E_{pc\ (RED)}$ [V]	$E_{pa} – E_{pc}$ (RED) [V]	$E_{1/2}$ Red [V]
**200**	0.436026	-1.72706	-1.79542	0.0684	-1.761
**100**	0.427481	-1.72386	-1.79222	0.0784	-1.758
**50**	0.417868	-1.71531	-1.79542	0.0801	-1.755

5.11 Structures of literature compounds **PhDPP NH**, **PhClDPP NH** and **PhBrDPP NH**.

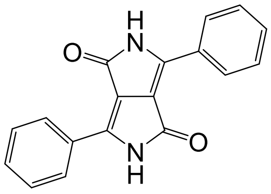

PhDPP NH

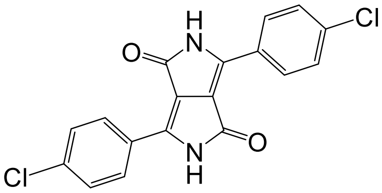

PhClDPP NH

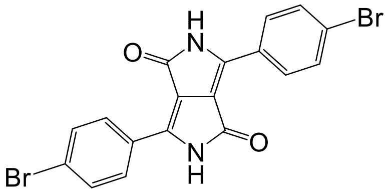

PhBrDPP NH

5.12. Table of the minimum concentration and initial growth temperature for formation of helical aggregates visible by optical microscopy for **PhO(*R,R*) EP DPP *N*H** and **PhO(*S,S*) EP DPP *N*H**.

**Solvent**	**Minimum concentration for helical aggregates visible twisting via optical microscope / mg ml^{-1}**	**Initial growth temperature / ^{o} C**
**Acetone**	0.2 [a]	50 -35 [b]
**Methanol**	0.1 [a]	59-38 [b]
**Acetonitrile**	0.1 [a]	71 [b]

[a] Concentrations were determined using cooling rate of 1 ^{o}C /min for both **PhO(*R,R*) EP DPP *N*H** and **PhO(*S,S*) EP DPP *N*H**.

[b] Temperatures were determined after several repeats with both **PhO(*R,R*) EP DPP *N*H** and **PhO(*S,S*) EP DPP *N*H**.

5.13 Growth initiation temperature study of the formation of helical aggregates in acetone.

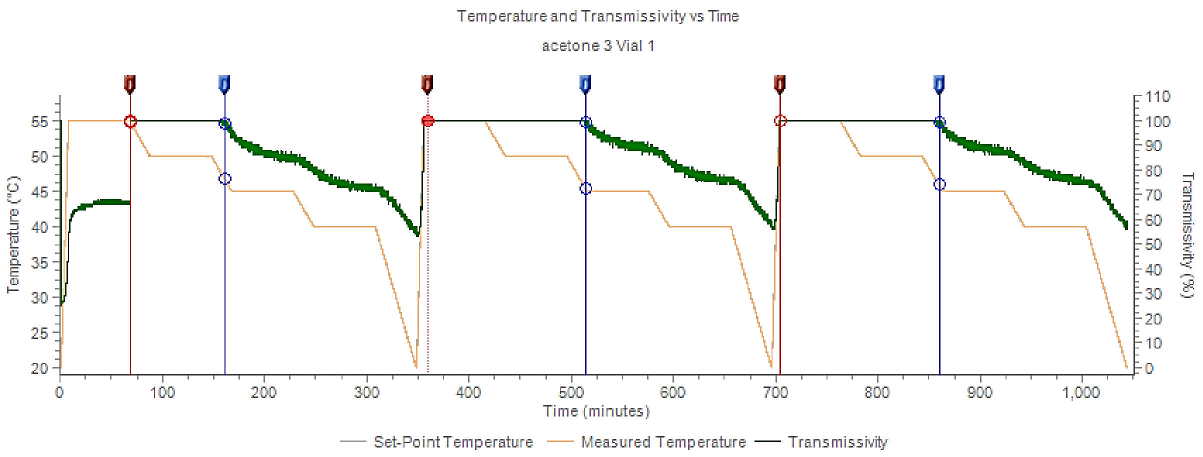

	Event	Time	Temperature	Transmissivity
	Clear	68.92	54.8	100
	Cloud	161.27	46.8	98.51
	Clear	359.76	55.1	100
	Cloud	514.32	45.34	99.35
	Clear	704.35	55.1	100
	Cloud	859.43	45.92	98.96

5.14 Experimental set up of vapour diffusion to form helical aggregates.

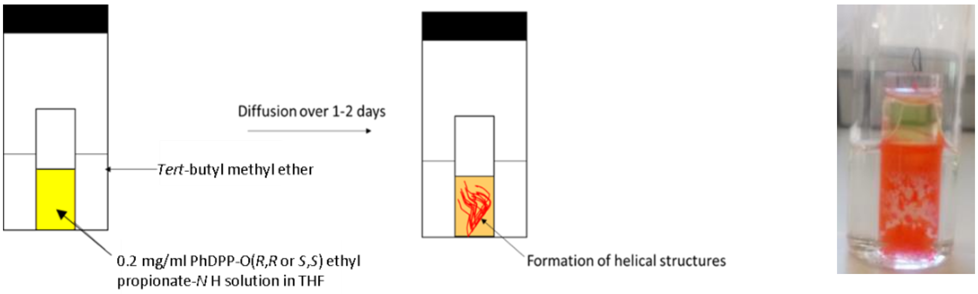

5.15 Optical microscope image in transmission mode of **PhO(*S,S*) EP DPP NH** fibres. , grown from an acetone solution under variable temperature conditions showing the general morphology of the fibres.

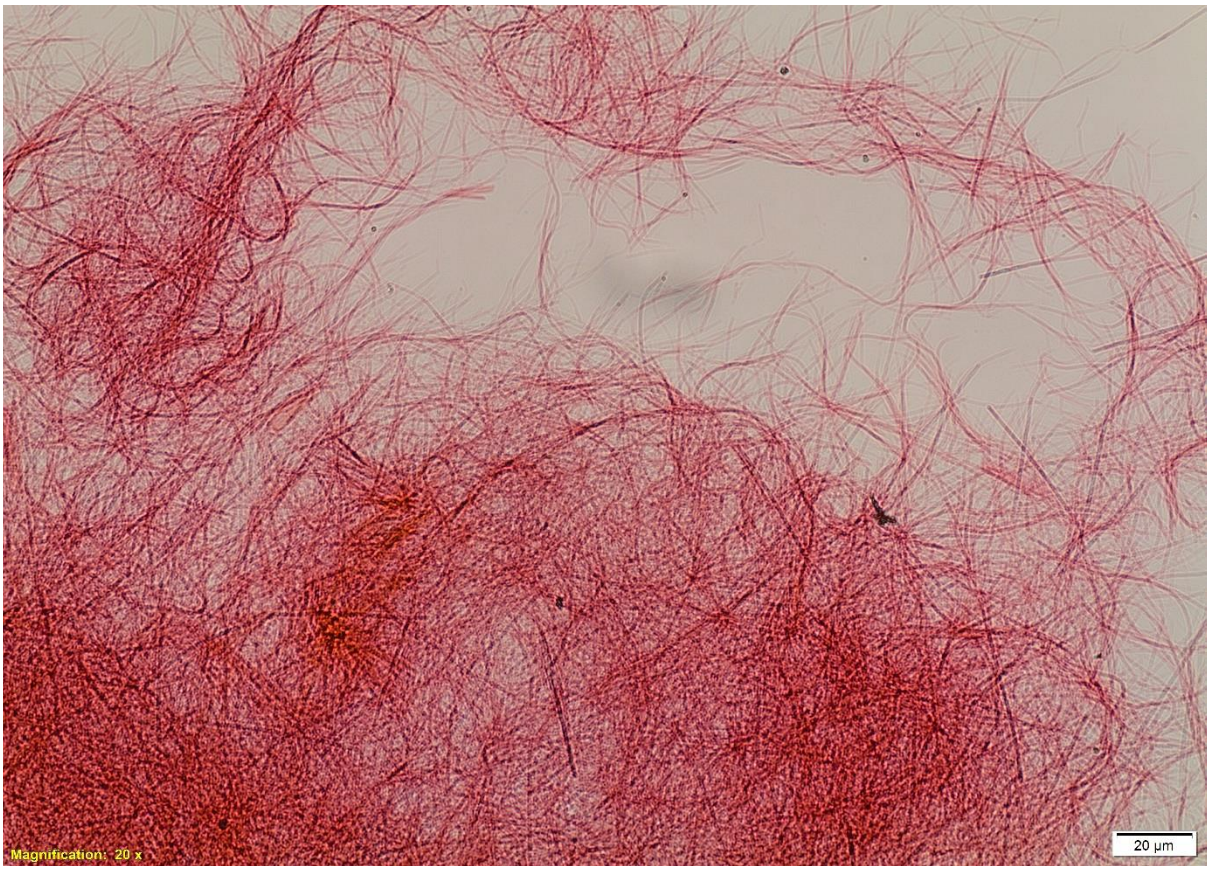

5.16 Optical microscope image in reflectance mode of **PhO(*S*,*S*) EP DPP NH**, grown from an acetone solution under variable temperature conditions showing the general morphology and twisting of the fibres.

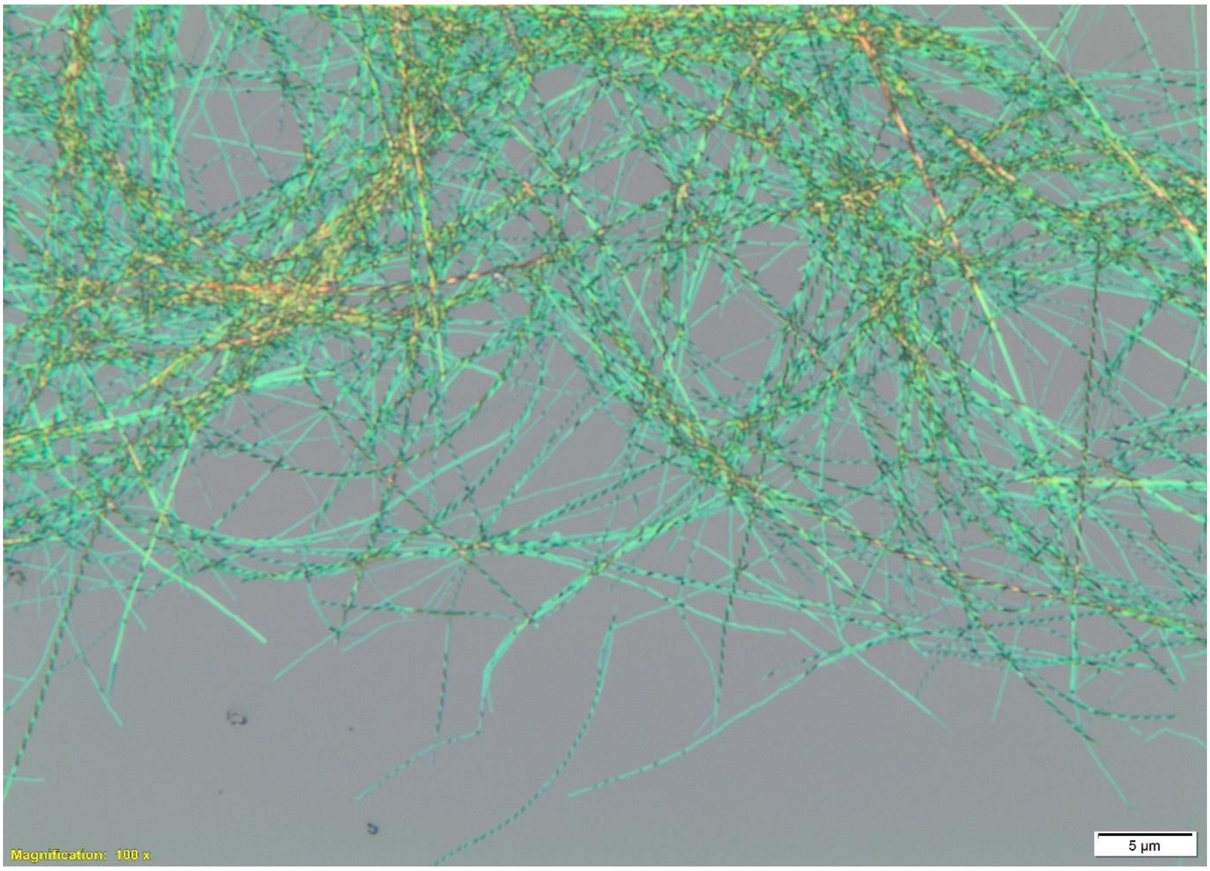

5.17. Optical microscope image in reflectance mode of **PhO(*R*,*R*) EP DPP NH**, grown from an acetone solution under variable temperature conditions showing the general morphology and twisting of the fibres.

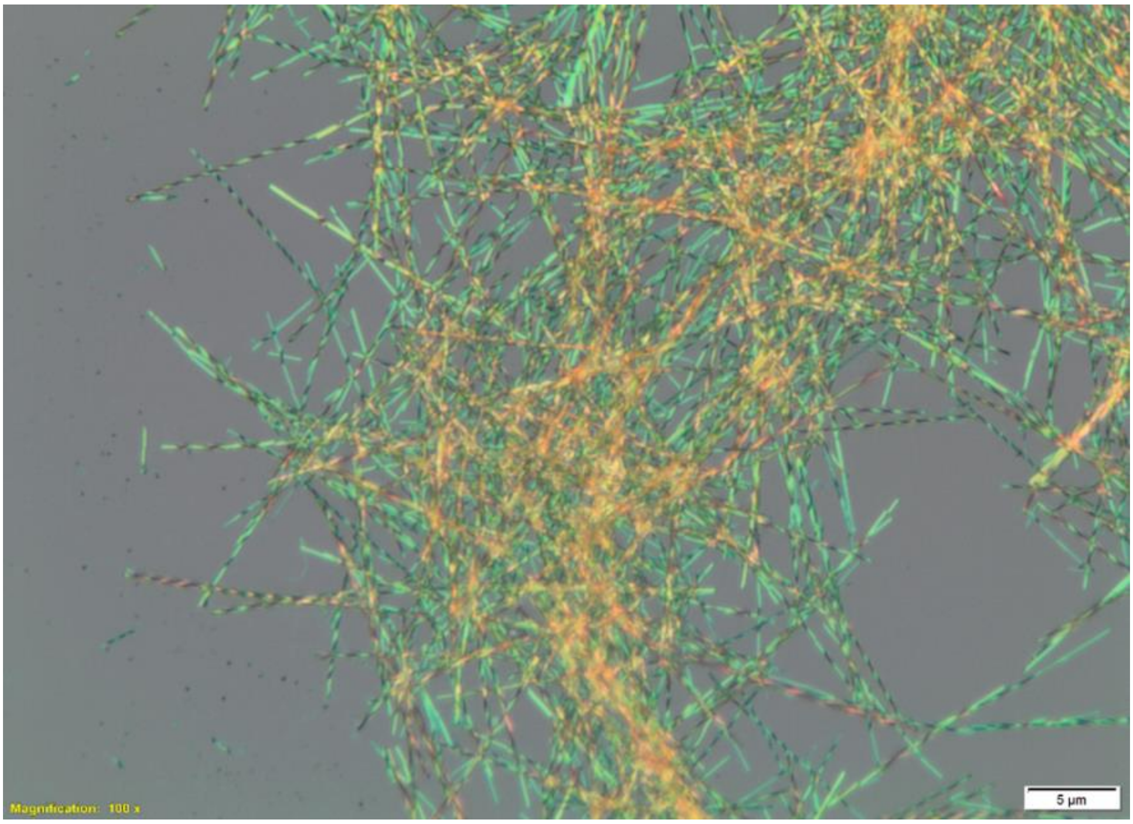

5.18. Optical microscope image in reflectance mode of **PhO(*R*,*R*) EP DPP NH,** grown from an acetonitrile solution under variable temperature conditions showing the general morphology and twisting of the fibres.

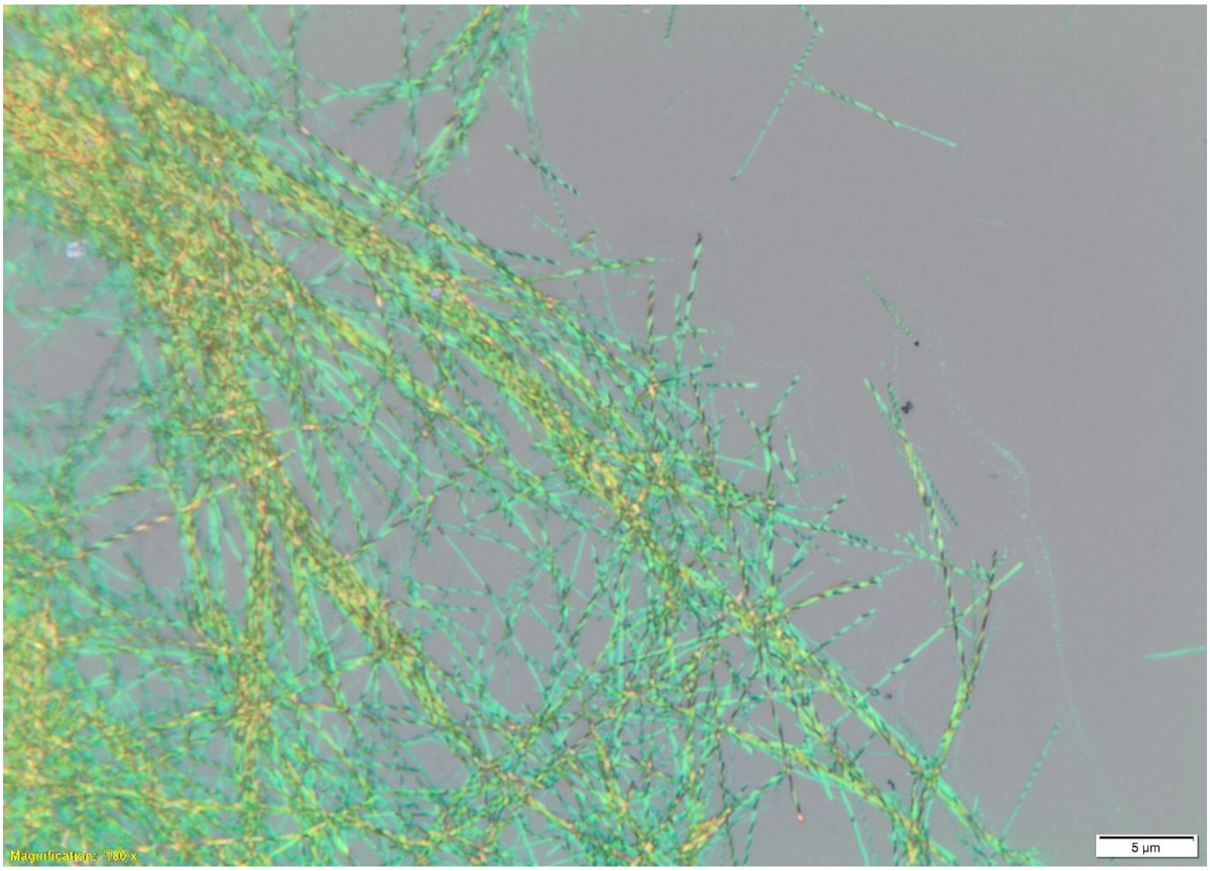

5.19. Optical microscope image in reflectance mode of **PhO(*R*,*R*) EP DPP NH**, grown from a methanol solution under variable temperature conditions showing the general morphology and twisting of the fibres.

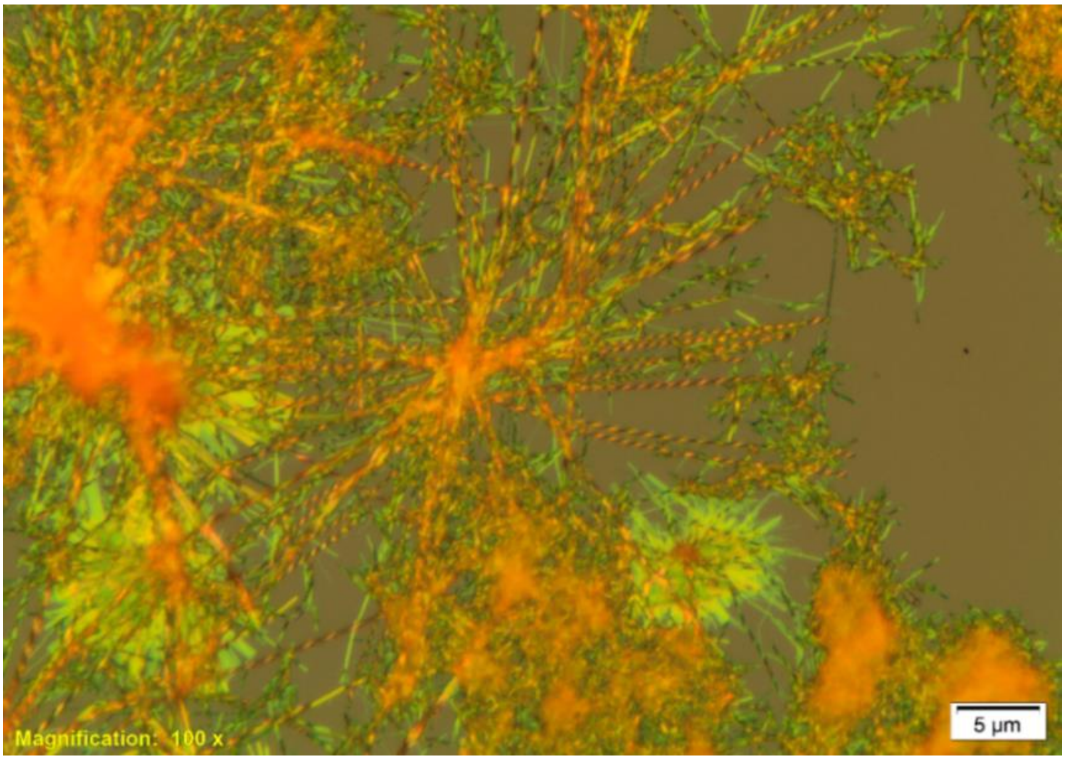

5.20. Optical microscope image in polarised transmission mode of **PhO(*R*,*R*) EP DPP NH**, grown from a methanol solution under variable temperature conditions showing the general morphology and twisting of the fibres.

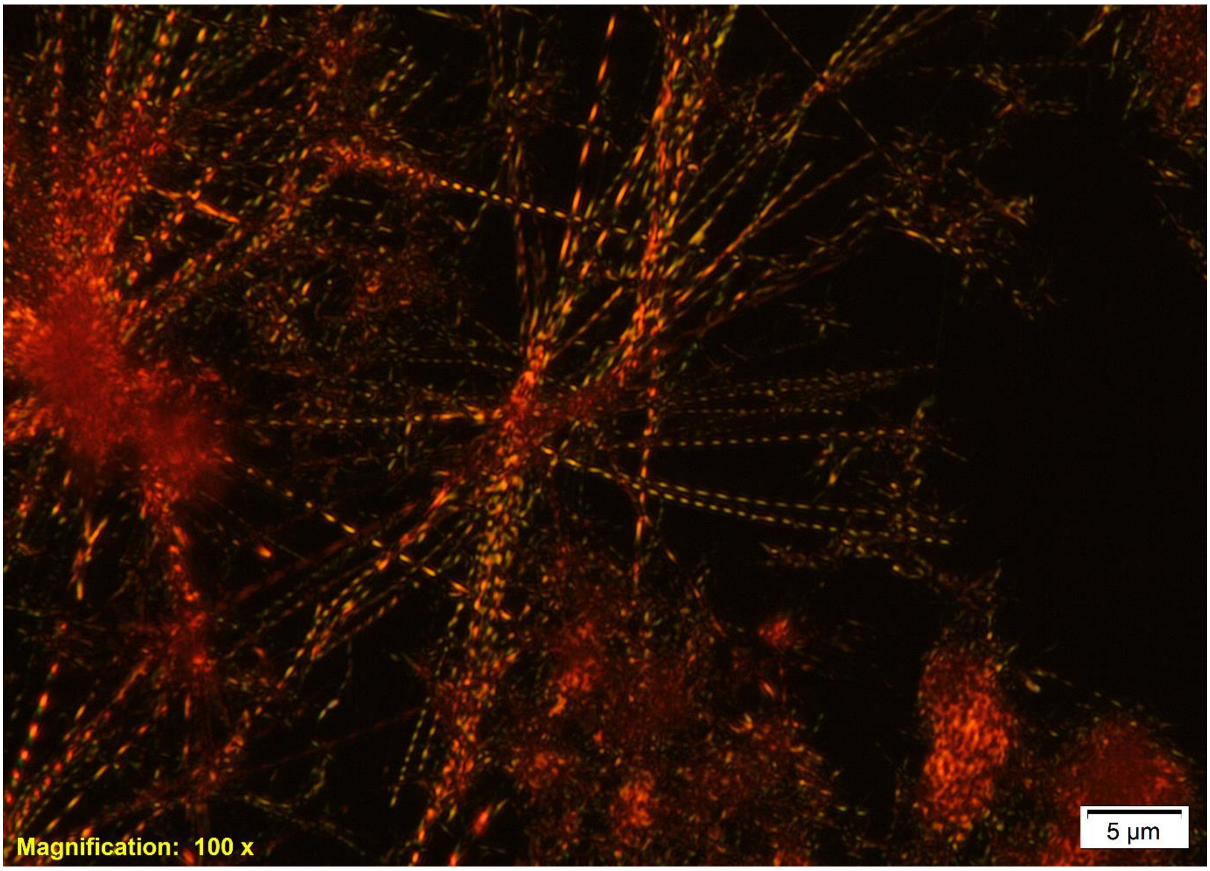

5.21. Optical microscope image of **PhO(*R,R*) EP DPP NH** in transmission and polarised transmission showing the general morphology and twisting of the fibres grown under vapour diffusion conditions

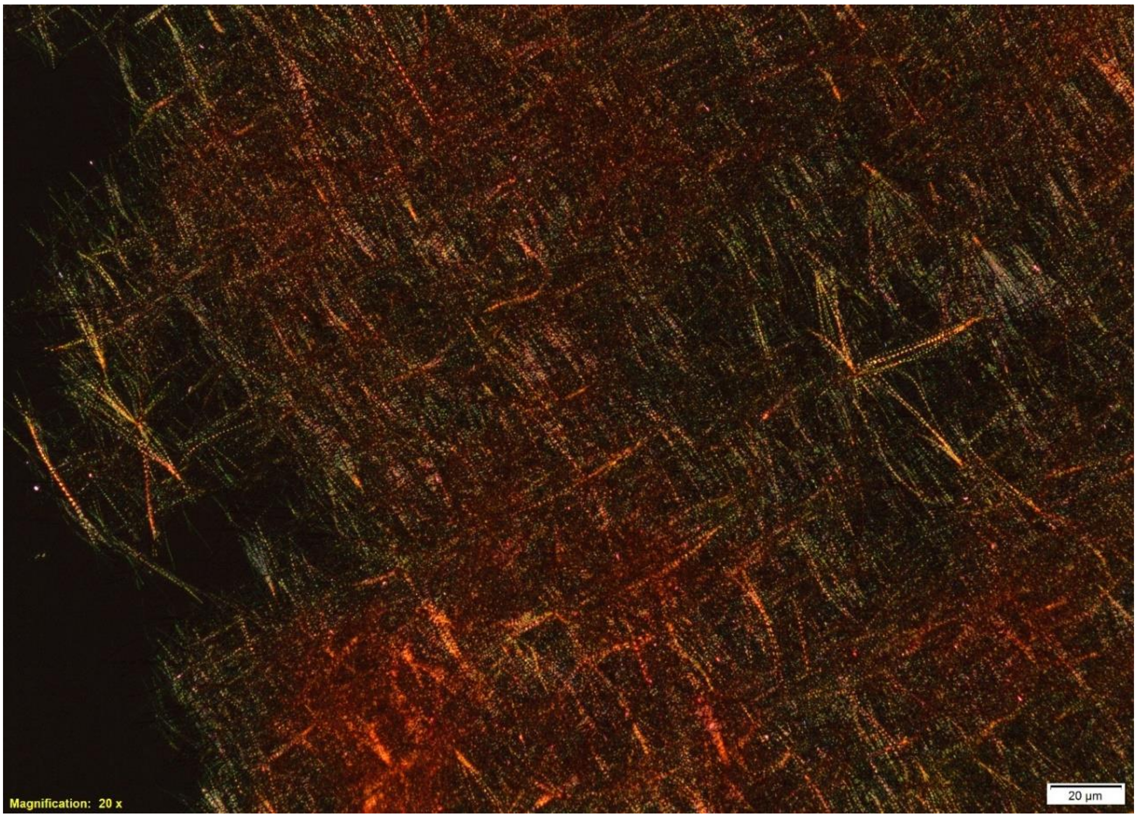

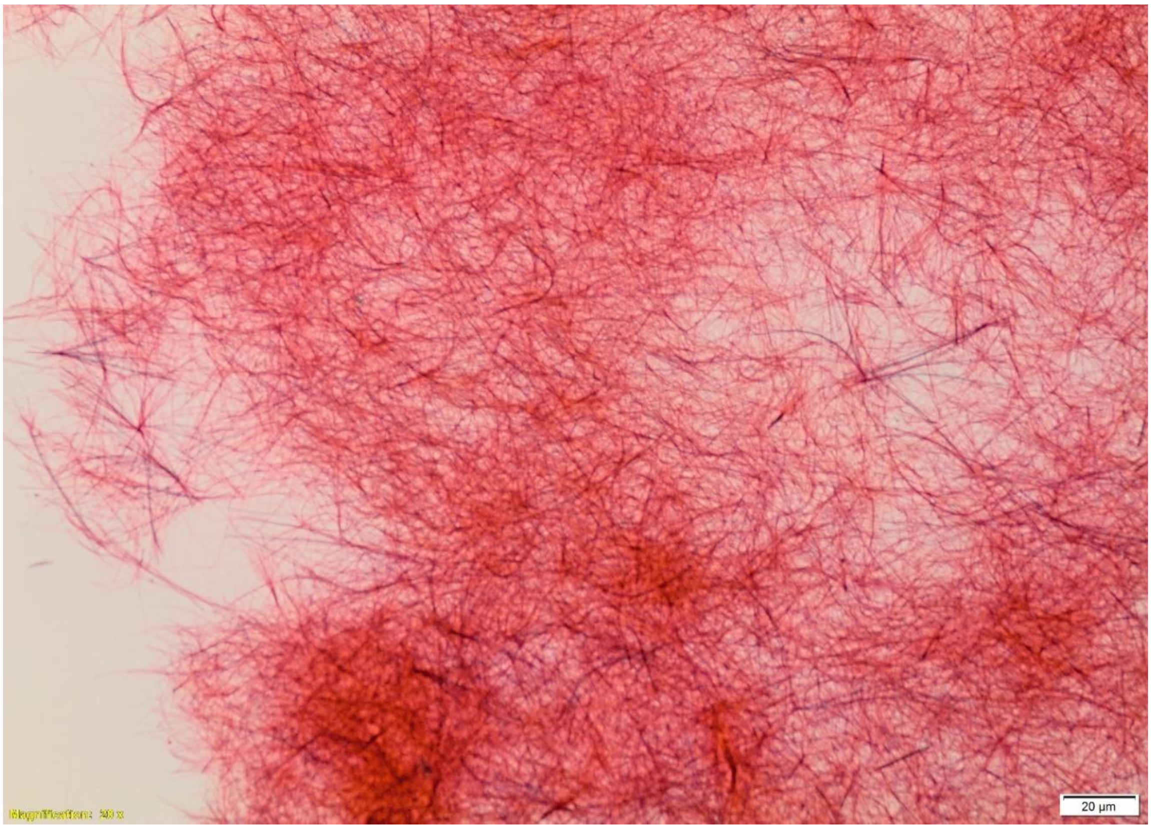

5.22. Optical microscope image of **PhO(*R,R*) EP DPP NH** in transmission and polarised transmission showing the general morphology and twisting of the fibres grown under vapour diffusion conditions.

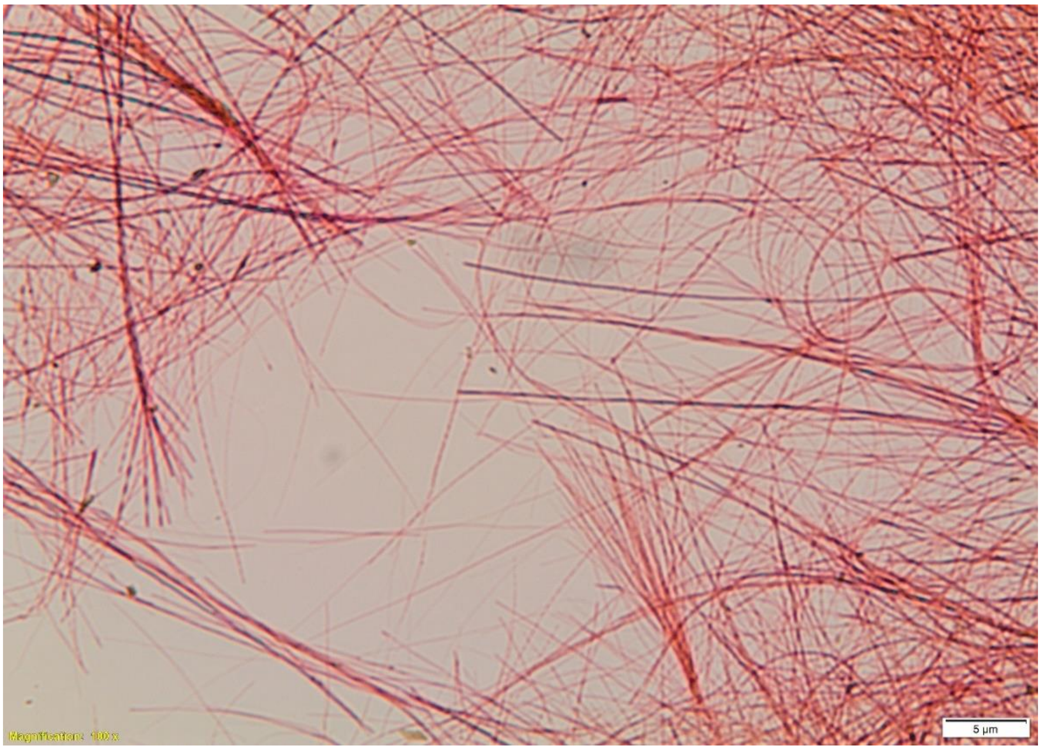

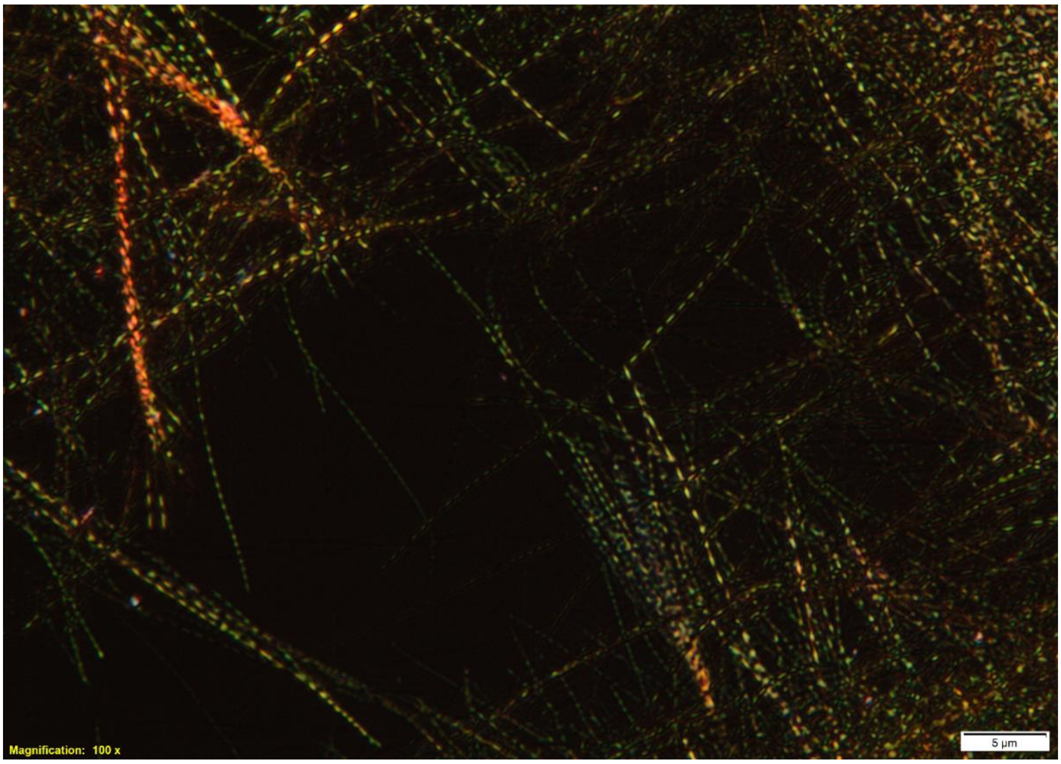

5.23**.** Optical microscope image of **PhO(*S*,*S*) EP DPP NH** in transmission and polarised transmission showing the general morphology and twisting of the fibres grown under vapour diffusion conditions.

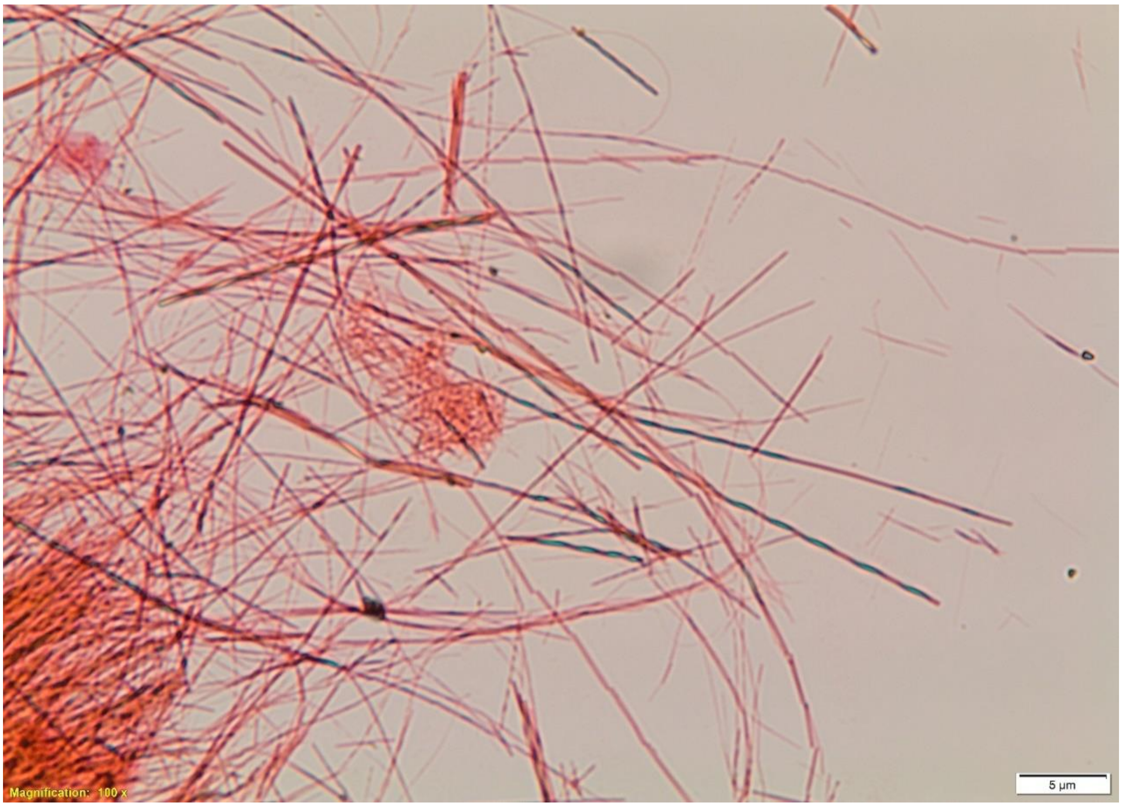

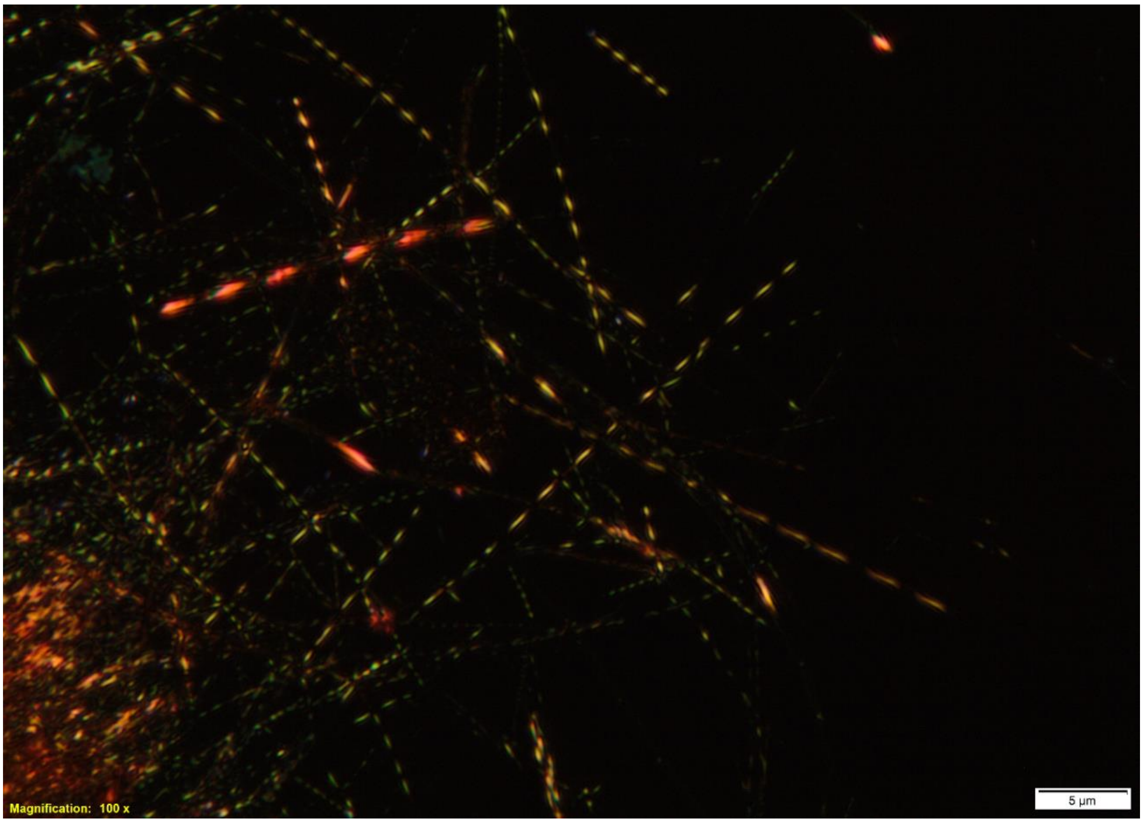

5.24. Optical microscope images of **PhO(*S*,*S*) EP DPP NH** in reflectance and polarised transmission showing a twisted fibre and a fractured one.

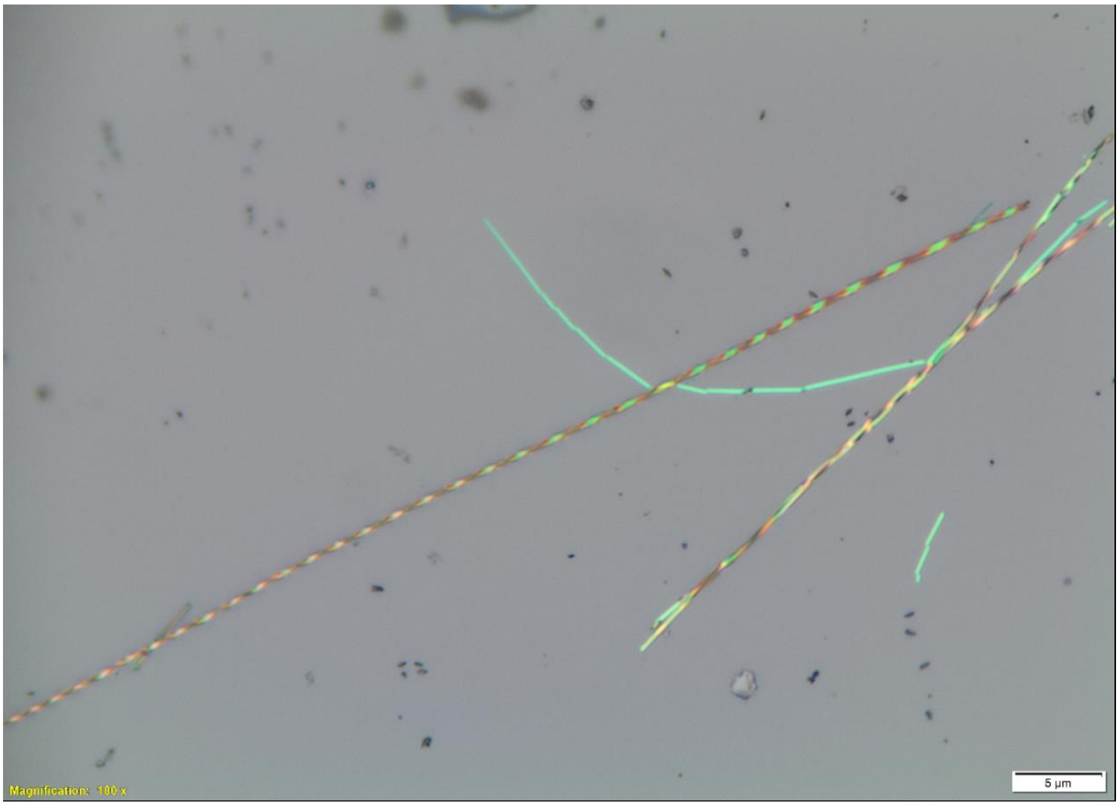

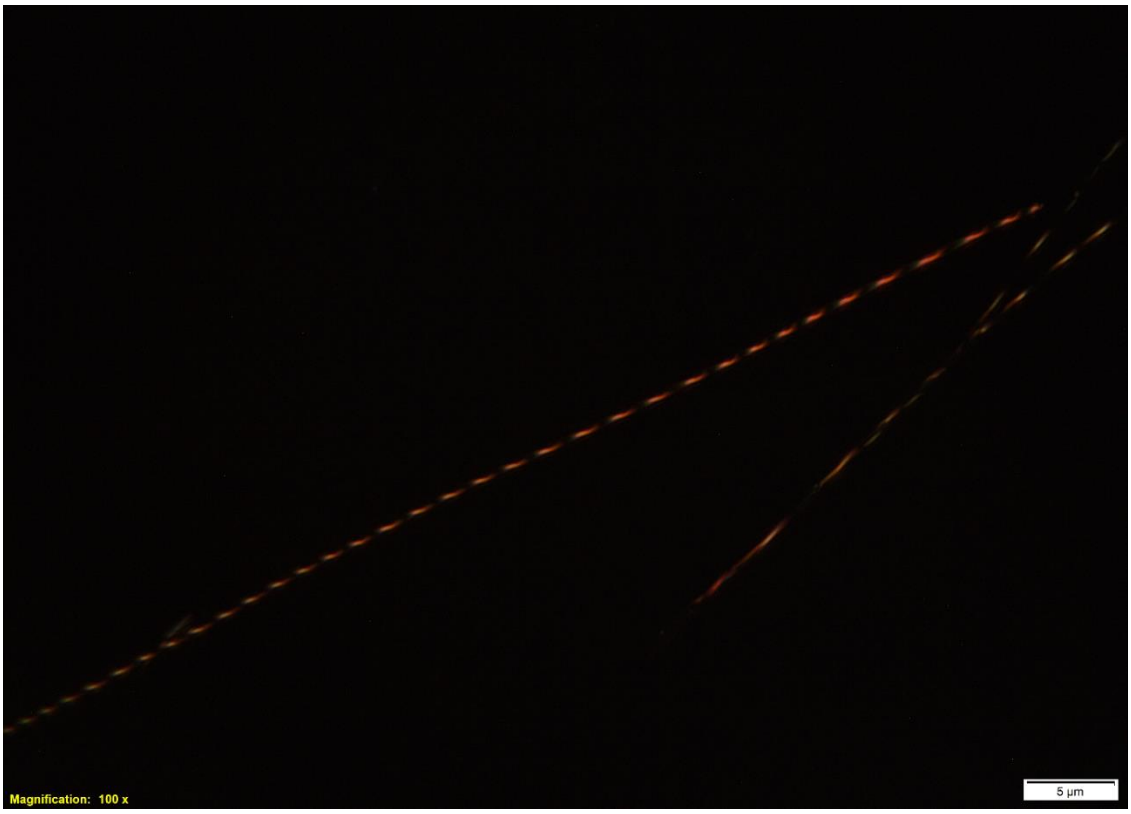

5.25. Optical microscope image in reflectance of a 50:50 mixture of **PhO(*R*,*R*) EP DPP NH** and **PhO(*S*,*S*) EP DPP NH** grown via vapour diffusion showing the general morphology of the aggregates.

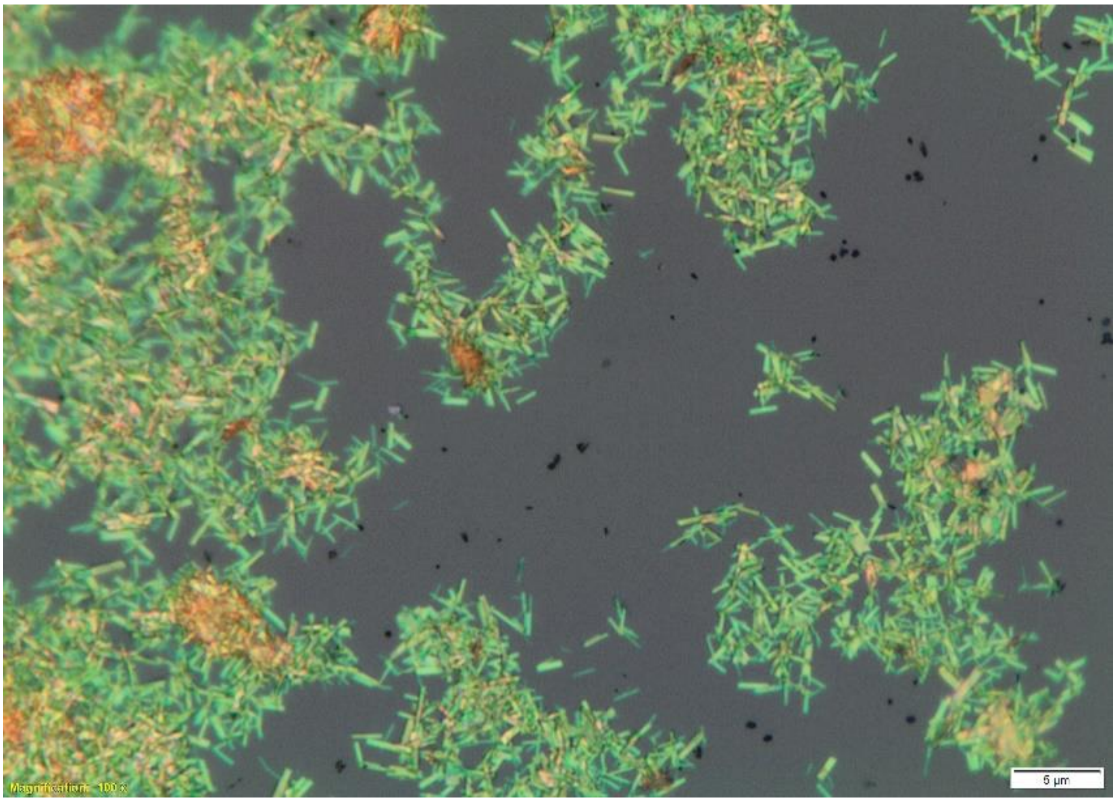

5.26. SEM image of **PhO(*R*,*R*) EP DPP NH** grown from an acetonitrile solution under variable temperature conditions showing the general morphology and twisting of the fibres.

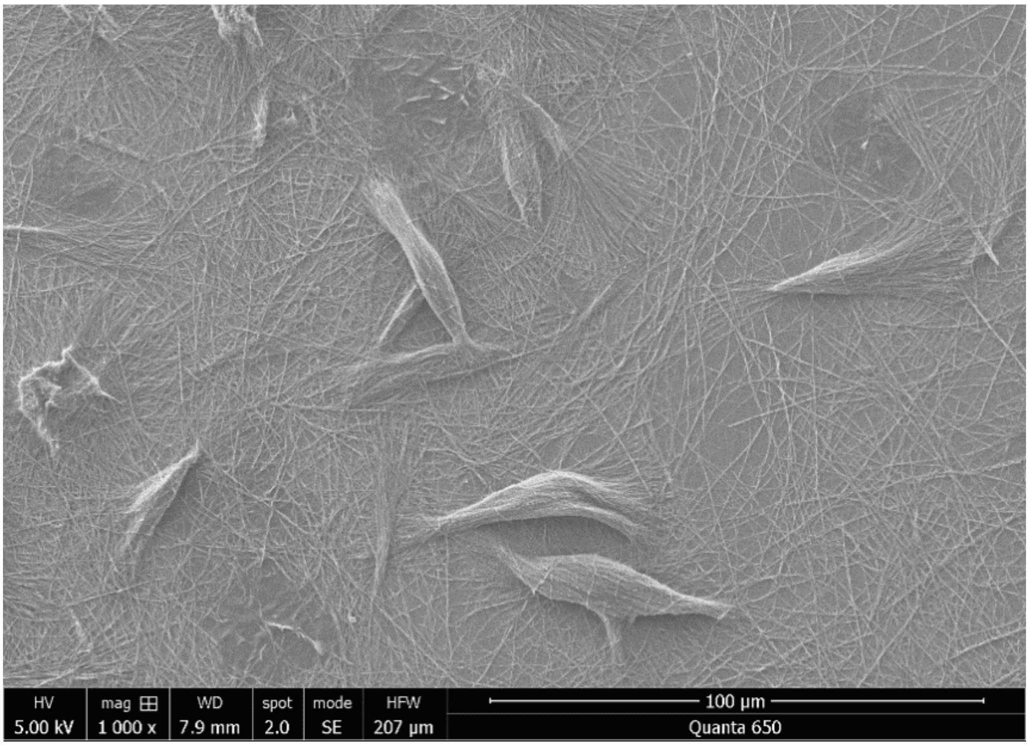

5.27. SEM image of **PhO(*R,R*) EP DPP NH** grown from an acetonitrile solution under variable temperature conditions showing the general morphology and twisting of the fibres.

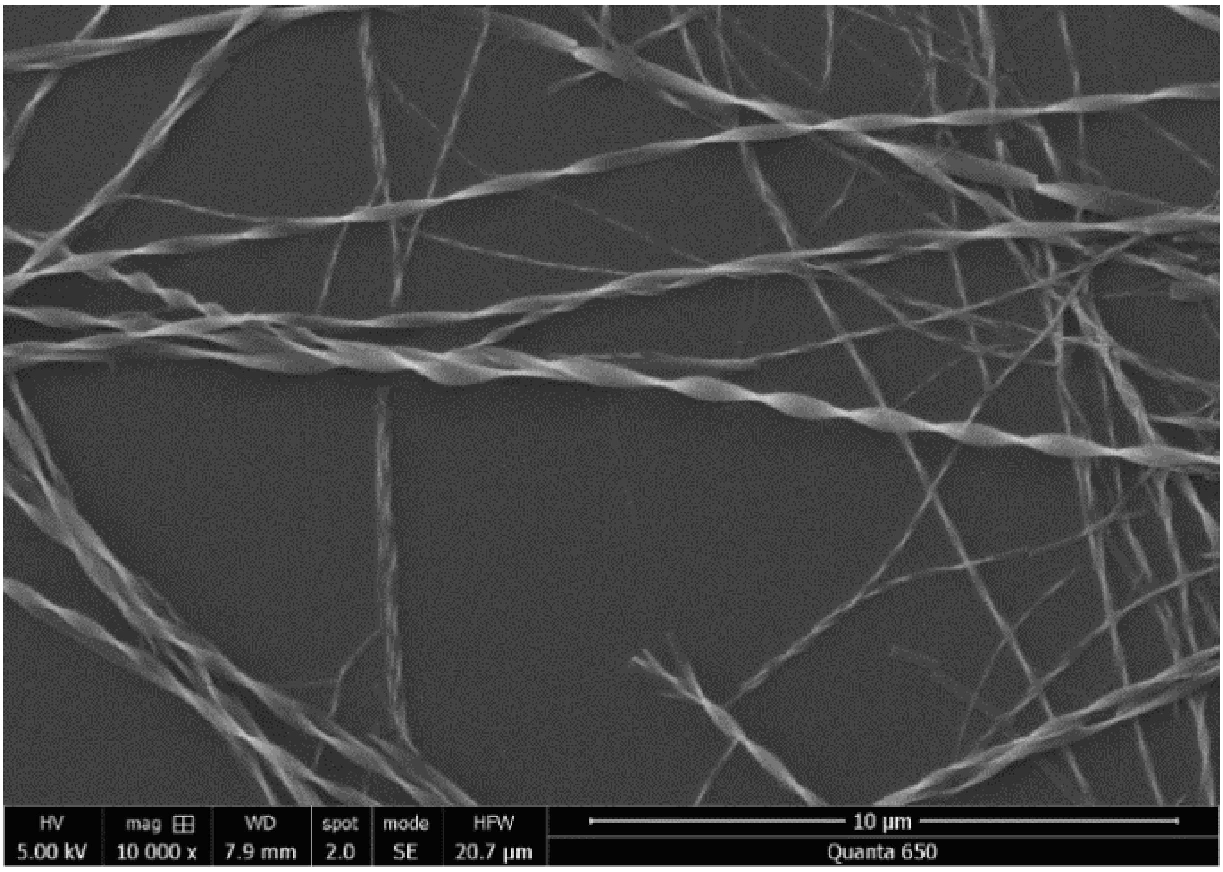

5.28. SEM image of **PhO(*S,S*) EP DPP NH**, grown from an acetonitrile solution under variable temperature conditions showing the general morphology and twisting of the fibres.

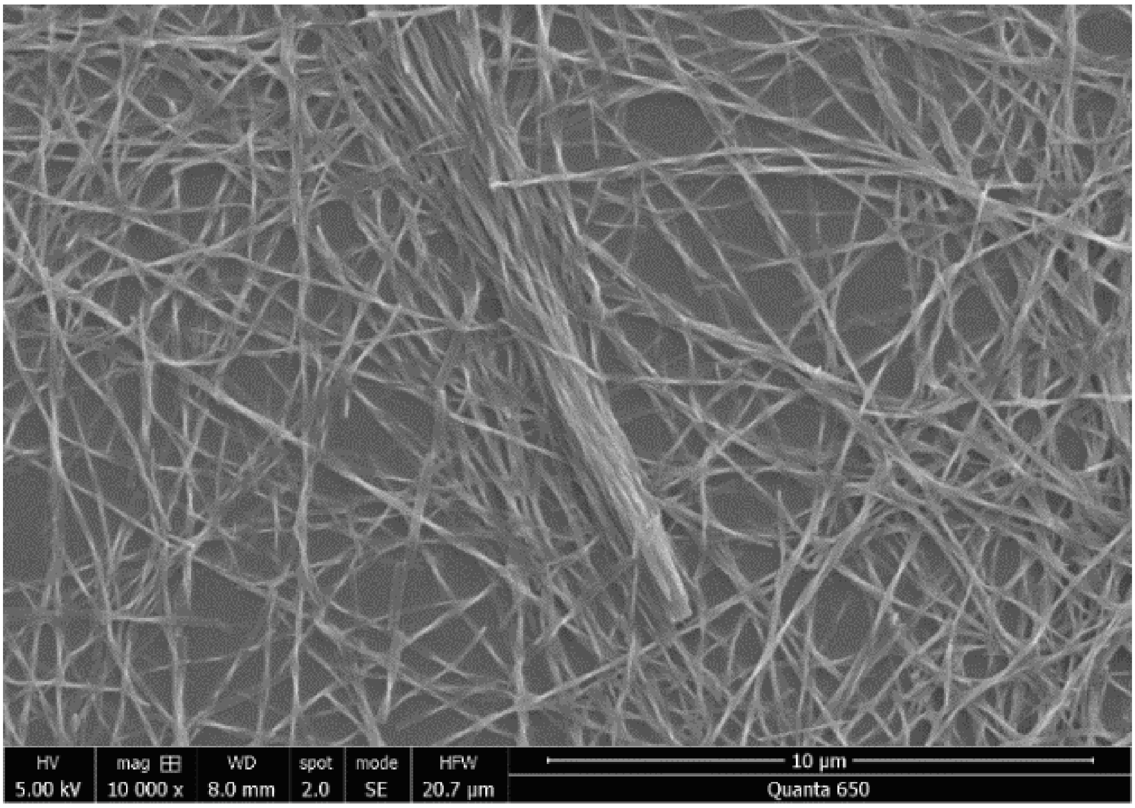

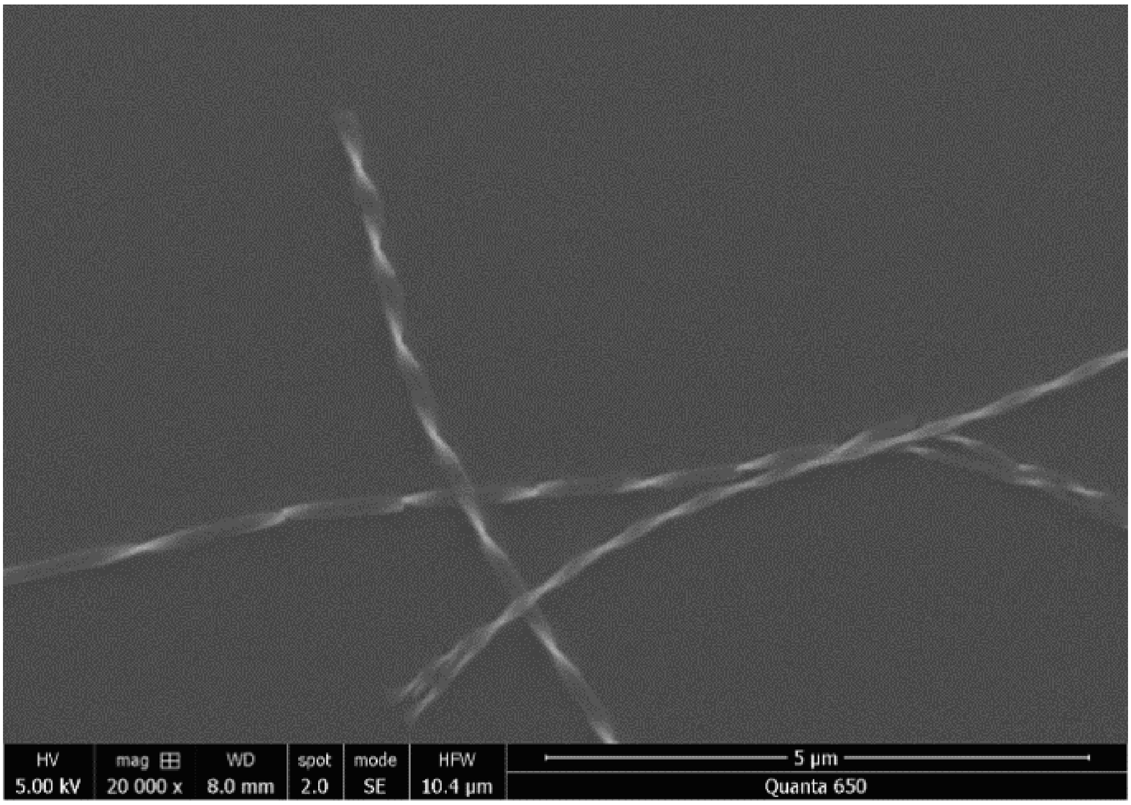

5.29. SEM image of **PhO(*S,S*) EP DPP NH**, grown from an acetone solution under variable temperature conditions showing the general morphology and twisting of the fibres.

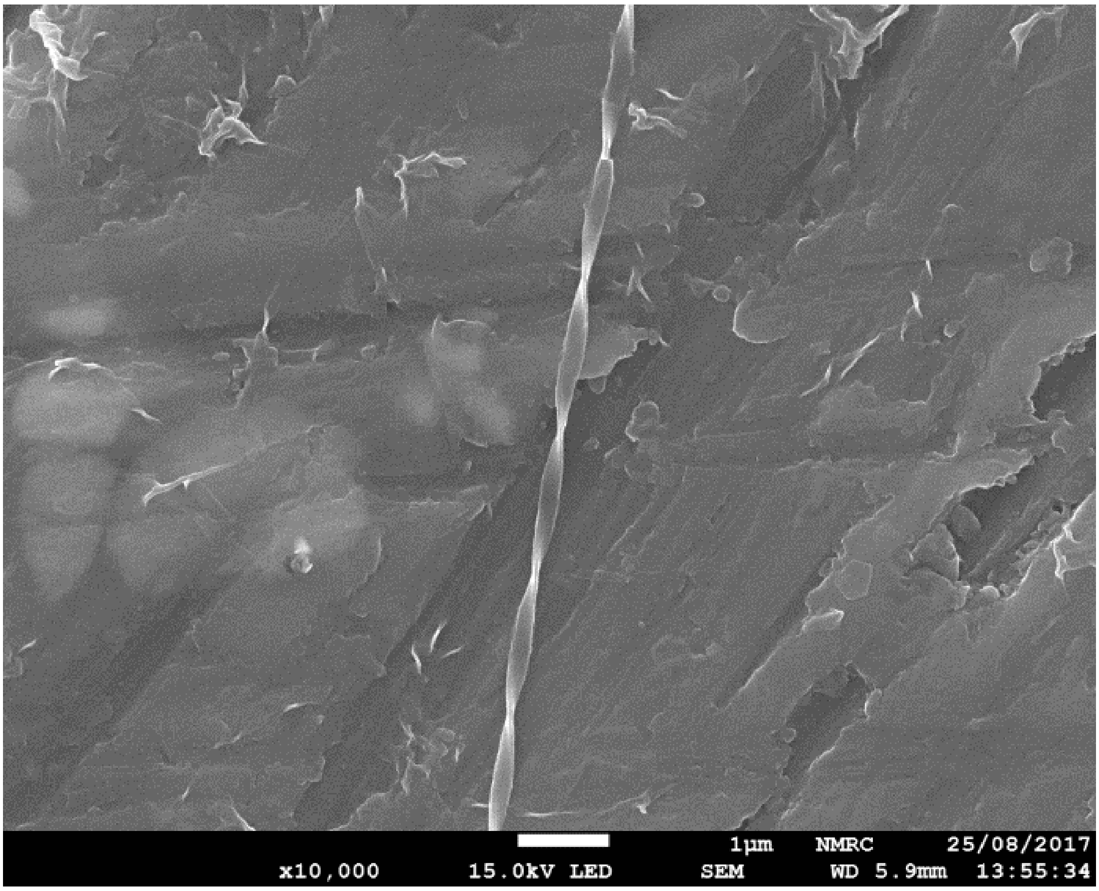

5.30. SEM image of **PhO(R,R) EP DPP NH**, grown from an acetone solution under variable temperature conditions showing the general morphology and twisting of the fibres.

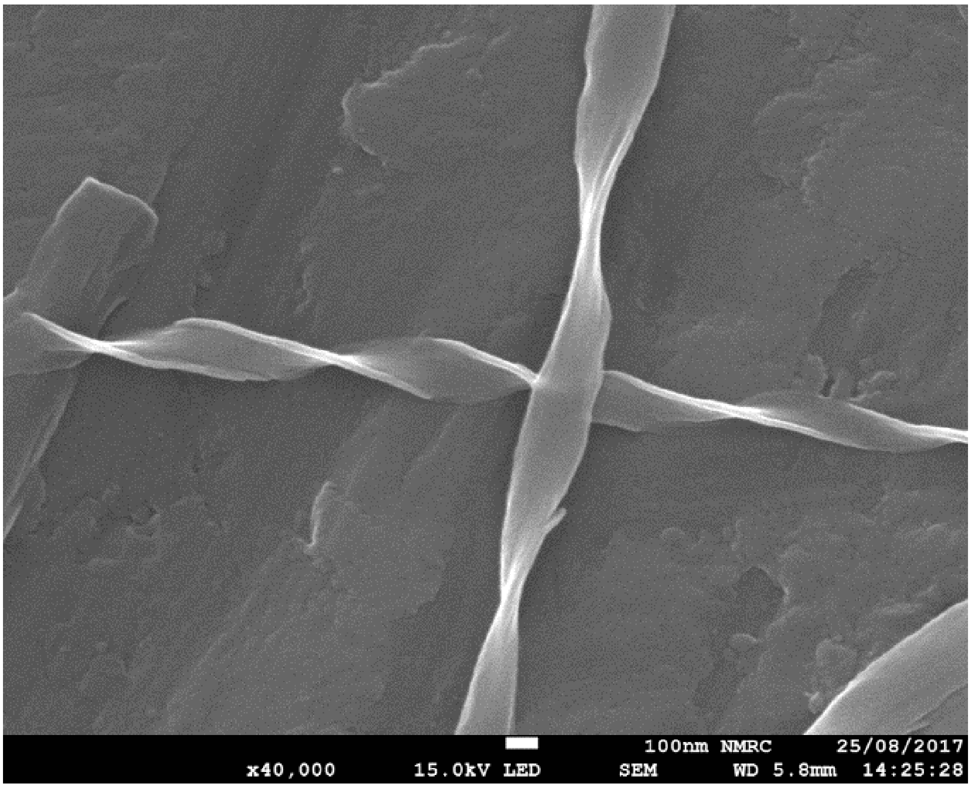

5.31. SEM image of **PhO(R,R) EP DPP NH**, grown from an acetone solution under variable temperature conditions showing the general morphology and twisting of the fibres.

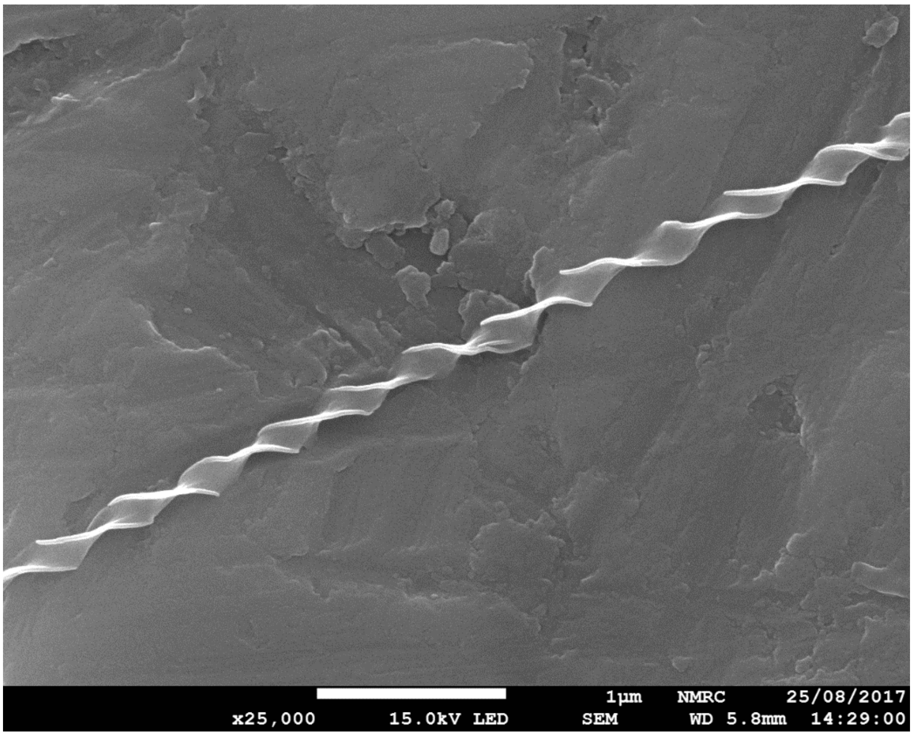

5.32. SEM image of **PhO(S,S) EP DPP NH**, grown from an acetone solution under variable temperature conditions showing the general morphology and twisting of the fibres.

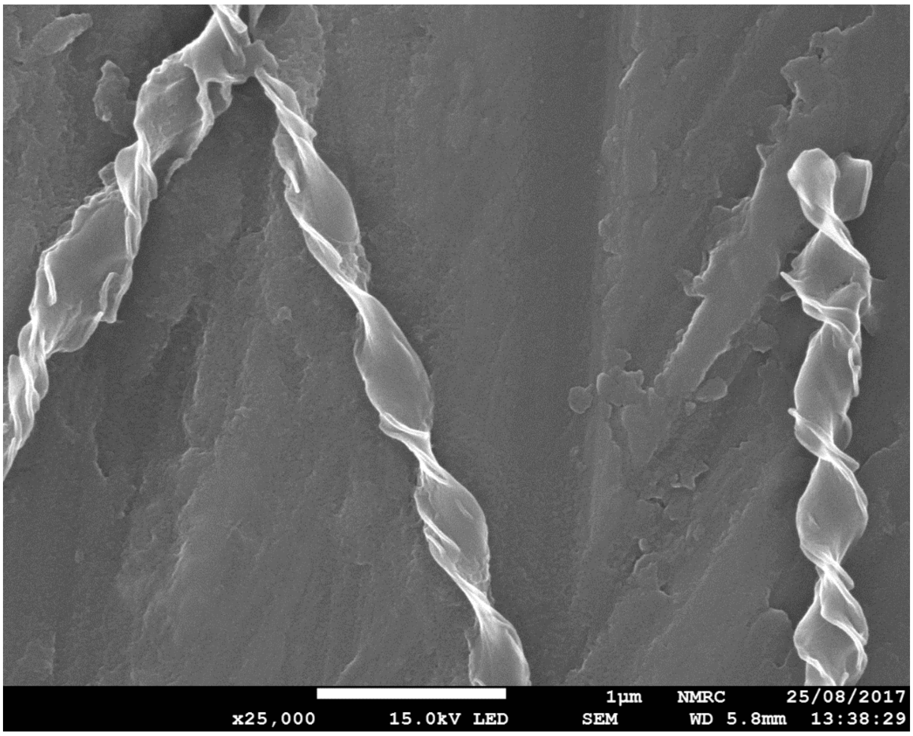

5.33. SEM image of **PhO(S,S) EP DPP NH**, grown from an acetone solution under variable temperature conditions showing the general morphology and twisting of the fibres.

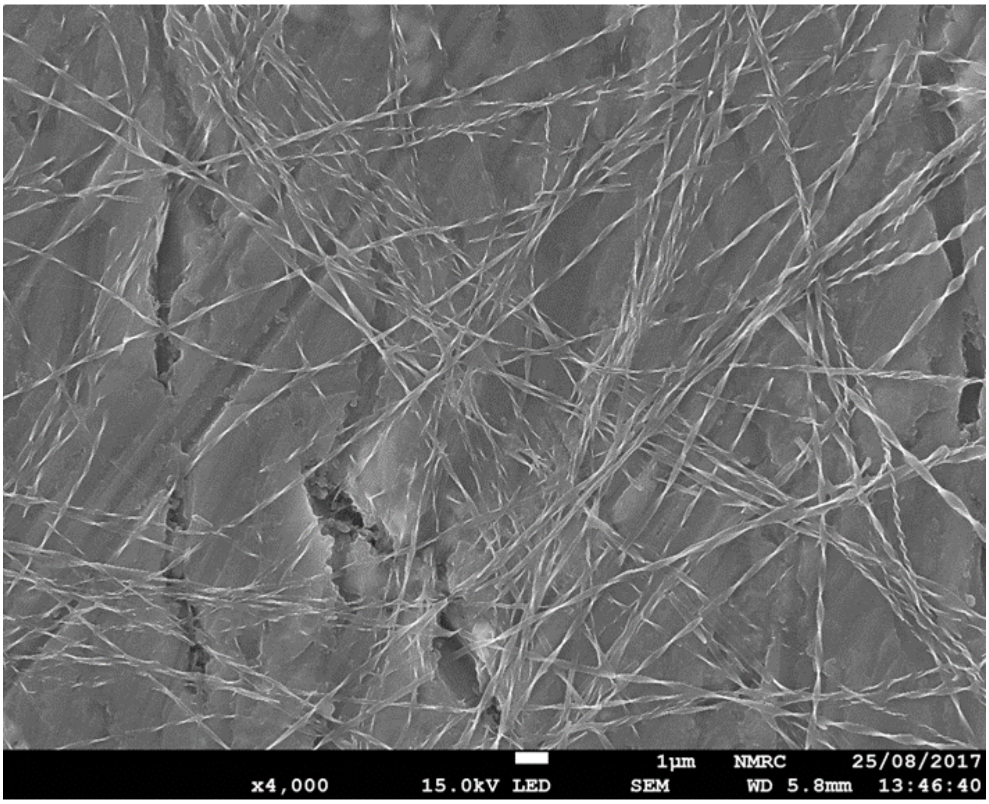

5.34. SEM image of **PhO(R,R) EP DPP NH**, grown from an acetone solution under variable temperature conditions detailing the direction of twisting of each aggregate.

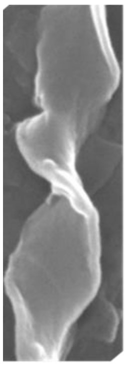

SS

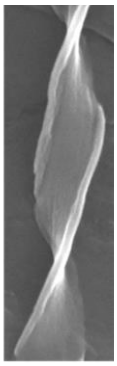

RR

5.35 SEM image of **PhO(S,S) EP DPP NH**, grown from an acetone solution under variable temperature conditions detailing the direction of twisting of each aggregate.

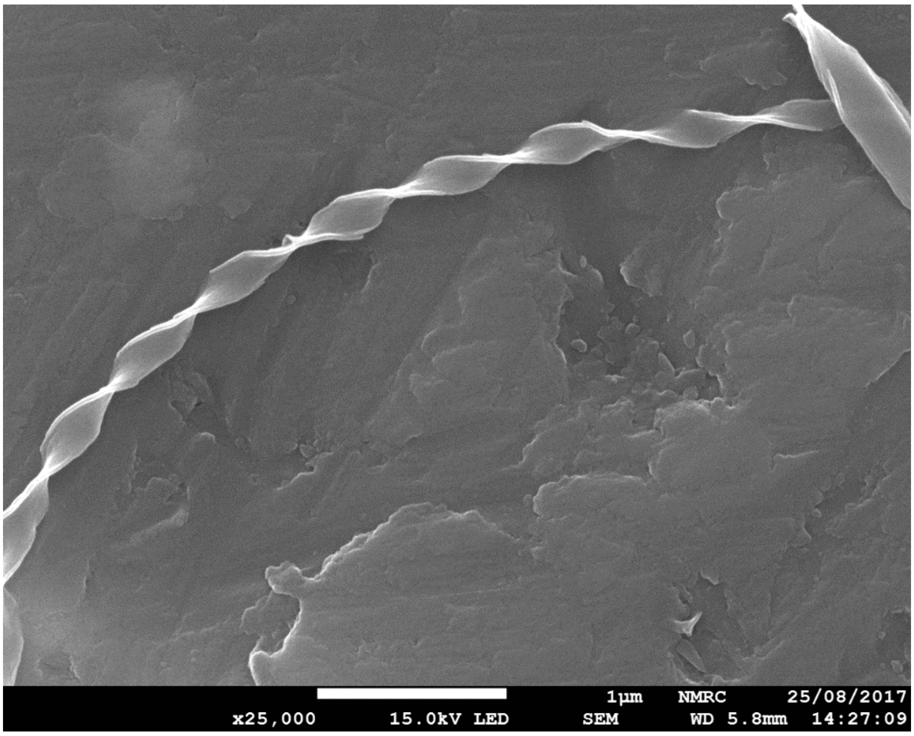

5.36 SEM image of **PhO(*S,S*) EP DPP NH**, grown from an acetone solution under variable temperature conditions detailing the direction of twisting of each aggregate.

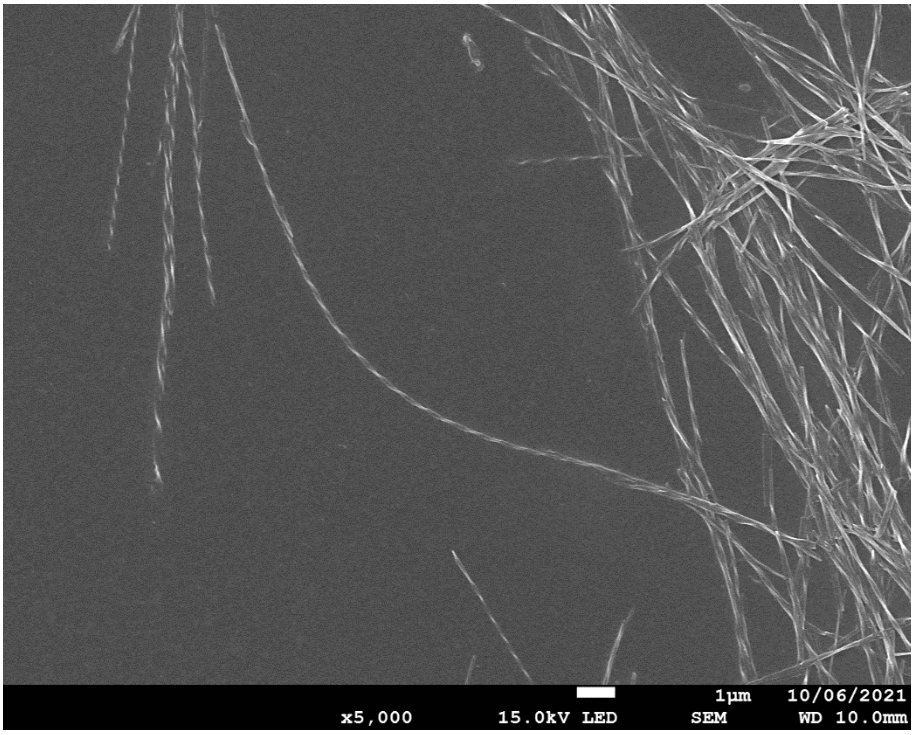

5.37 SEM image of **PhO(*S,S*) EP DPP NH**, grown from an acetone solution under variable temperature conditions detailing the direction of twisting of each aggregate.

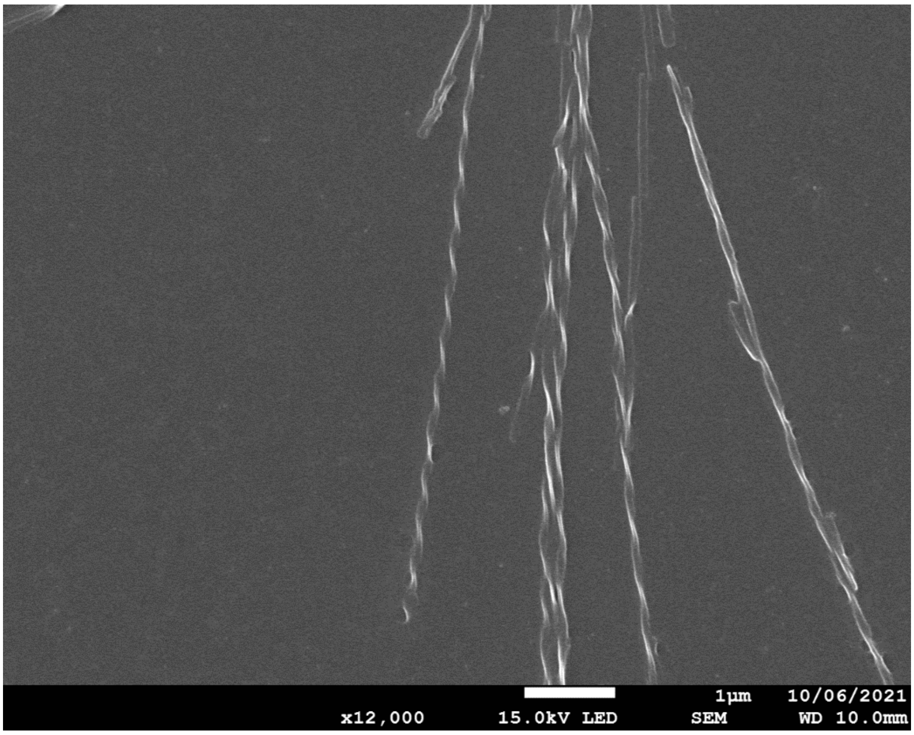

5.38 SEM image of **PhO(*R*,*R*) EP DPP NH**, grown from an acetone solution under variable temperature conditions detailing the direction of twisting of each aggregate.

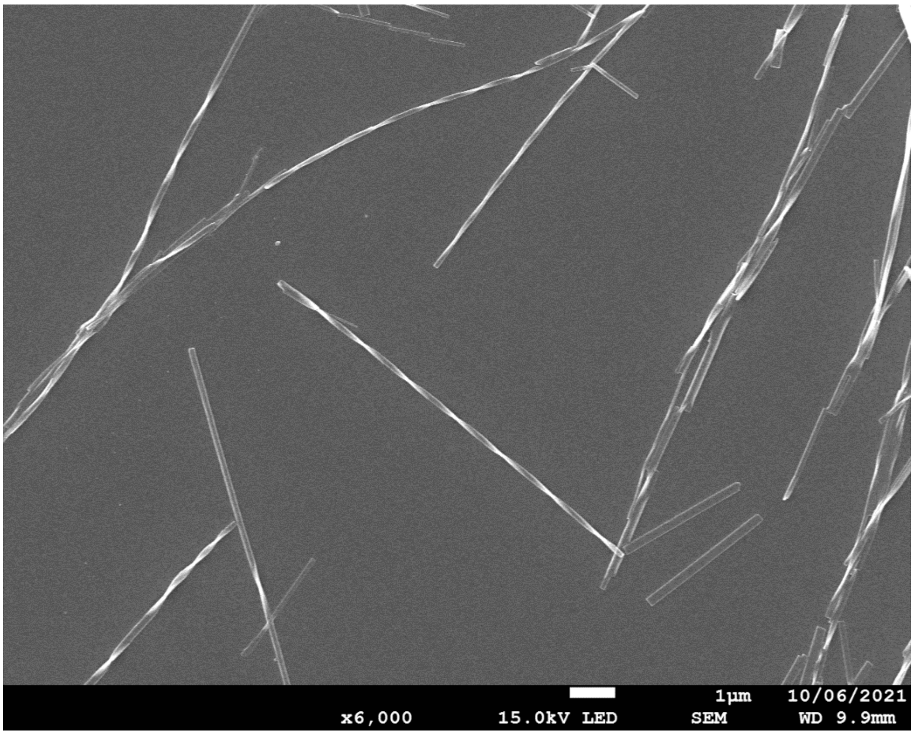

5.39. SEM image of **PhO(S,S) EP DPP NH**, grown from a methanol solution under variable temperature conditions showing the general morphology and twisting of the fibres.

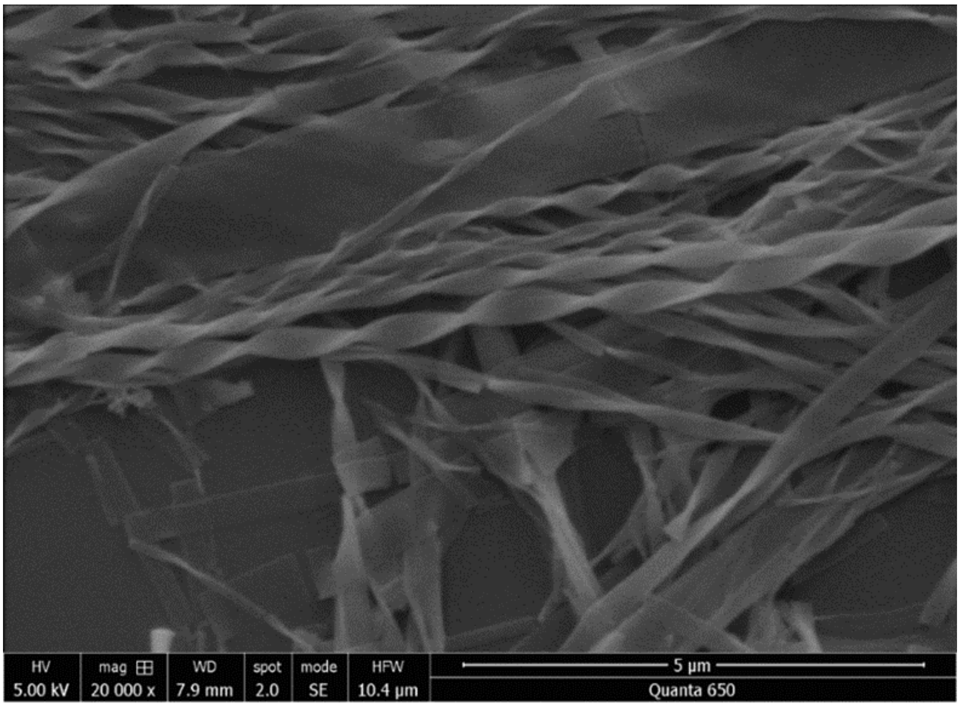

5.40. SEM image of **PhO(R,R) EP DPP NH,** grown from a methanol solution under variable temperature conditions showing the general morphology and twisting of the fibres.

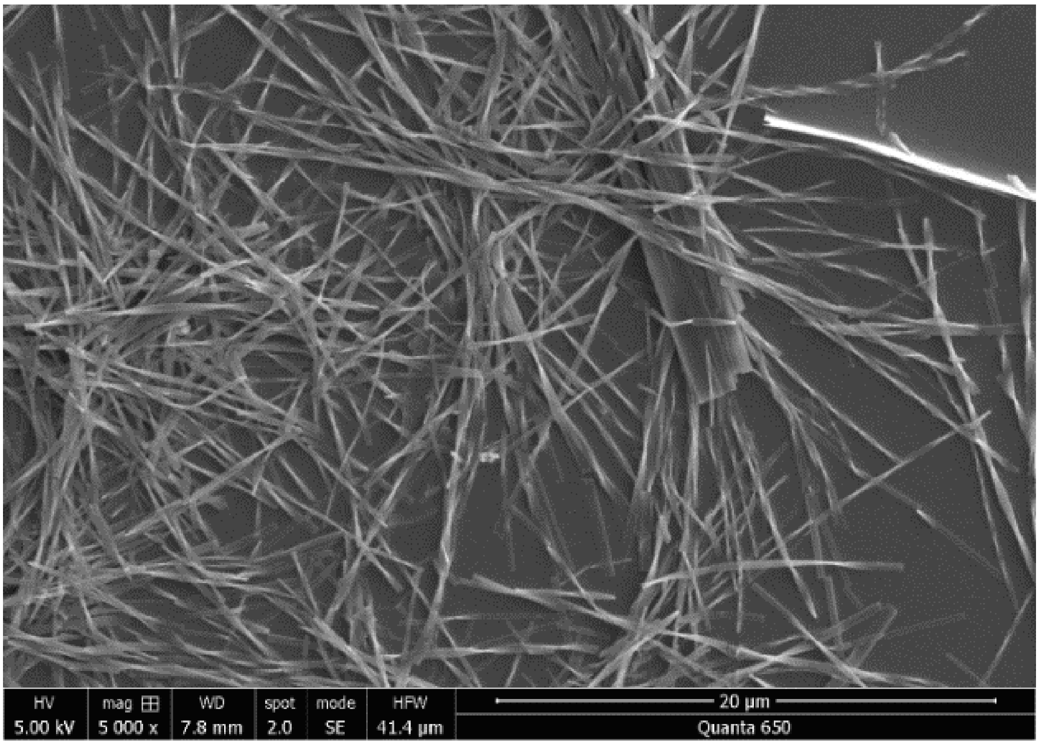

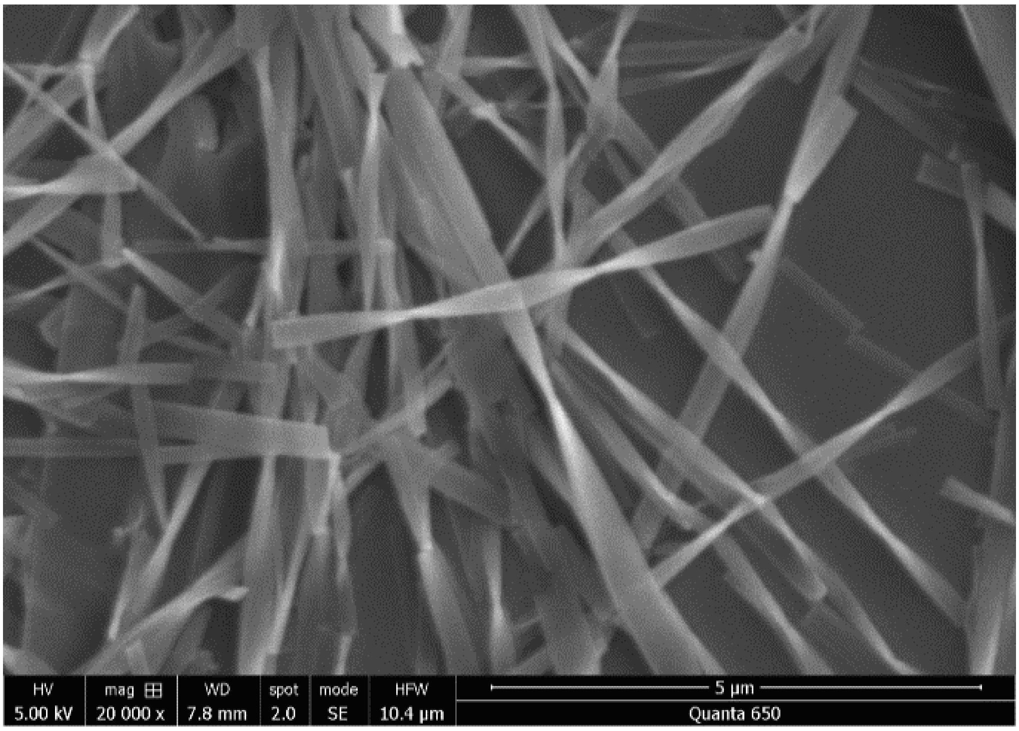

5.41 SEM image of **PhO(*S,S*) EP DPP NH** grown under vapour diffusion conditions showing the general morphology and twisting of the fibres.

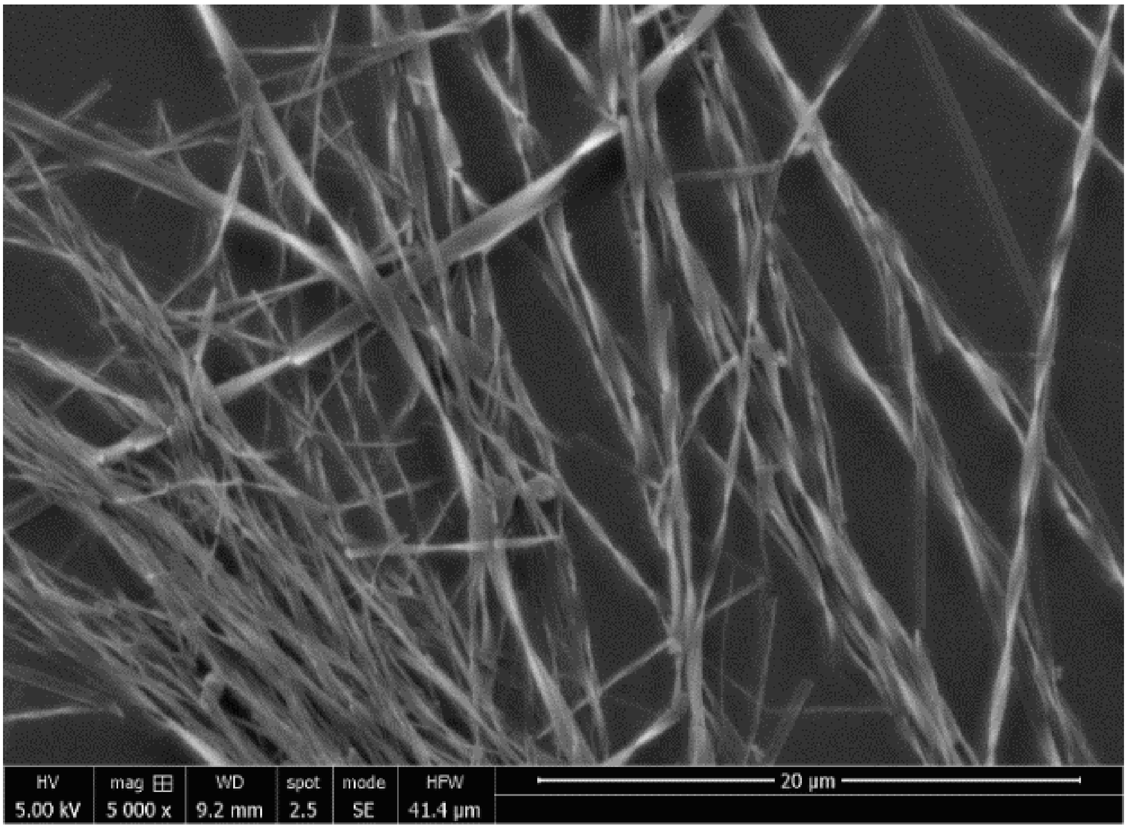

5.42. SEM image of **PhO(*S*,*S*) EP DPP NH** grown under vapour diffusion conditions showing the general morphology and twisting of the fibres.

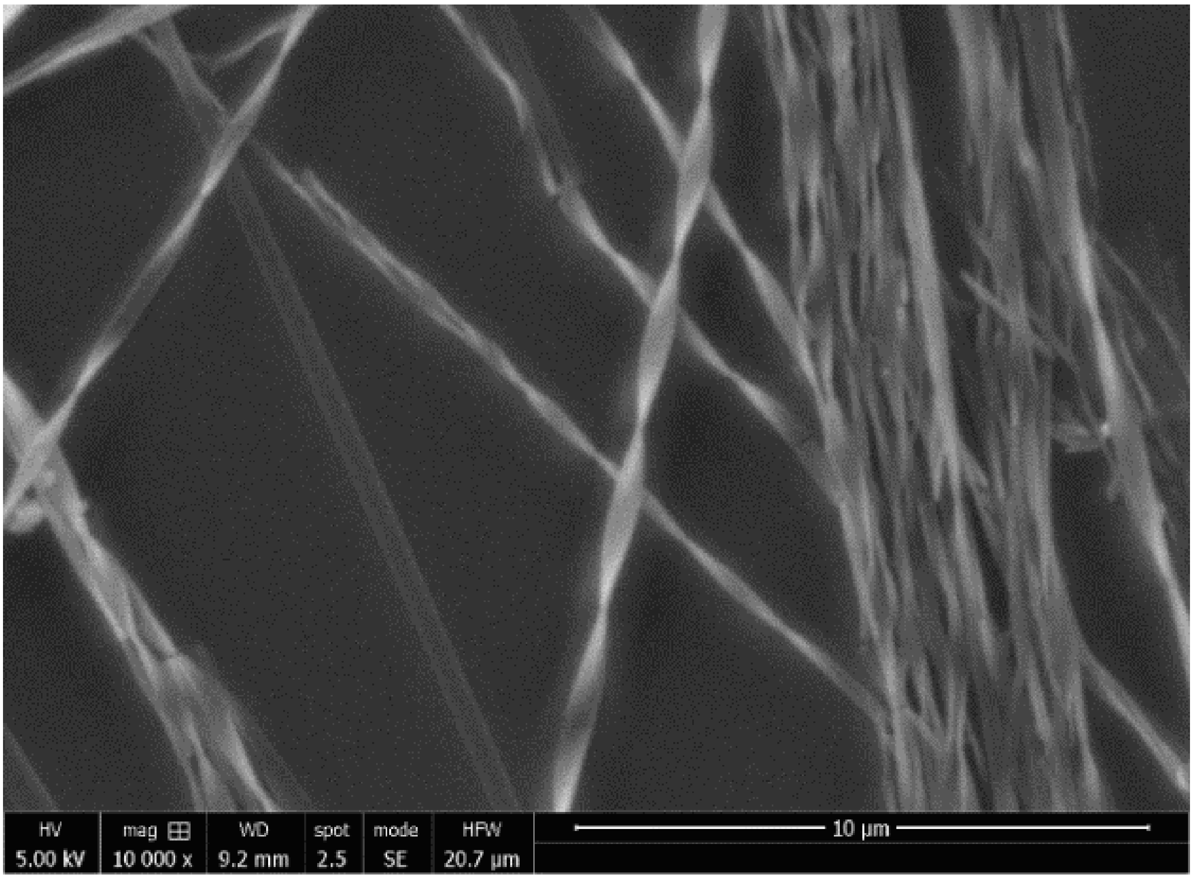

5.43. SEM image of **PhO(*R*,*R*) EP DPP NH** grown under vapour diffusion conditions showing the general morphology and twisting of the fibres.

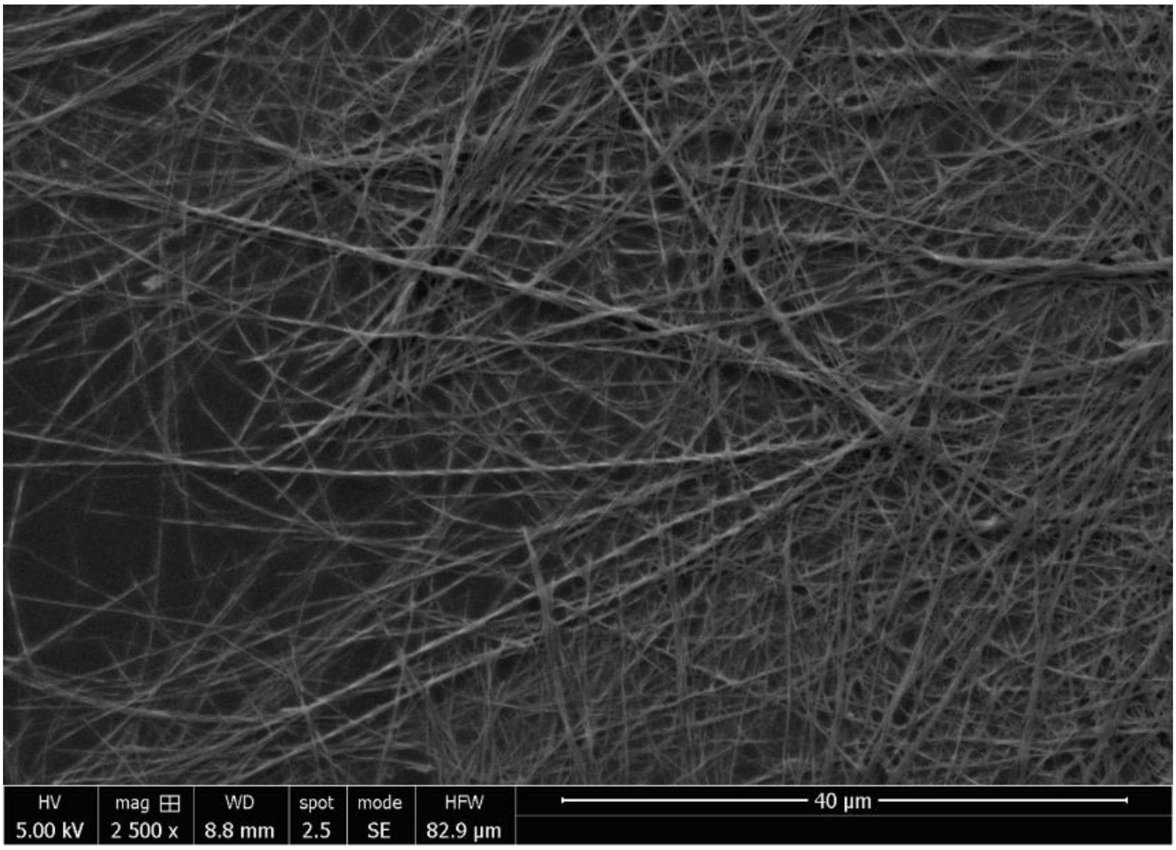

5.44. SEM image of **PhO(*R*,*R*) EP DPP NH** grown under vapour diffusion conditions showing the general morphology and twisting of the fibres.

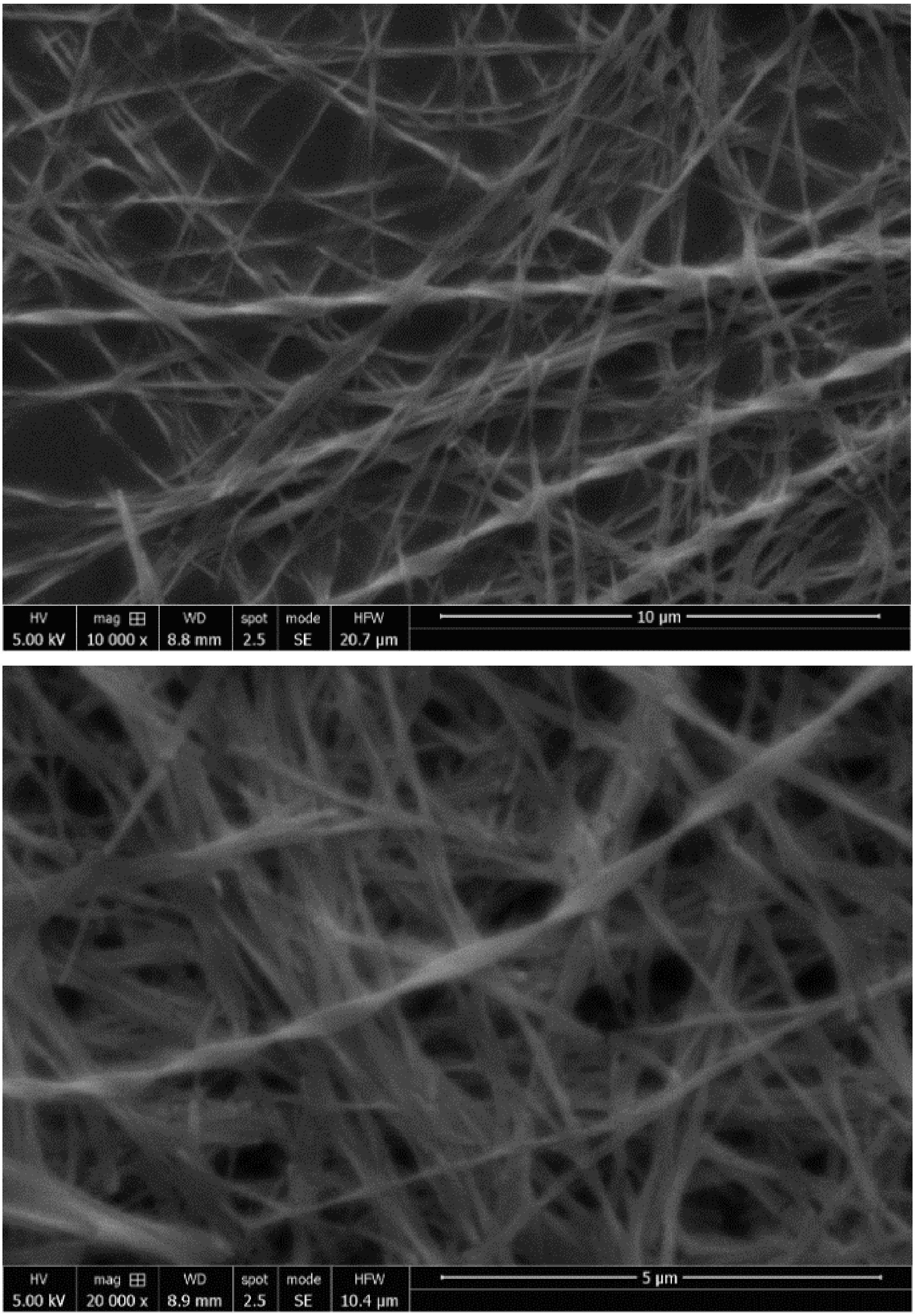

5.45. SEM image of **PhO(*S*,*S*) EP DPP NH**, grown from a methanol solution under variable temperature conditions showing the general morphology and twisting of the fibres.

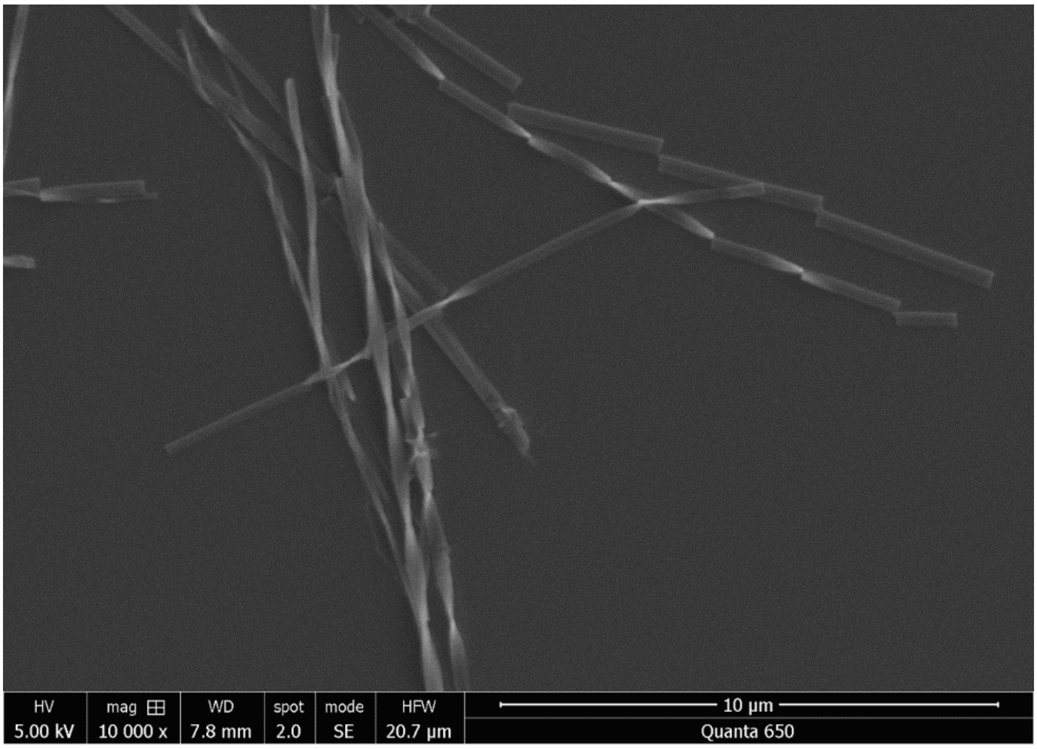

5.46 Correlation between full twist period (2P, 2π rotation, μm) and the smallest crystal size in a cross section (for example, the thickness of a ribbon, h, μm). For each material and condition, the exponent n can be determined from a fit to P = khn. **PhO(*R*,*R*) EP DPP NH** grown from acetone, n=0.47

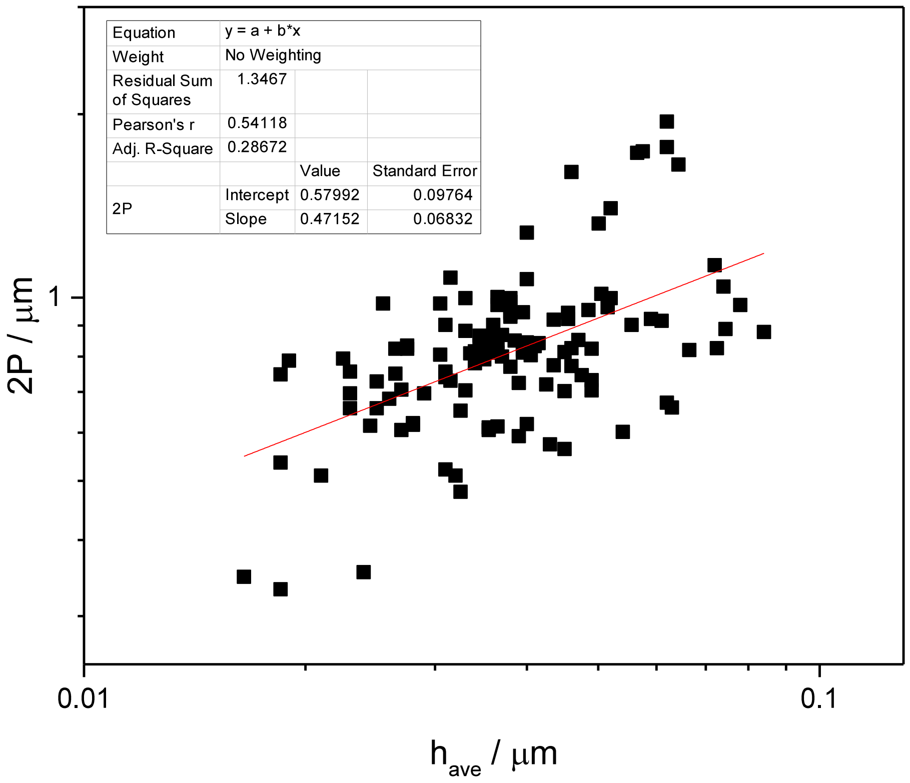

5.47 Correlation between full twist period (2P, 2π rotation, µm) and the smallest crystal size in a cross section (for example, the thickness of a ribbon, h, µm). For each material and condition, the exponent n can be determined from a fit to P = khn. **PhO(*S*,*S*) EP DPP *N*H** – grown from acetone , n=0.49.

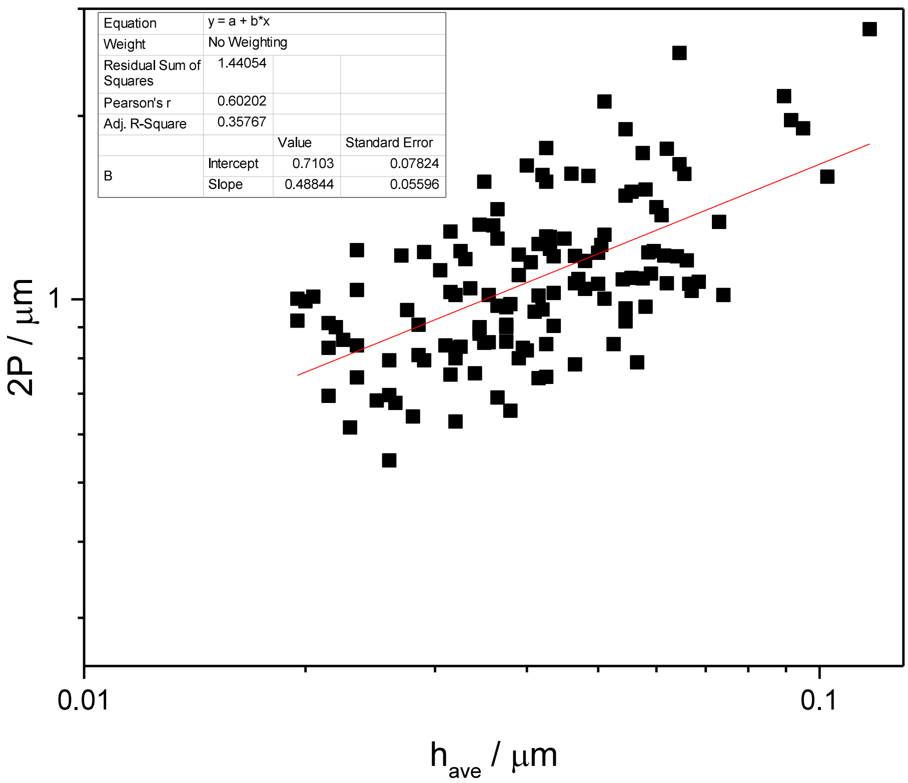

5.48. Correlation between full twist period (2P, 2π rotation, μm) and the smallest crystal size in a cross section (for example, the thickness of a ribbon, h, μm). For each material and condition, the exponent n can be determined from a fit to P = khn. grown from acetone,. The line corresponds to 2P < 103πh, below which (at least) partial relaxation of stress can relax in the twisted crystals.

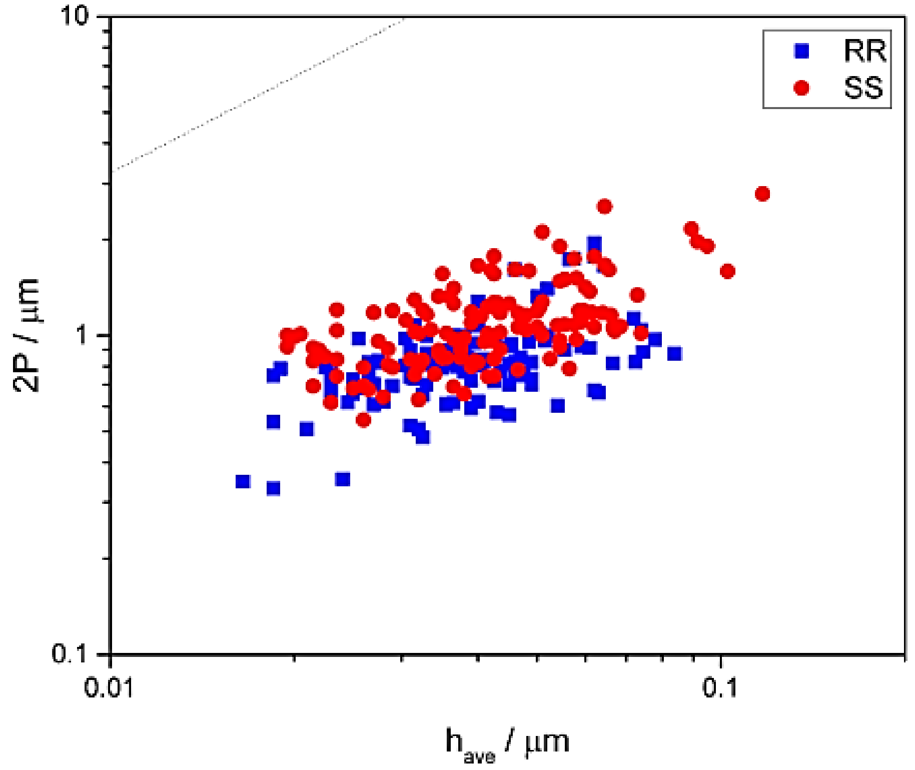

5.49 IR spectra of **PhO(*S*,*S*) EP DPP *N*H** compared carbonyl region

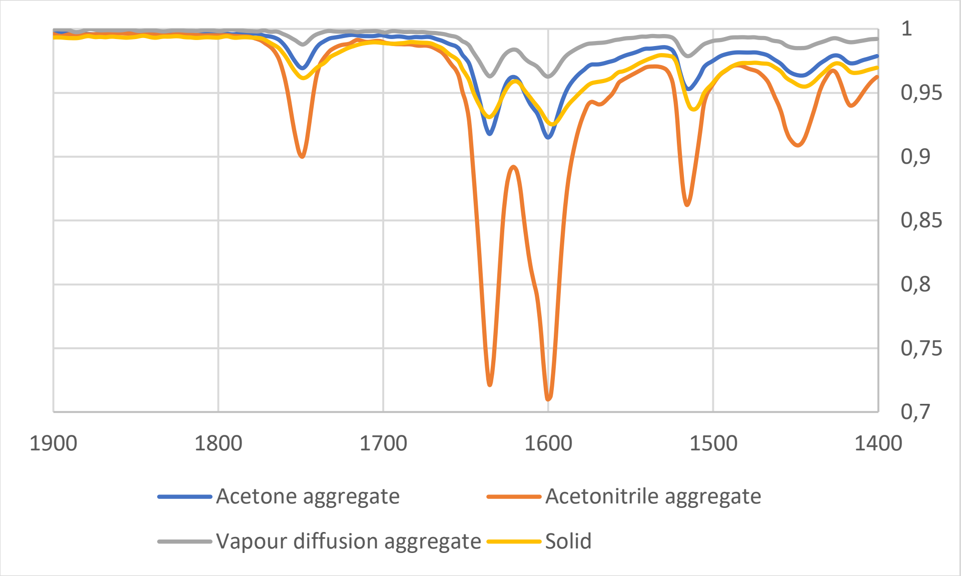

5.50 IR spectra of **PhO(*S*,*S*) EP DPP *N*H** compared NH region

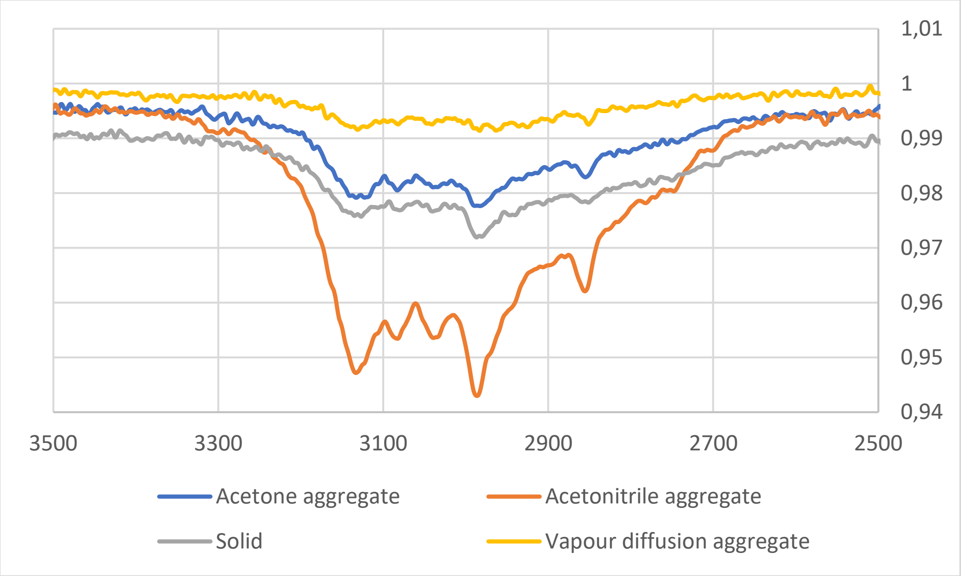

5.51 Absorbance spectra of **PhO(*R,R*) EP DPP *N*H (blue) and PhO(*S,S*) EP DPP *N*H** (red) in 0.5 mmol solutions of THF giving monomeric Circular Dichroism spectra

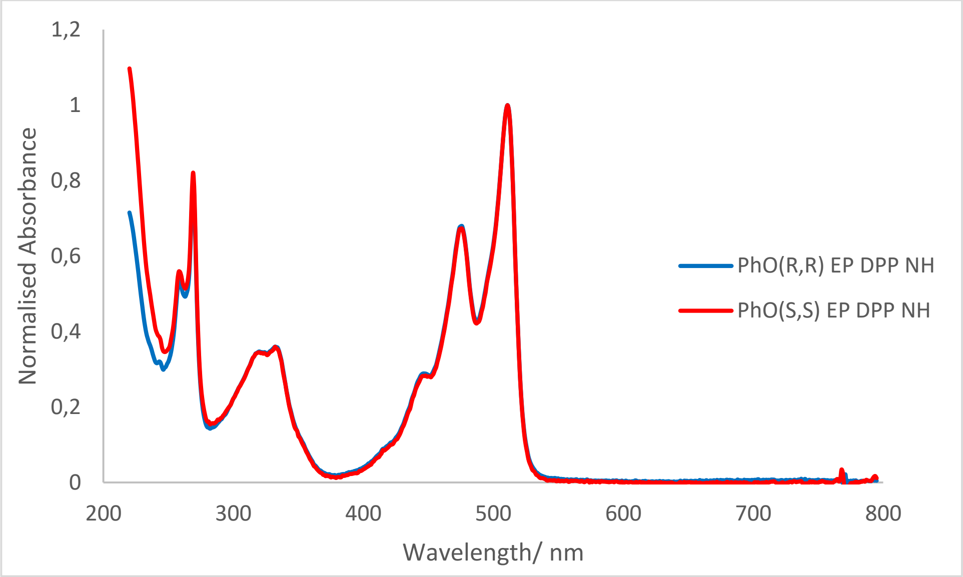

5.52 Circular Dichroism spectra of **PhO(*R,R*) EP DPP *N*H** (black) and **PhO(*S,S*) EP DPP *N*H** (red) in 0.5 mmol solutions of THF.

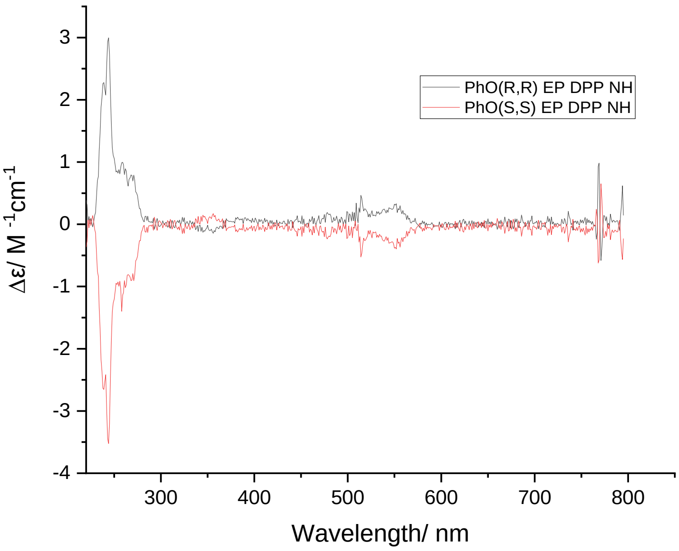

5.53. CD spectrum of **PhO(R,R) EP DPP NH** and **PhO(S,S) EP DPP NH** aggregates formed from an acetone solution displaying opposite chirality.

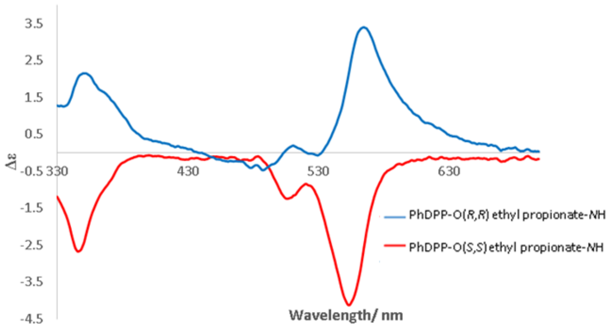

5.54. CD spectrum of **PhO(R,R) EP DPP NH** and **PhO(S,S) EP DPP NH** in acetone displaying loss of CD signal upon heating, indicating dissolution of aggregates to monomeric species.

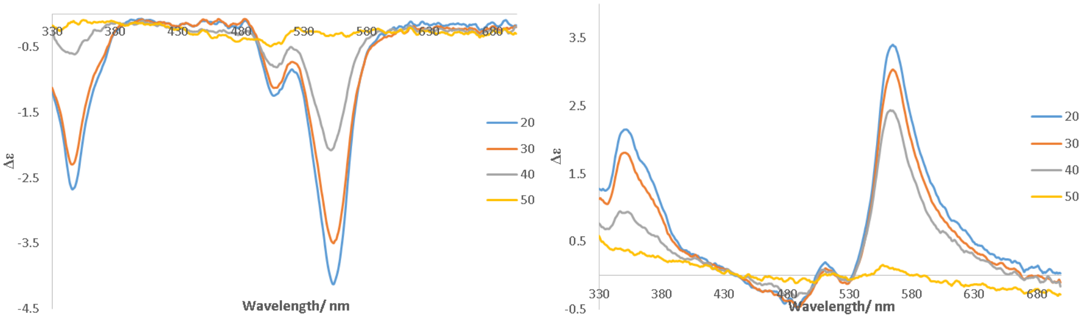

5.55. CD spectrum of **PhO(S,S) EP DPP NH** in acetone showing the signal doesn't return on cooling at 2.5 ^{O}C/ min and requires slower cooling rates, suggesting kinetic controlled growth.

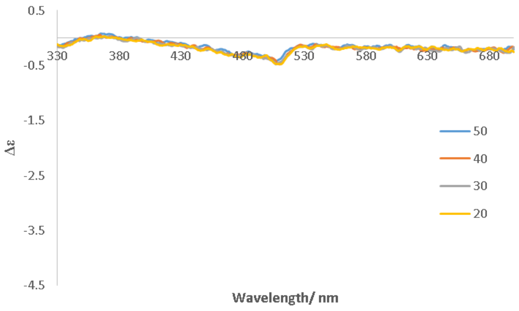

5.56 Plot of CD signal at 552 nm of **PhO(S,S) EP DPP NH** in acetone giving indication of supramolecular polymerisation mechanism

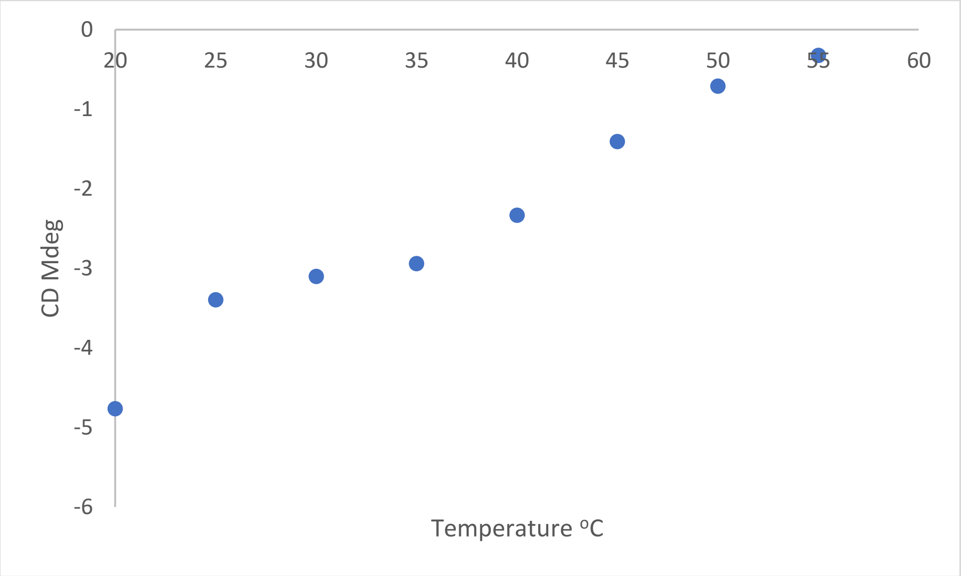

5.57 Absorbance spectra of **PhO(S,S) EP DPP NH** in acetone displaying loss of J aggregate signal upon heating, indicating dissolution of aggregates to monomeric species.

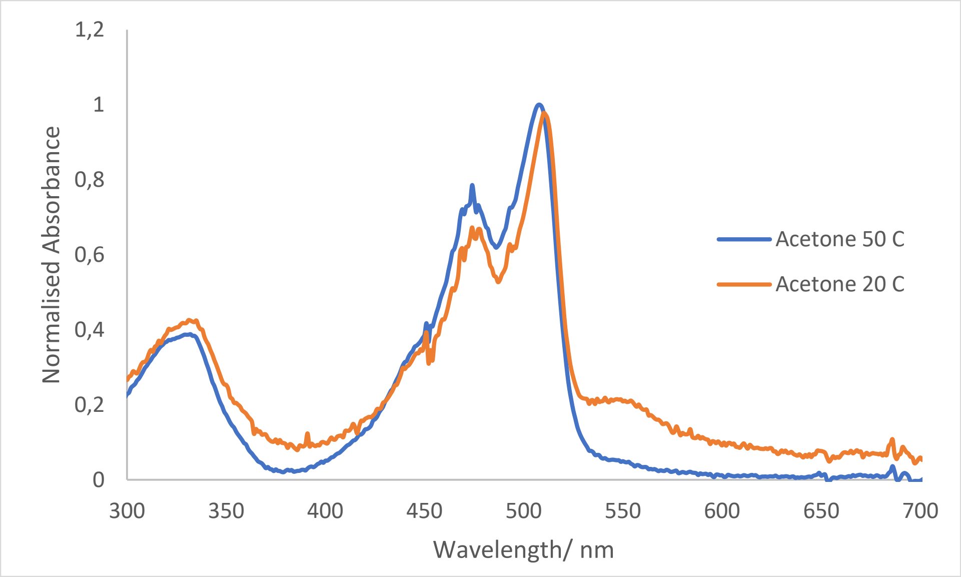

5.58 Absorbance spectra of **PhO(*R*,*R*) EP DPP *N*H (blue) and PhO(*S*,*S*) EP DPP *N*H** (red) in 0.35 mmol solutions of ethanol at 75 °C

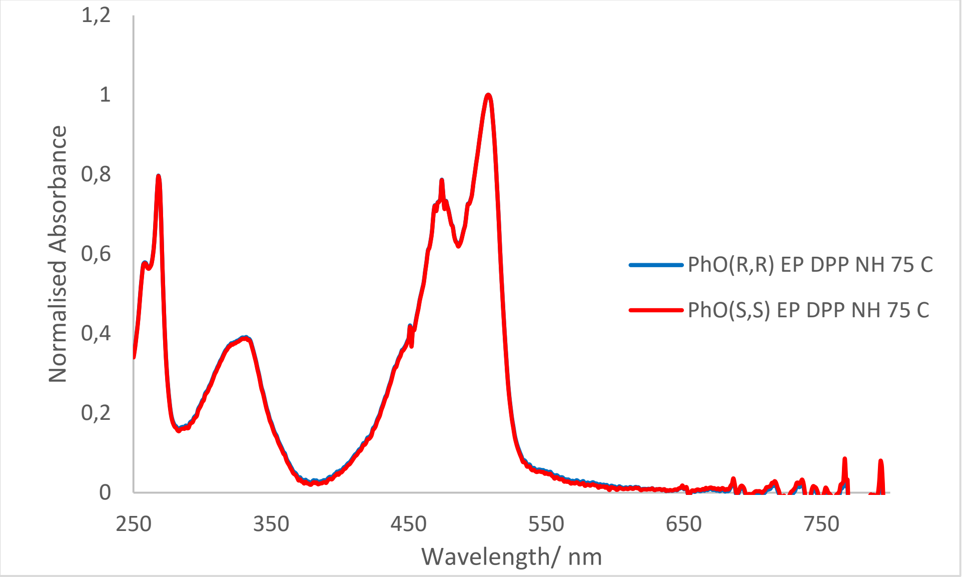

5.59 Circular Dichroism spectra of **PhO(*R*,*R*) EP DPP *N*H (blue) and PhO(*S*,*S*) EP DPP *N*H** (red) in 0.35 mmol solutions of ethanol at 75 °C.

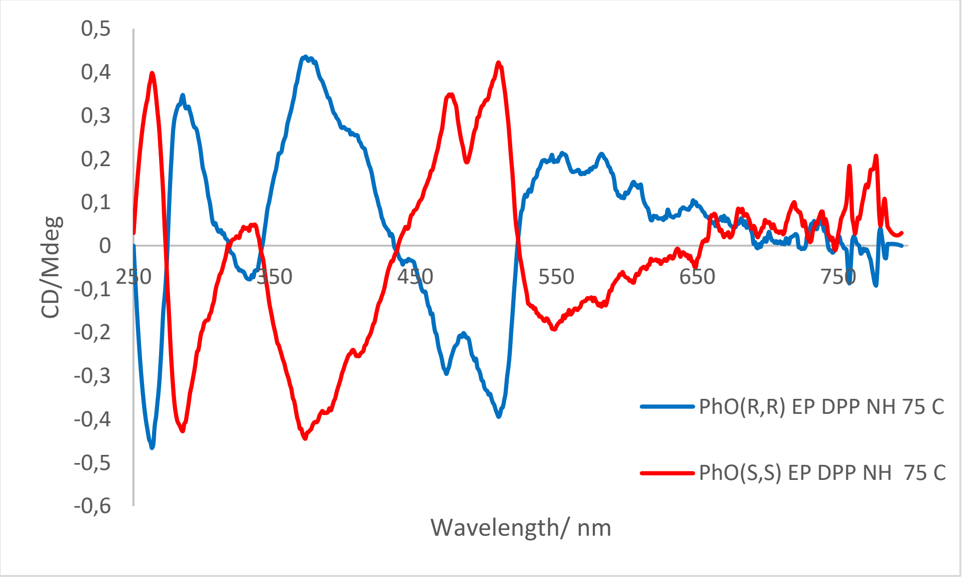

5.60 Absorbance spectra of **PhO(*R,R*) EP DPP *N*H (blue) PhO(*S,S*) EP DPP *N*H** (red) in 0.35 mmol solutions of ethanol at 5 °C

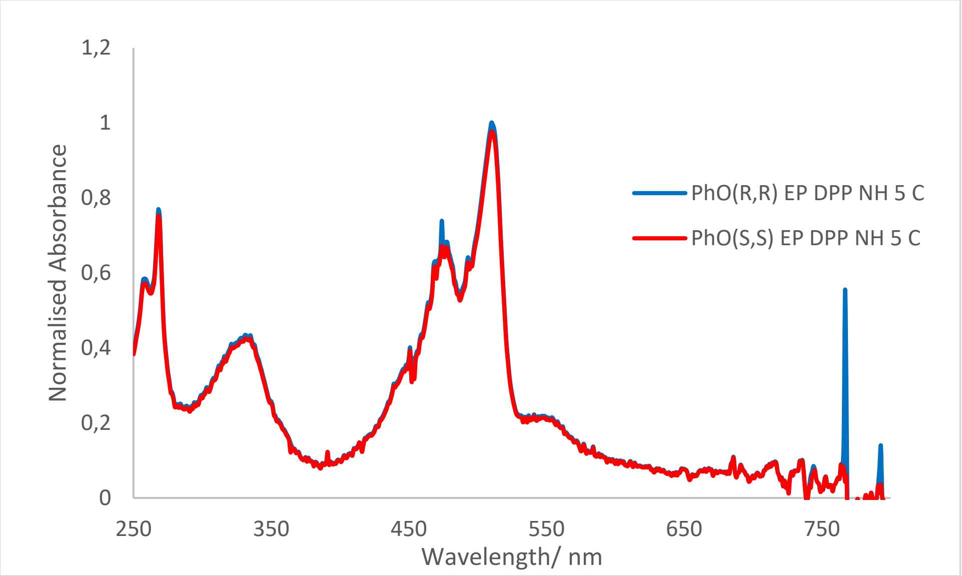

5.61 Emission spectra of of **PhO(*R,R*) EP DPP *N*H** (Blue) and **PhO(*S,S*) EP DPP *N*H** (red) in 0.35 mmol solutions of ethanol.

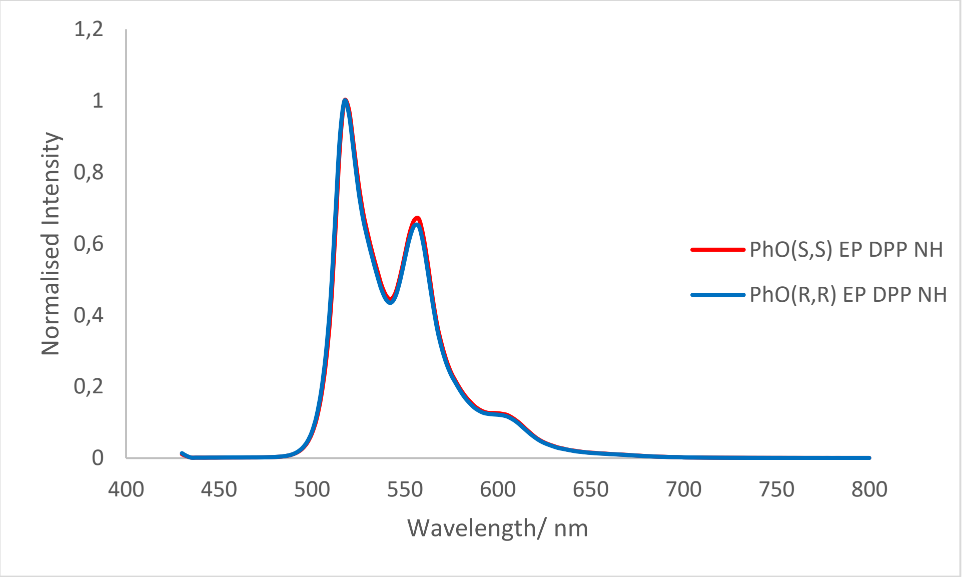

5.62 Illustration of 5 x 5 grid array used when scanning **PhO(*R*,*R*) EP DPP *N*H** films, showing a step size of 50 µm (left). The areas illuminated by the 50 µm beam are depicted as yellow circles.

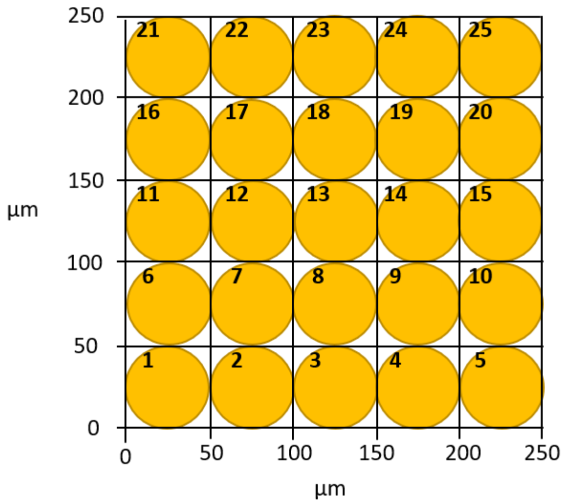

5.63: Absorbance spectra of 25 scans and average (blue dotted line) of a drop cast film from a 2.00 mmol solution of **PhO(*R*,*R*) EP DPP** in THF.

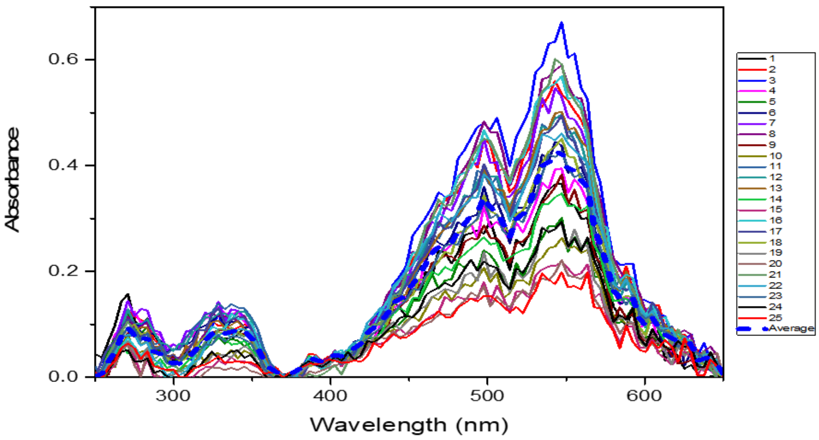

5.64. Averaged CD spectra averages of 25 scans at 20 °C of a drop cast film of **PhO(*R*,*R*) EP DPP *N*H** and when it has been flipped by 180 °.

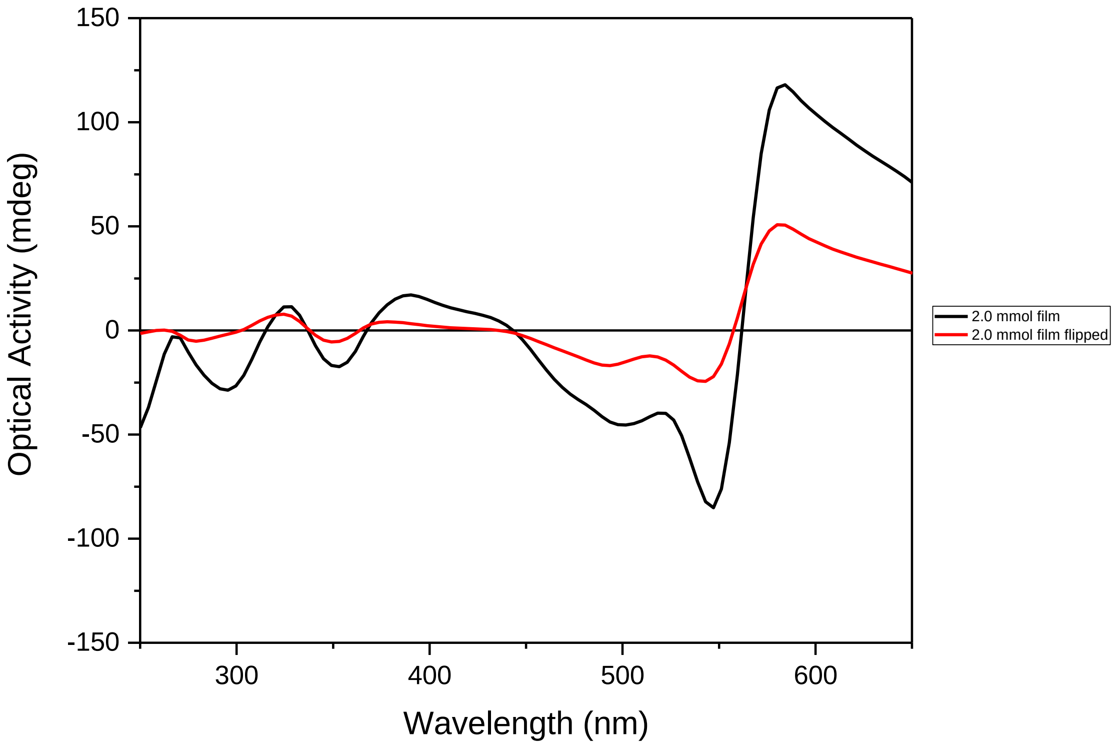

5.65 Self-assembled monolayer of **PhO(*S,S*) EP DPP NH** formed from THF solution with phenyl octane droplet on HOPG (V_{bias} = -700 mV I_{set} = 300 pA)

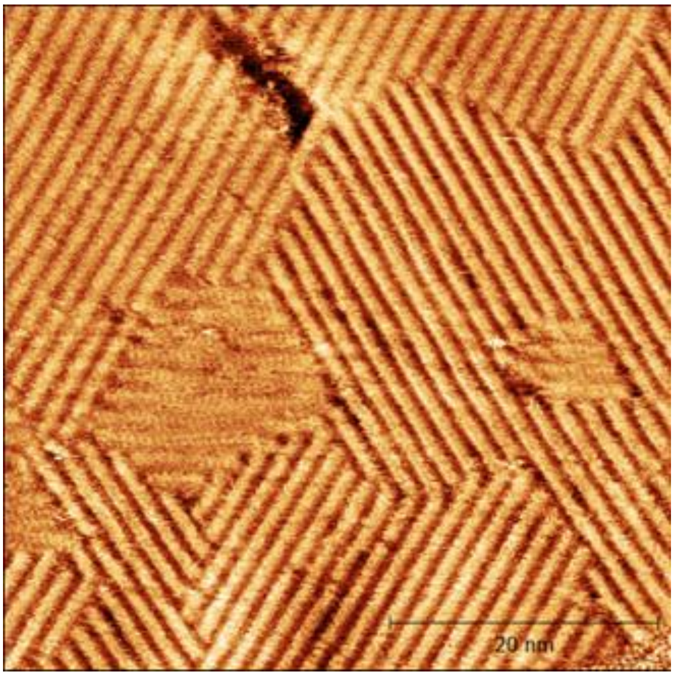

5.66*.* Self-assembled monolayer of **PhO(*S,S*) EP DPP NH** formed from THF solution on HOPG with phenyl octane droplet showing defect in packing not representative of sample (V_{bias} = -700 mV I_{set} = 300 pA)

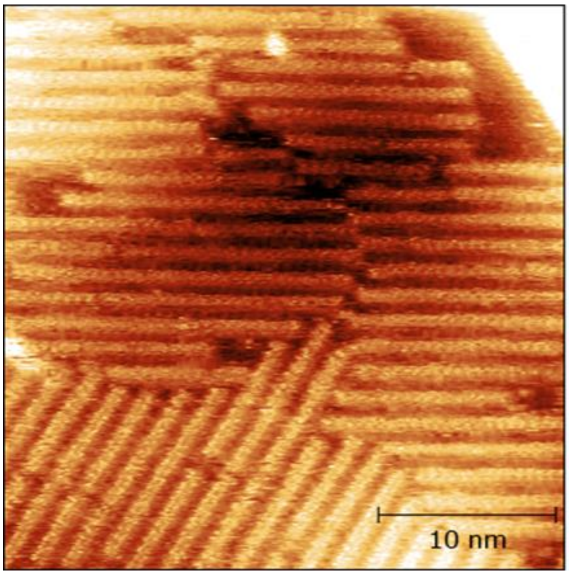

5.67 Proposed stacking orientation of **PhO(*S*,*S*) EP DPP NH** on HOPG driven by hydrogen bonding.

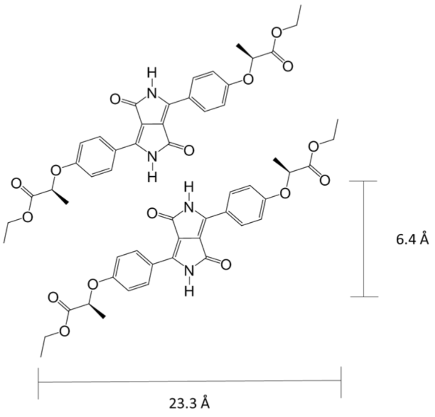

5.68 Several chronological images displaying the dynamic nature of the self-assembled monolayer formed from **PhO(*S*,*S*) EP DPP NH** on HOPG.

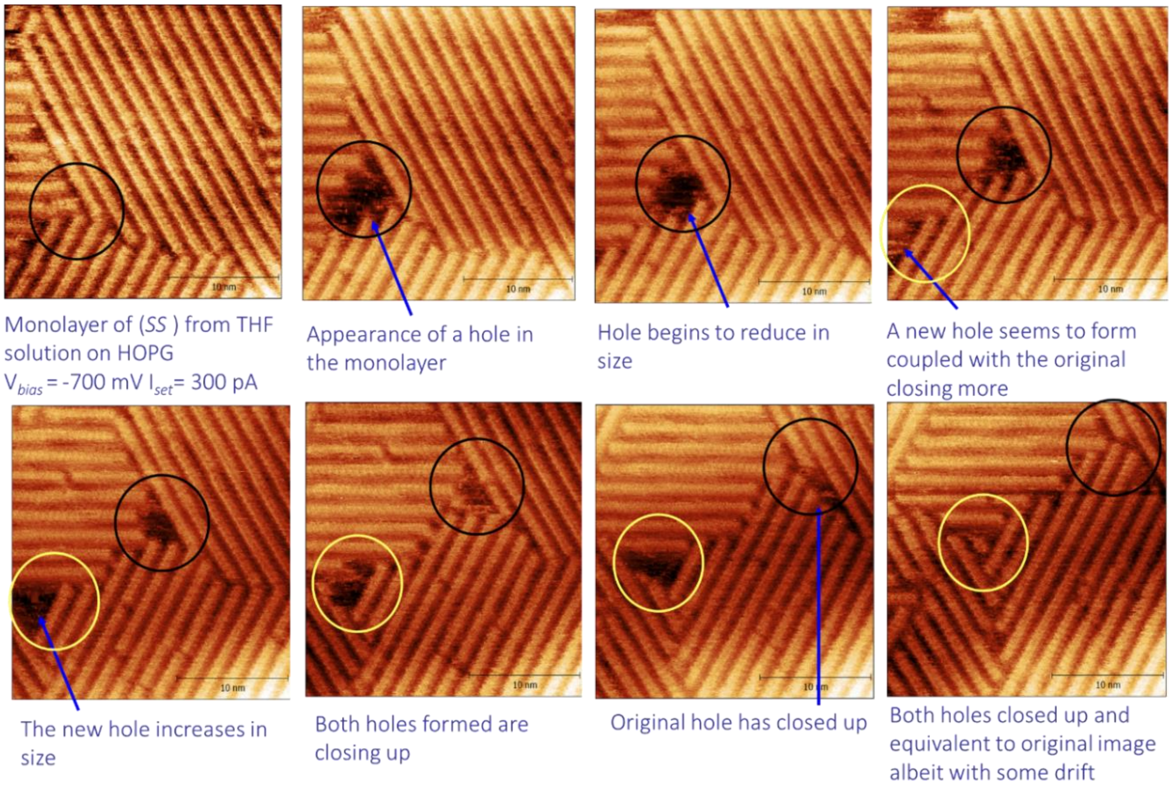

Supplement: Supplementary file 1 — Data S1. Supporting Information. [file CHIR-35-281-s001.pdf]
